# Supplementary material for: Mobile-UI-Repair: a deep learning based UI smell detection technique for mobile user interface
Source: PeerJ Comput Sci. 2024 May 16;10:e2028. doi: 10.7717/peerj-cs.2028 (PMC11157604; doi:10.7717/peerj-cs.2028)
Supplement: Supplemental Information 1 — Use main file UI repair [file peerj-cs-10-2028-s001.zip › MUI Repair code and Data/UI images/Bug Report-1.docx]

**Bug Report**

**Summary:**

My List title is overlapping with the add to my list guidelines text

**Bug/Error Type: UI (**Text overlapping)

**Steps to reproduce:**

- Login the user so that no content is added to My List already
- Go to settings by clicking More button at the bottom navigation bar
- Click on My List option from the setting’s menu

**Actual Result:**

The title text My List is overlapping with the information text below.

**Expected Result:**

My List Title text should appear above the general guidelines text to add to my list so that user can read it easily.

**Screenshot:**

**
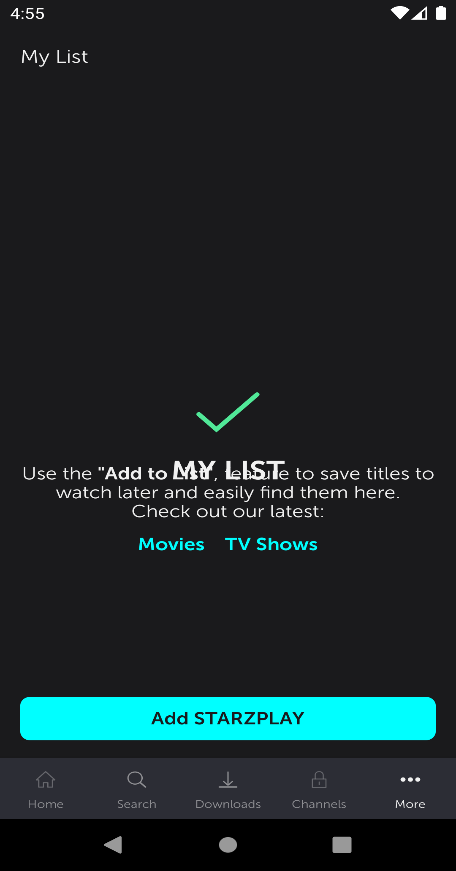
**

**Reporter:** Asif Ali

**Assigned to:** Kaleem Bajwa

**Platform:** Android

**Affects Version:** 6.5.1

**Fix Version:** 6.5.2

**Priority:** Low (Text overlapping hindering the user to follow the guidlines**)**

**Devices:** Android Mobile and Tablet
