# Supplementary figures and images for: Mobile-UI-Repair: a deep learning based UI smell detection technique for mobile user interface
Source: PeerJ Comput Sci. 2024 May 16;10:e2028. doi: 10.7717/peerj-cs.2028 (PMC11157604; doi:10.7717/peerj-cs.2028)

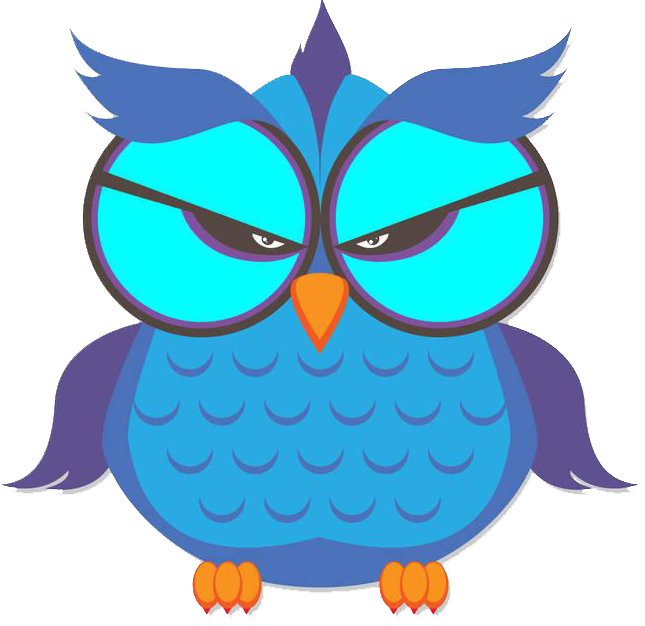

Supplement: Supplemental Information 1 — Use main file UI repair [file peerj-cs-10-2028-s001.zip › MUI Repair code and Data/apple-touch-icon.png]

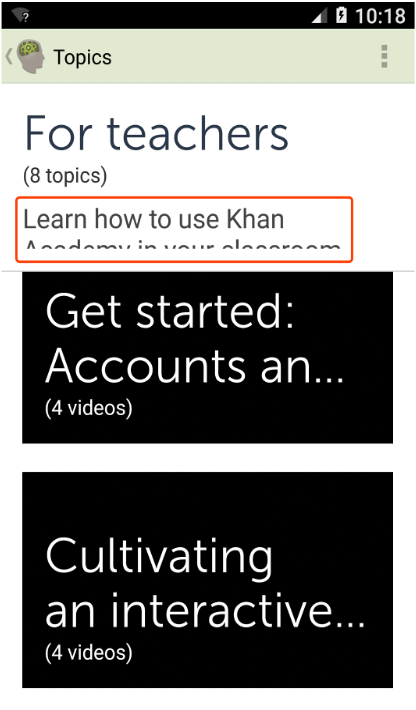

Supplement: Supplemental Information 1 — Use main file UI repair [file peerj-cs-10-2028-s001.zip › MUI Repair code and Data/component Occlusion/1.jpg]

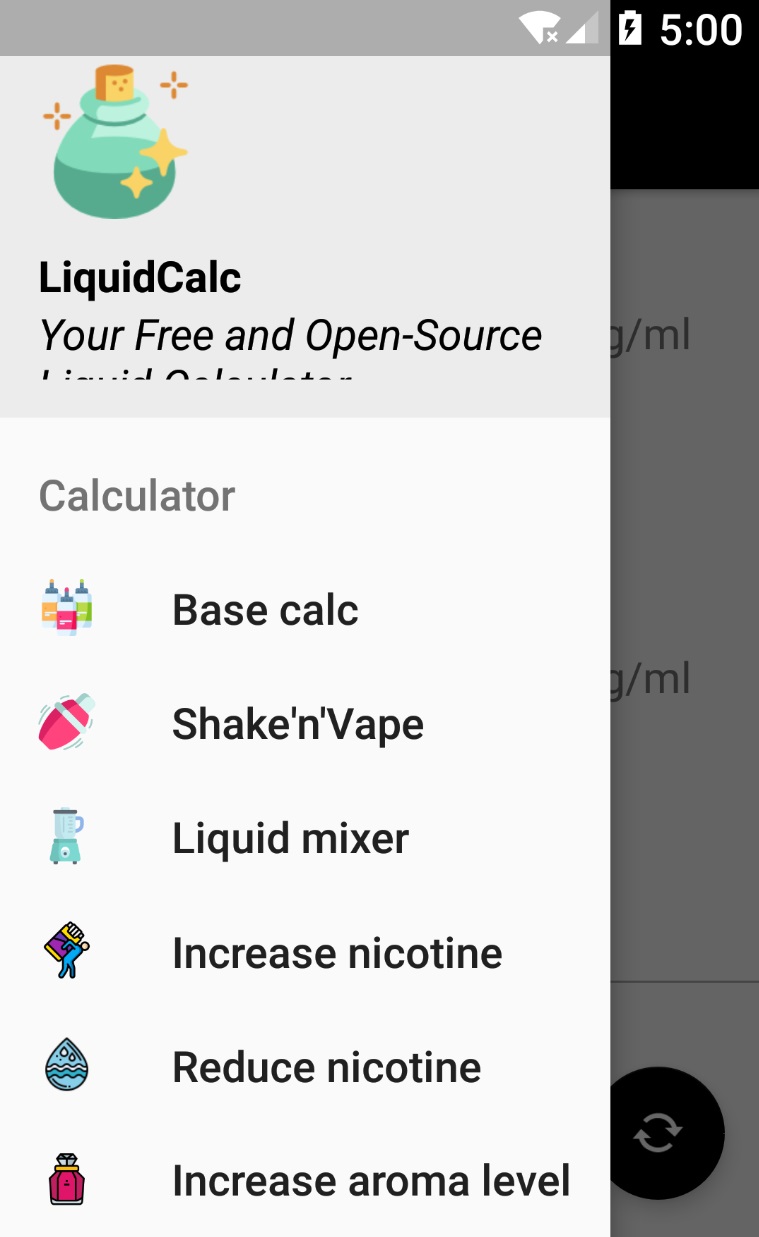

Supplement: Supplemental Information 1 — Use main file UI repair [file peerj-cs-10-2028-s001.zip › MUI Repair code and Data/component Occlusion/10.jpg]

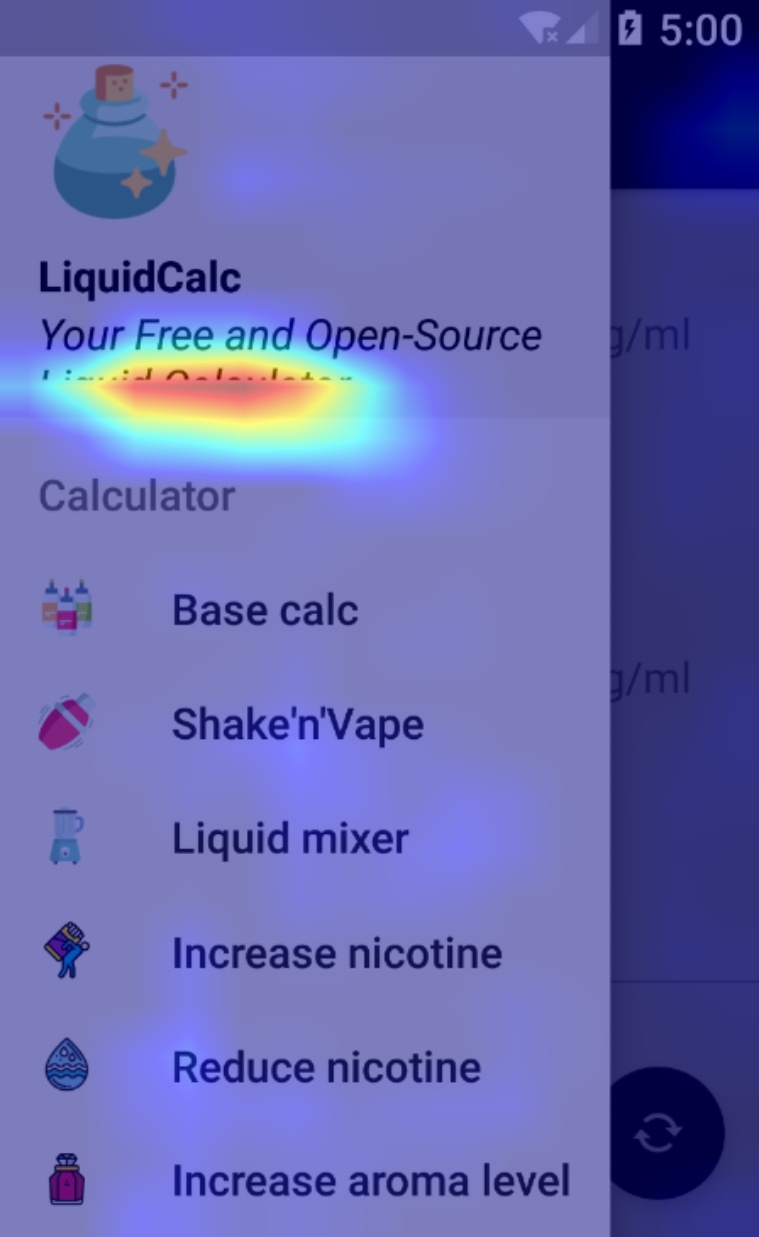

Supplement: Supplemental Information 1 — Use main file UI repair [file peerj-cs-10-2028-s001.zip › MUI Repair code and Data/component Occlusion/10cam.jpg]

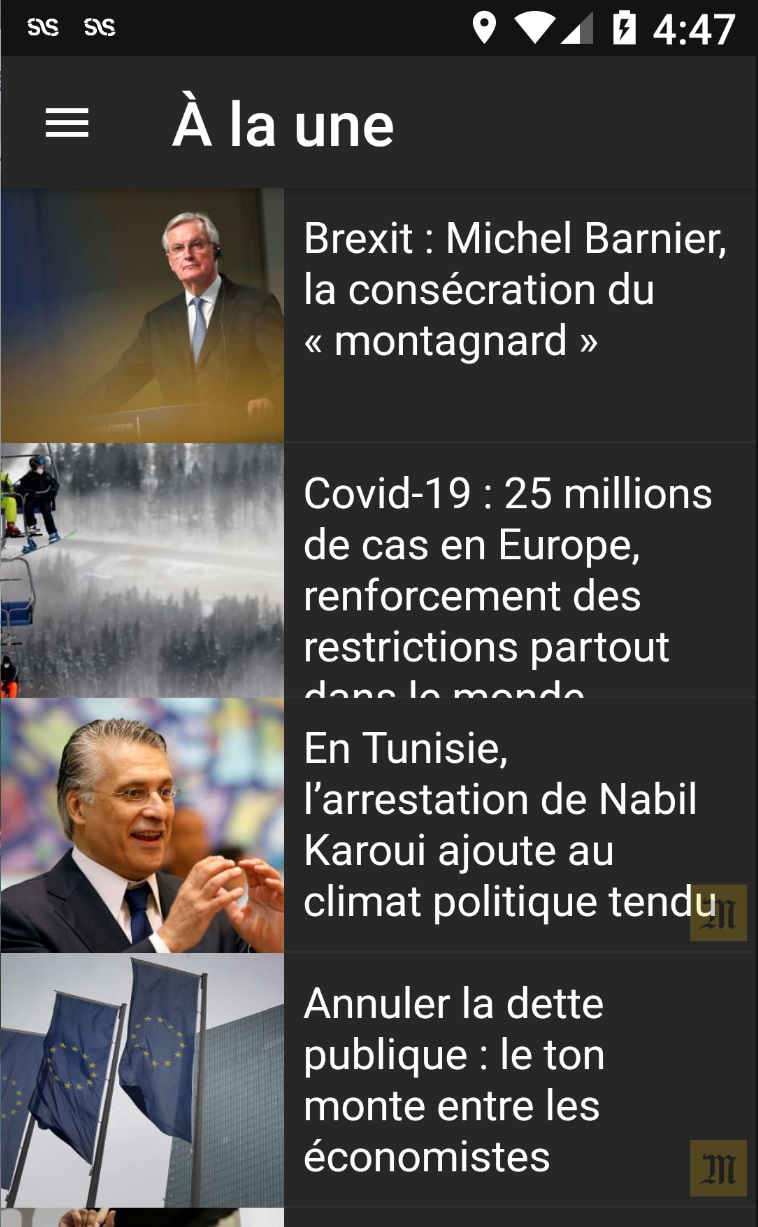

Supplement: Supplemental Information 1 — Use main file UI repair [file peerj-cs-10-2028-s001.zip › MUI Repair code and Data/component Occlusion/11.jpg]

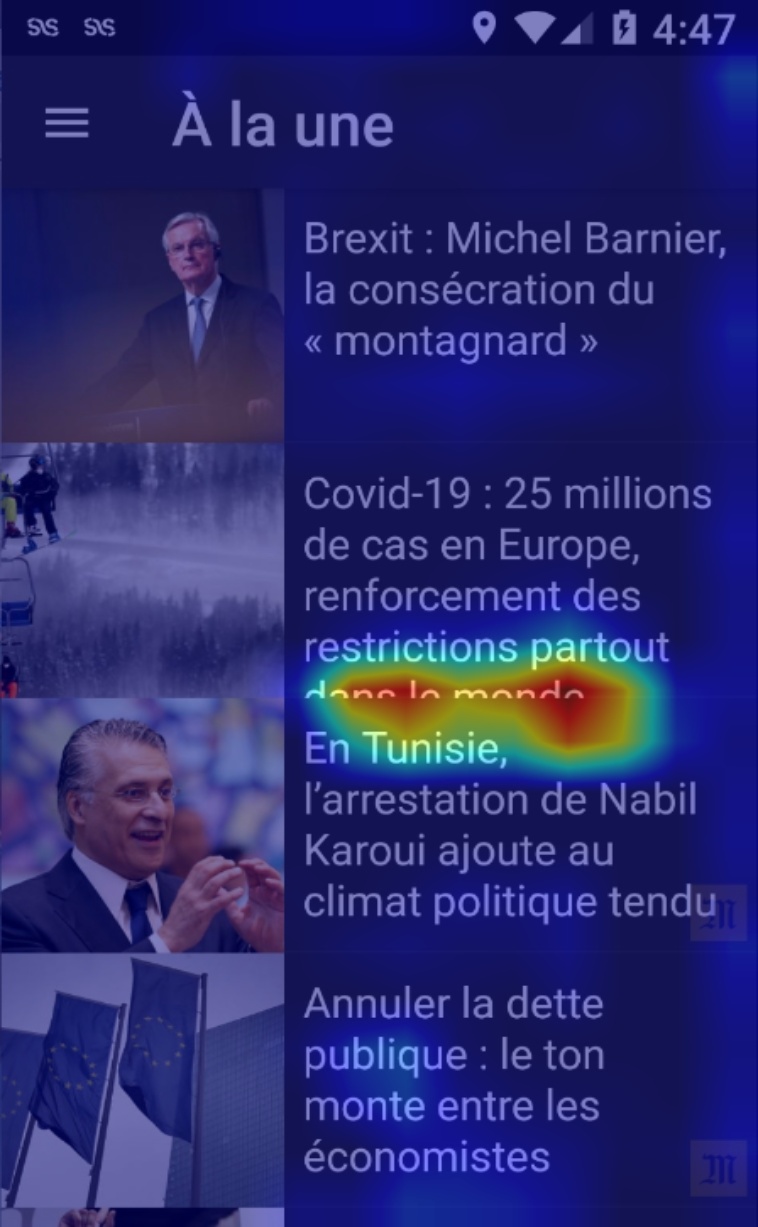

Supplement: Supplemental Information 1 — Use main file UI repair [file peerj-cs-10-2028-s001.zip › MUI Repair code and Data/component Occlusion/11cam.jpg]

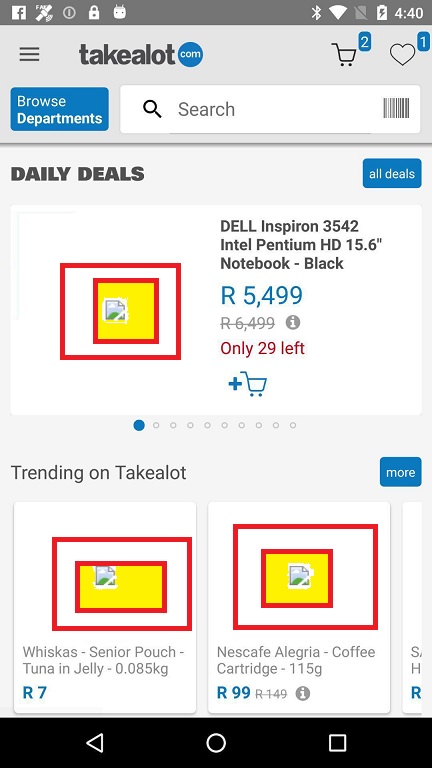

Supplement: Supplemental Information 1 — Use main file UI repair [file peerj-cs-10-2028-s001.zip › MUI Repair code and Data/component Occlusion/12.jpg]

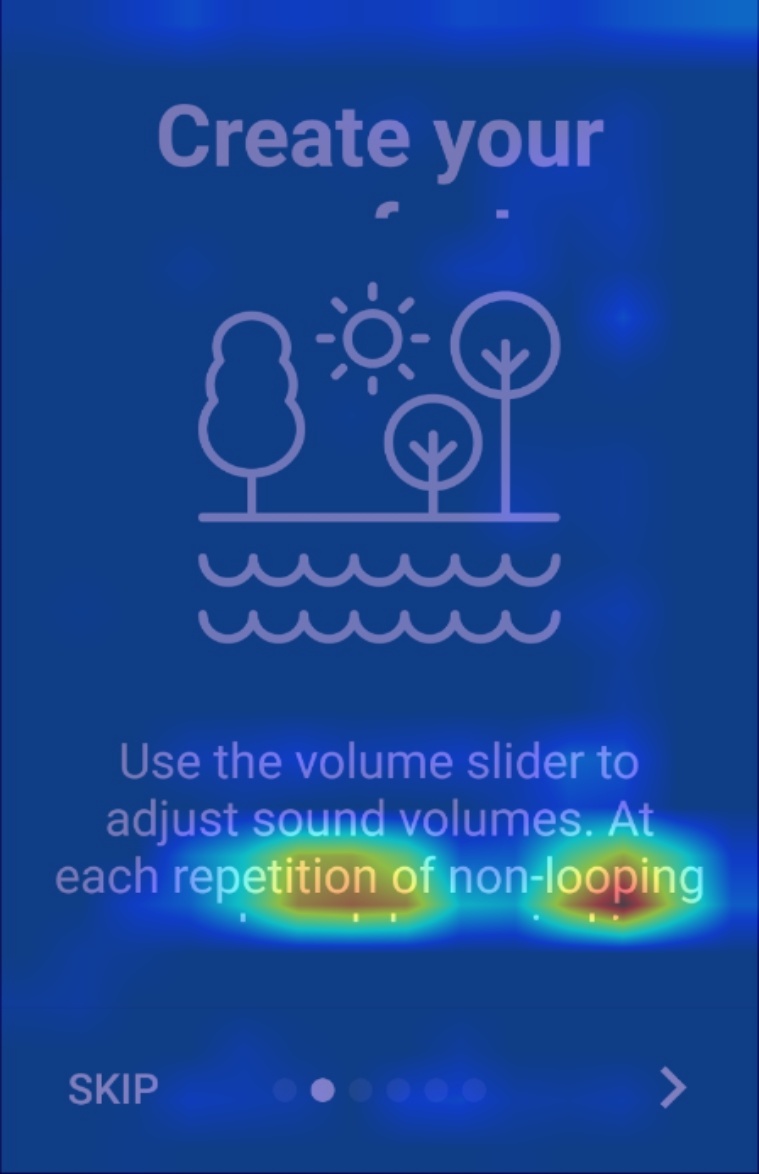

Supplement: Supplemental Information 1 — Use main file UI repair [file peerj-cs-10-2028-s001.zip › MUI Repair code and Data/component Occlusion/12cam.jpg]

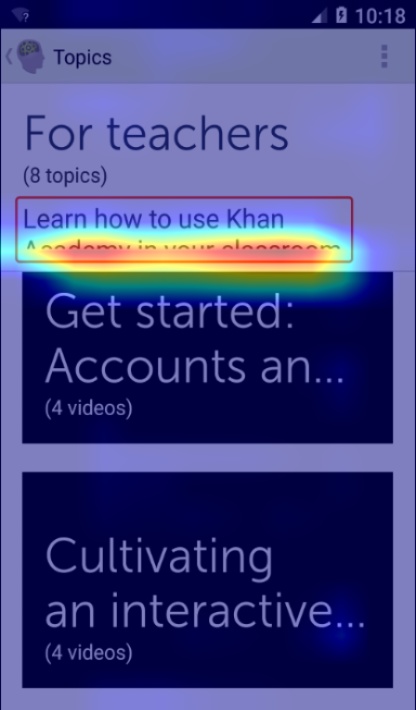

Supplement: Supplemental Information 1 — Use main file UI repair [file peerj-cs-10-2028-s001.zip › MUI Repair code and Data/component Occlusion/1cam.jpg]

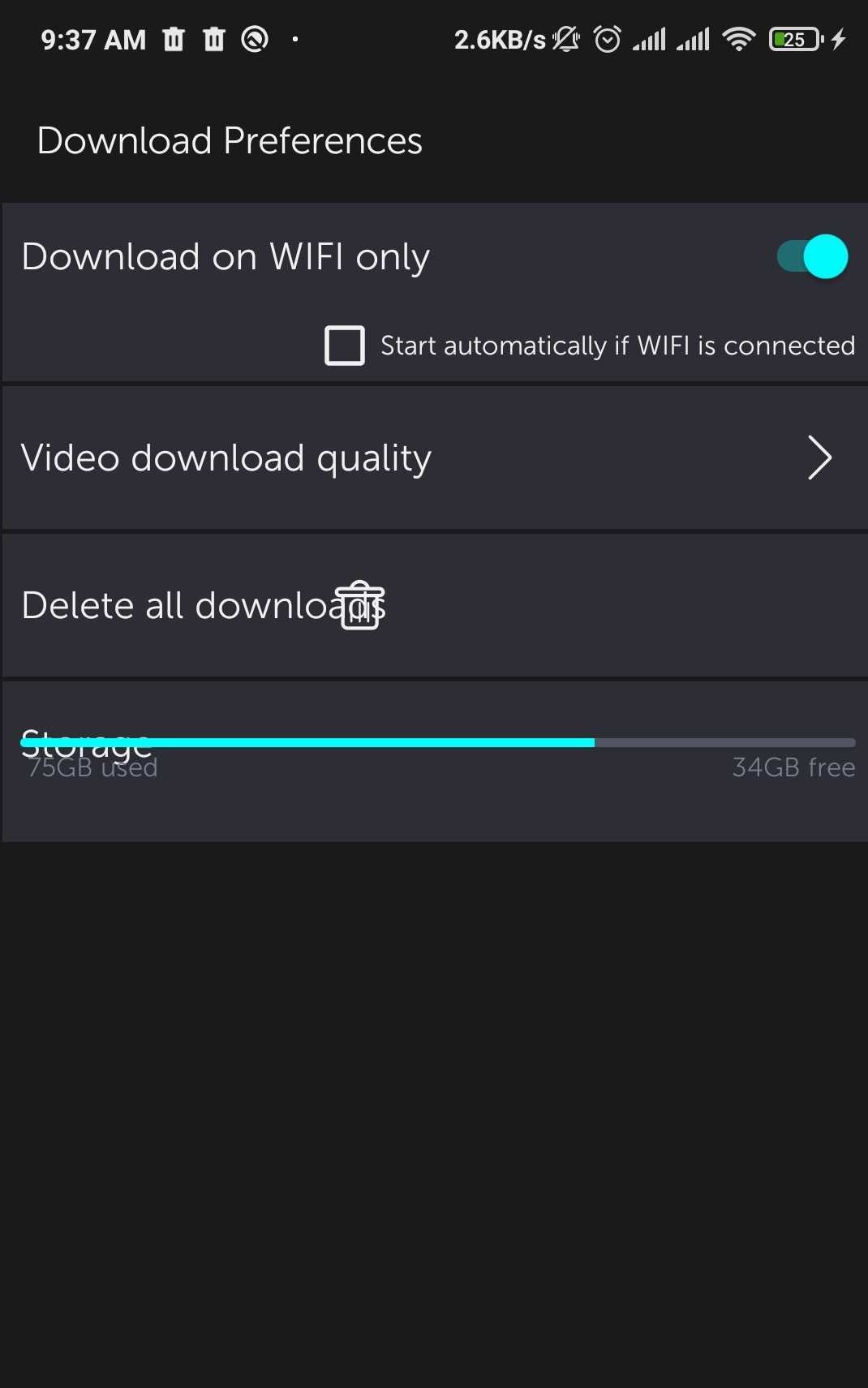

Supplement: Supplemental Information 1 — Use main file UI repair [file peerj-cs-10-2028-s001.zip › MUI Repair code and Data/component Occlusion/2.jpg]

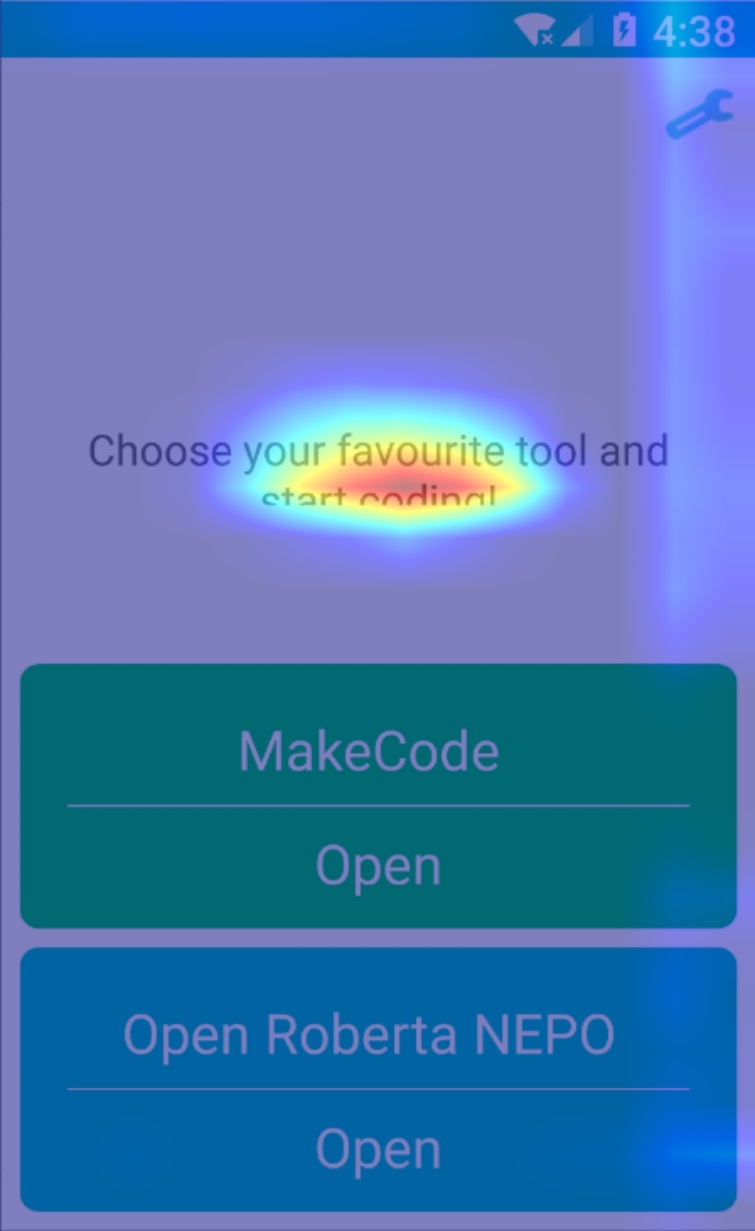

Supplement: Supplemental Information 1 — Use main file UI repair [file peerj-cs-10-2028-s001.zip › MUI Repair code and Data/component Occlusion/2cam.jpg]

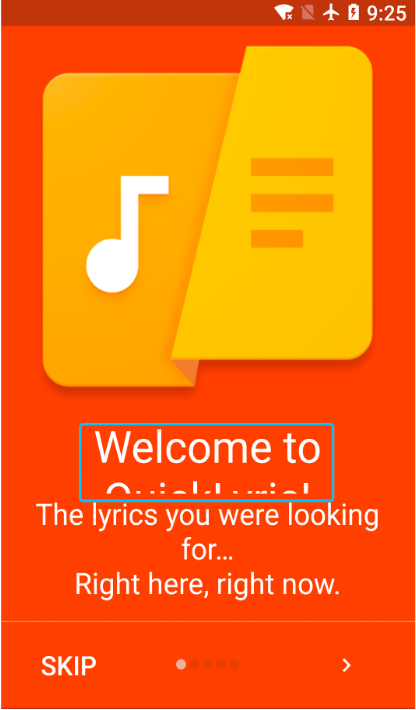

Supplement: Supplemental Information 1 — Use main file UI repair [file peerj-cs-10-2028-s001.zip › MUI Repair code and Data/component Occlusion/3.jpg]

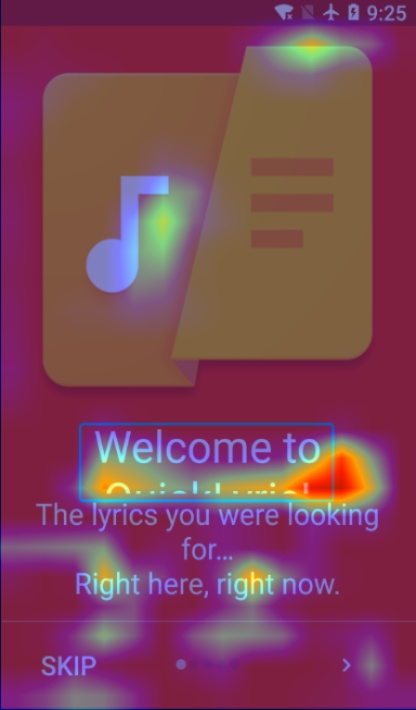

Supplement: Supplemental Information 1 — Use main file UI repair [file peerj-cs-10-2028-s001.zip › MUI Repair code and Data/component Occlusion/3cam.jpg]

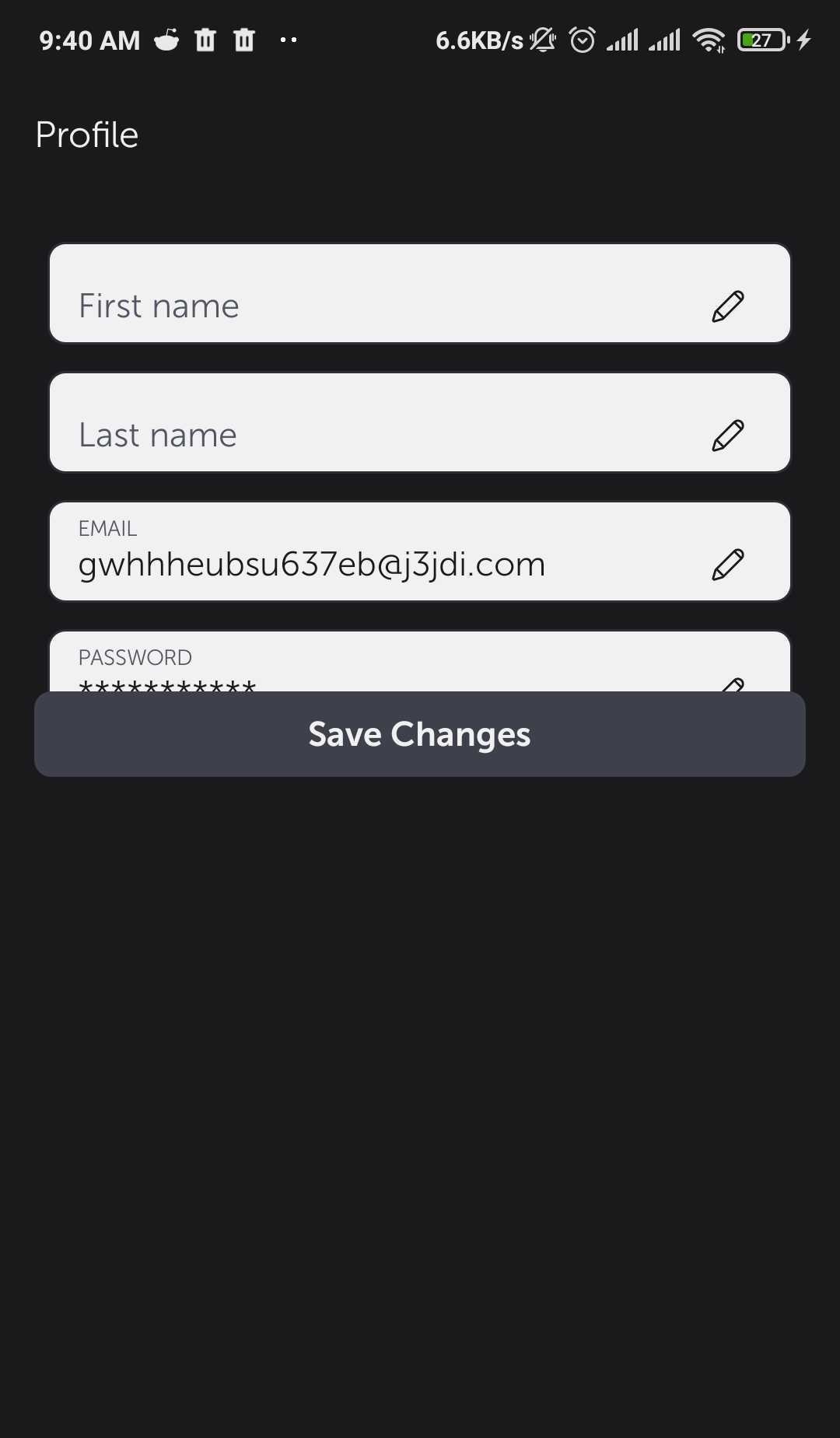

Supplement: Supplemental Information 1 — Use main file UI repair [file peerj-cs-10-2028-s001.zip › MUI Repair code and Data/component Occlusion/4.jpg]

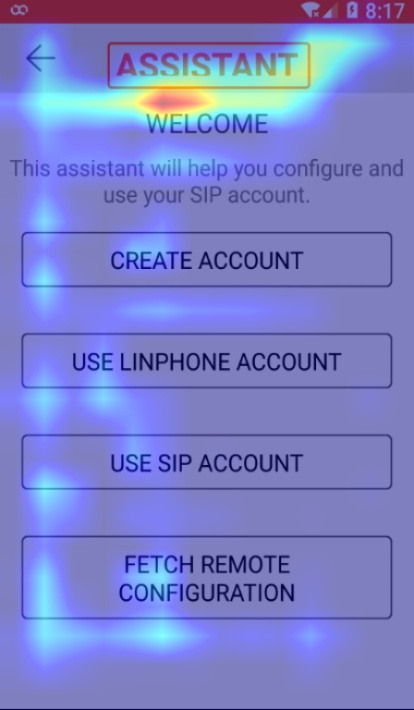

Supplement: Supplemental Information 1 — Use main file UI repair [file peerj-cs-10-2028-s001.zip › MUI Repair code and Data/component Occlusion/4cam.jpg]

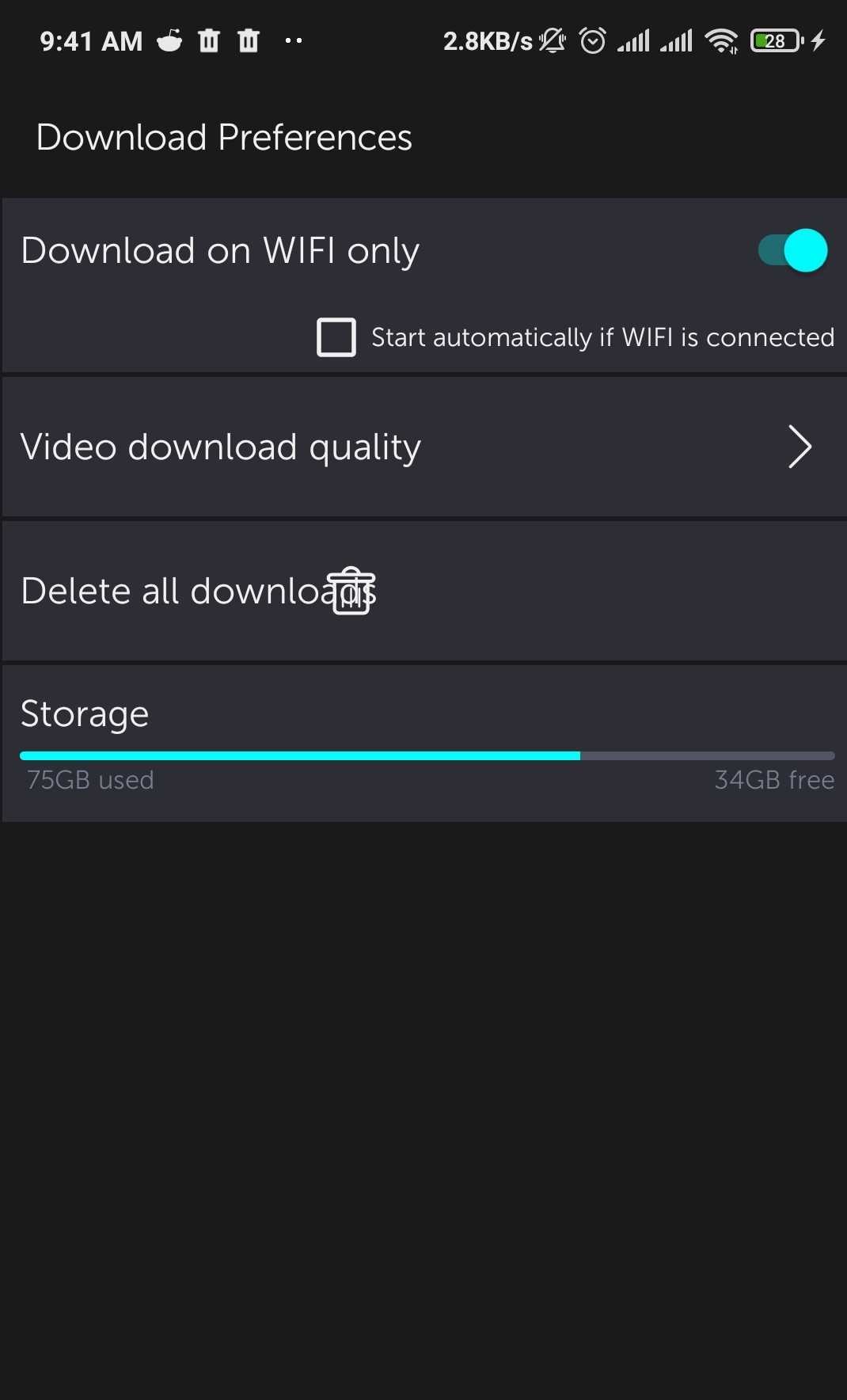

Supplement: Supplemental Information 1 — Use main file UI repair [file peerj-cs-10-2028-s001.zip › MUI Repair code and Data/component Occlusion/5.jpg]

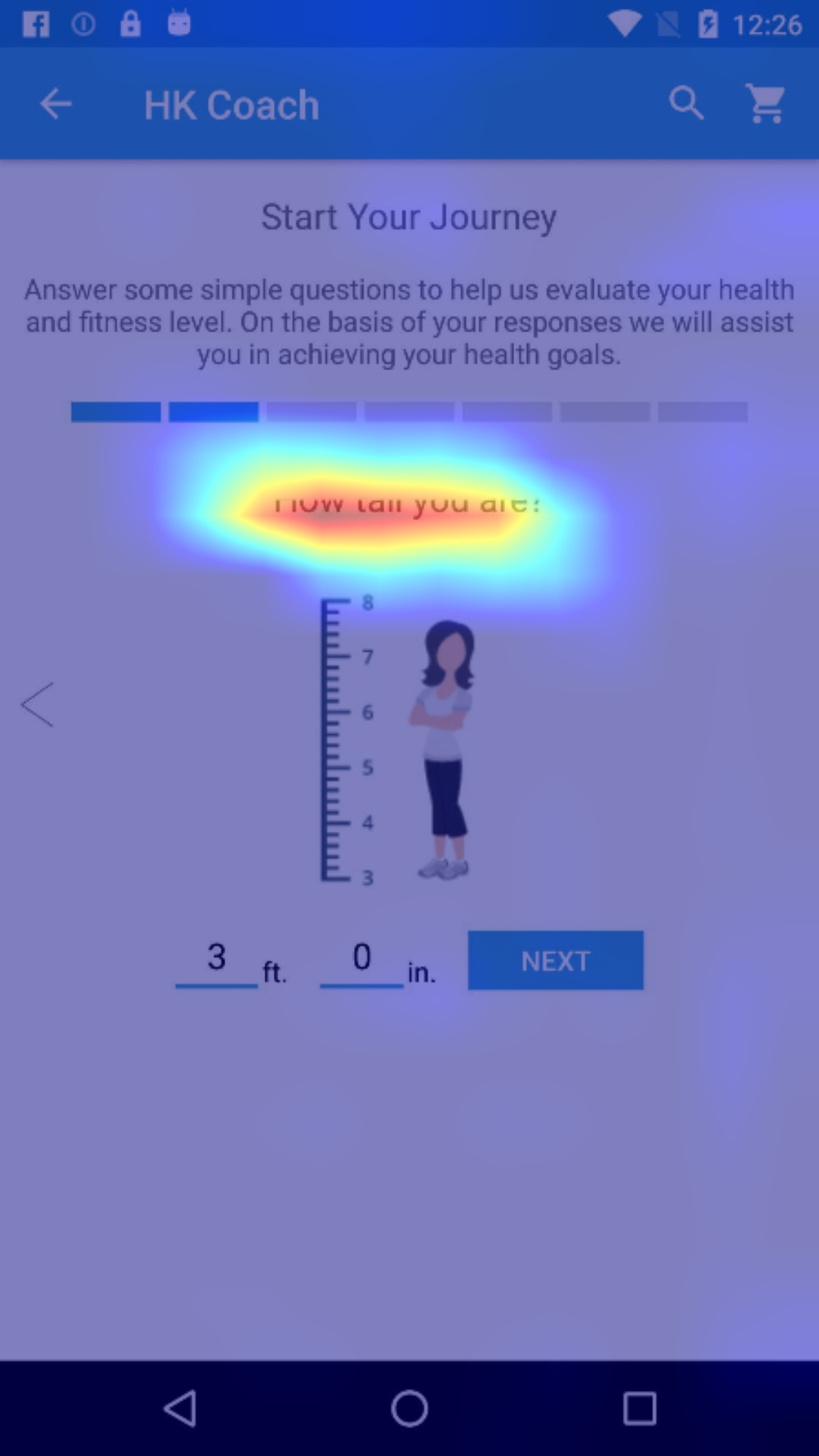

Supplement: Supplemental Information 1 — Use main file UI repair [file peerj-cs-10-2028-s001.zip › MUI Repair code and Data/component Occlusion/5cam.jpg]

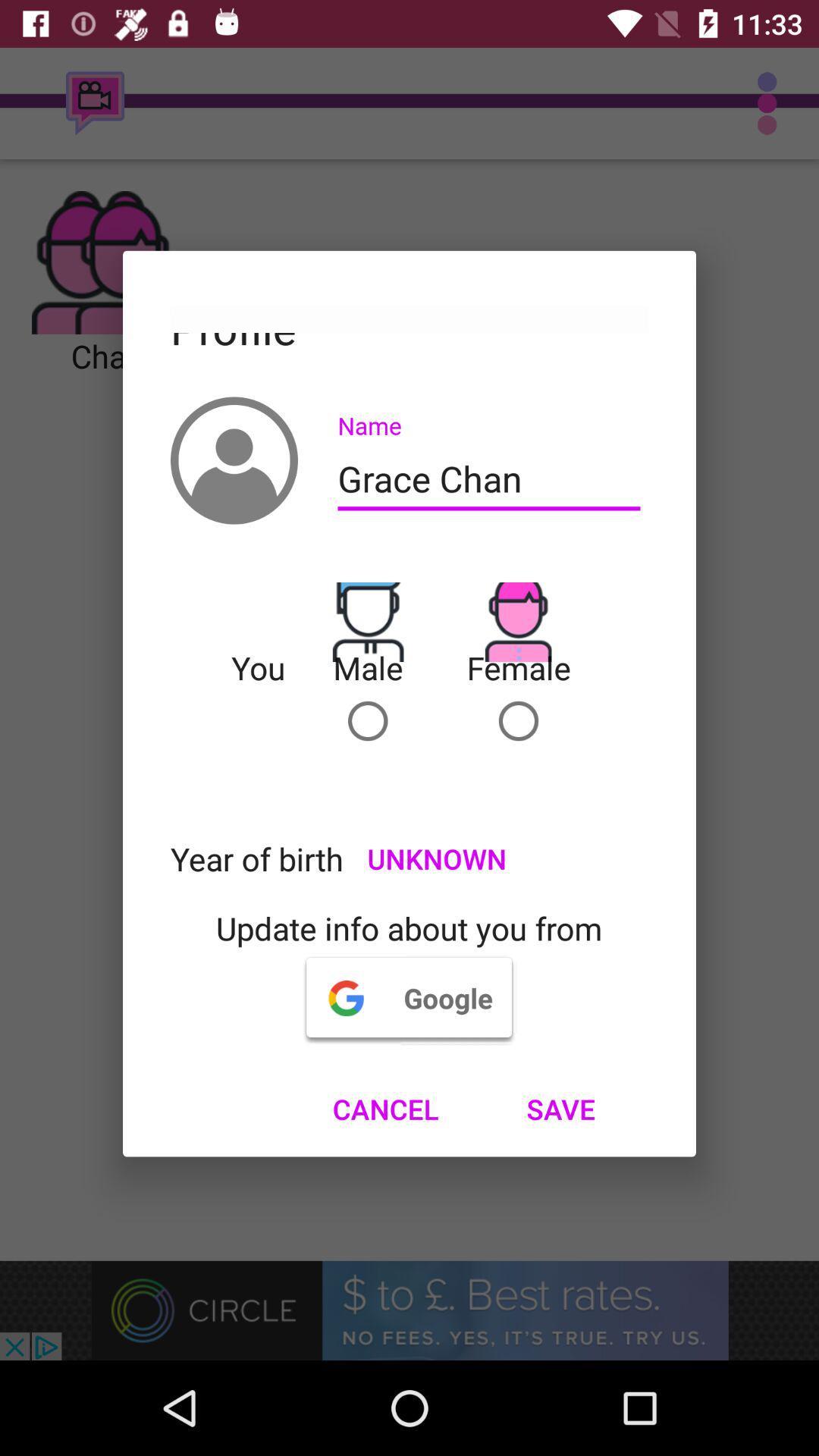

Supplement: Supplemental Information 1 — Use main file UI repair [file peerj-cs-10-2028-s001.zip › MUI Repair code and Data/component Occlusion/6.jpg]

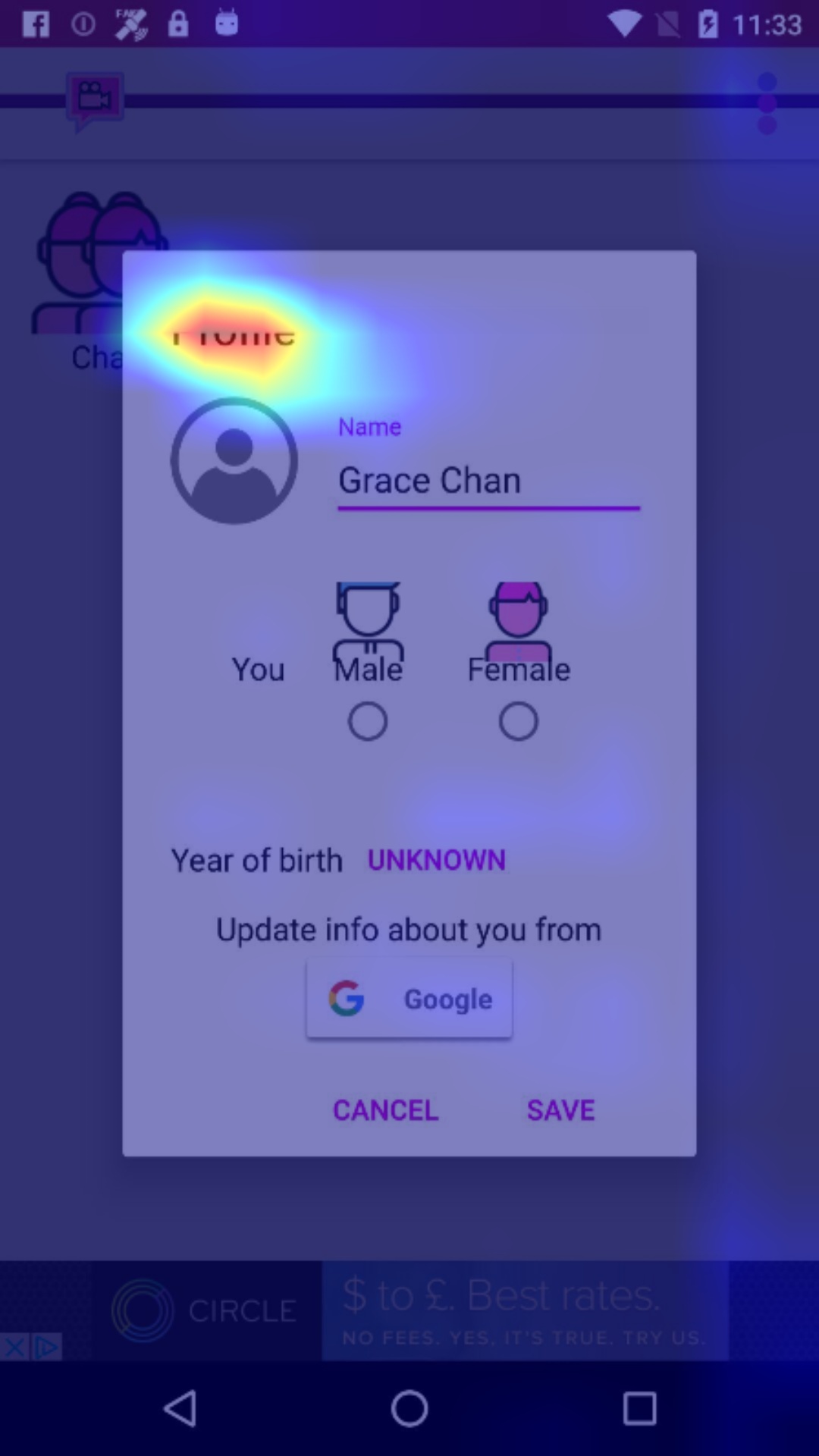

Supplement: Supplemental Information 1 — Use main file UI repair [file peerj-cs-10-2028-s001.zip › MUI Repair code and Data/component Occlusion/6cam.jpg]

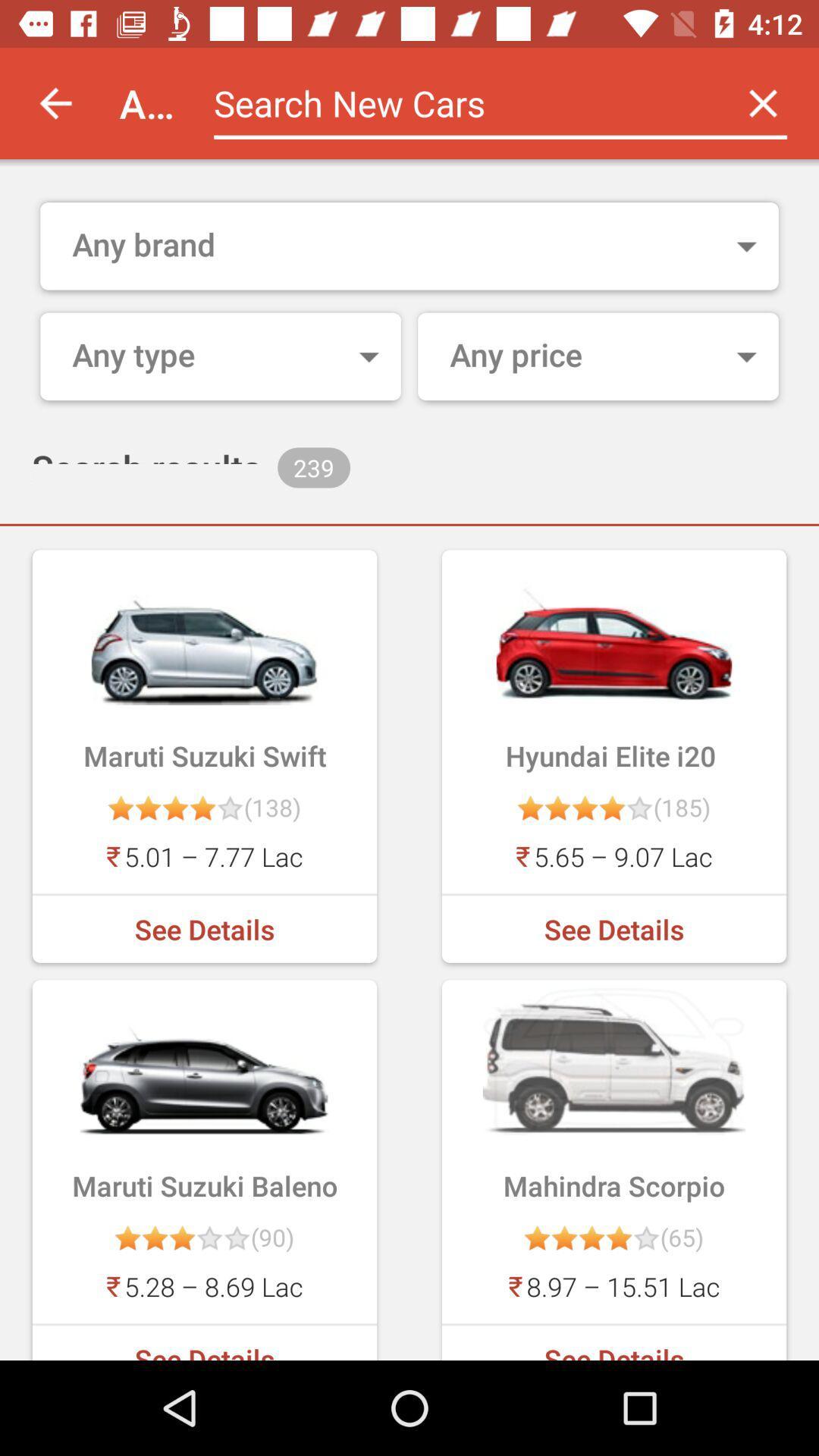

Supplement: Supplemental Information 1 — Use main file UI repair [file peerj-cs-10-2028-s001.zip › MUI Repair code and Data/component Occlusion/7.jpg]

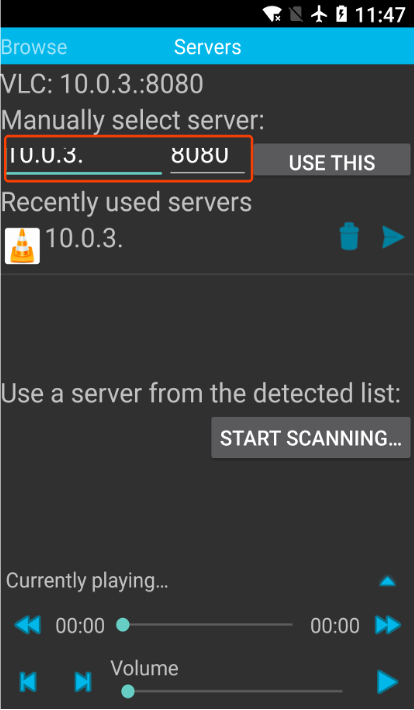

Supplement: Supplemental Information 1 — Use main file UI repair [file peerj-cs-10-2028-s001.zip › MUI Repair code and Data/component Occlusion/7.png]

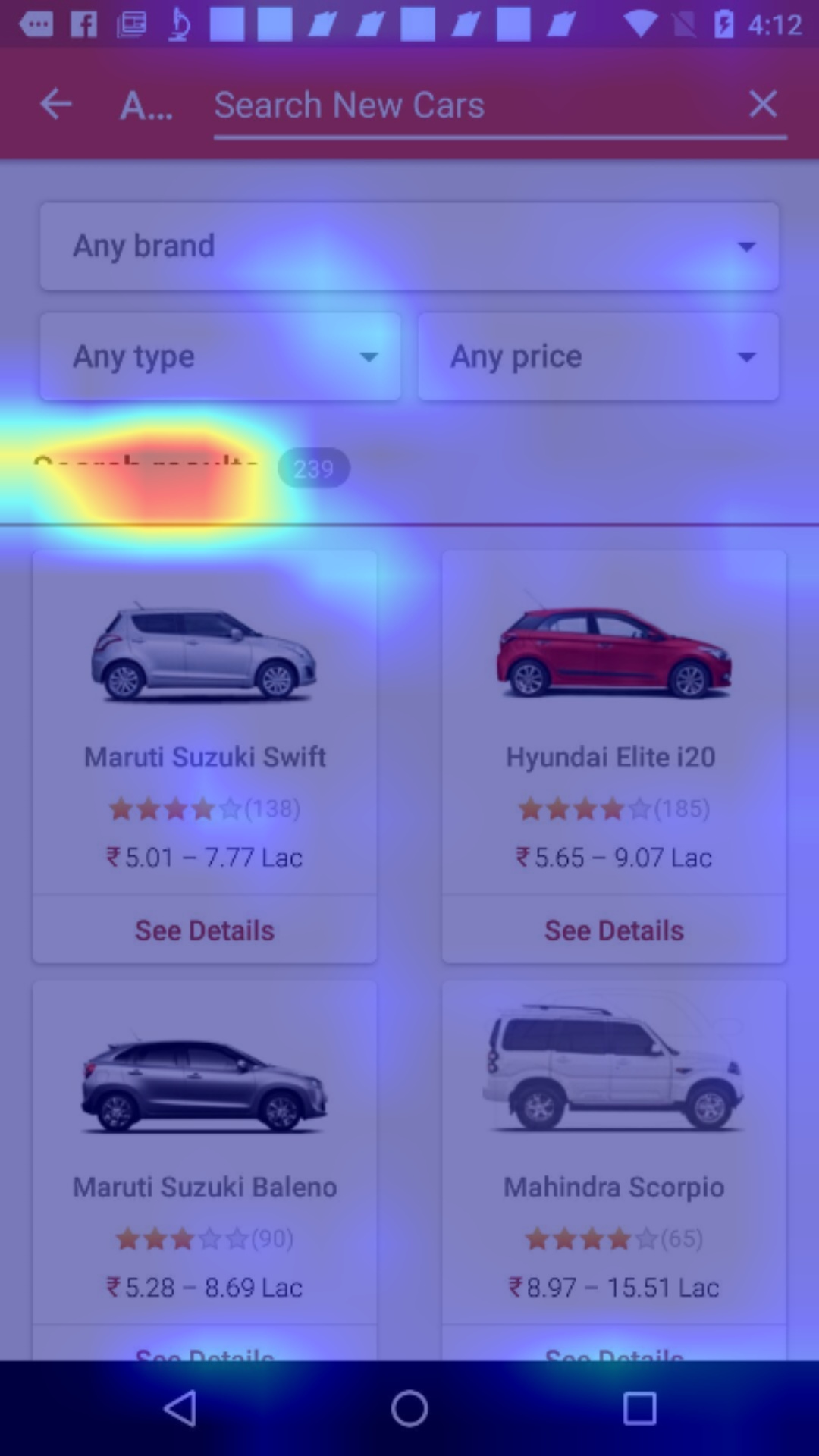

Supplement: Supplemental Information 1 — Use main file UI repair [file peerj-cs-10-2028-s001.zip › MUI Repair code and Data/component Occlusion/7cam.jpg]

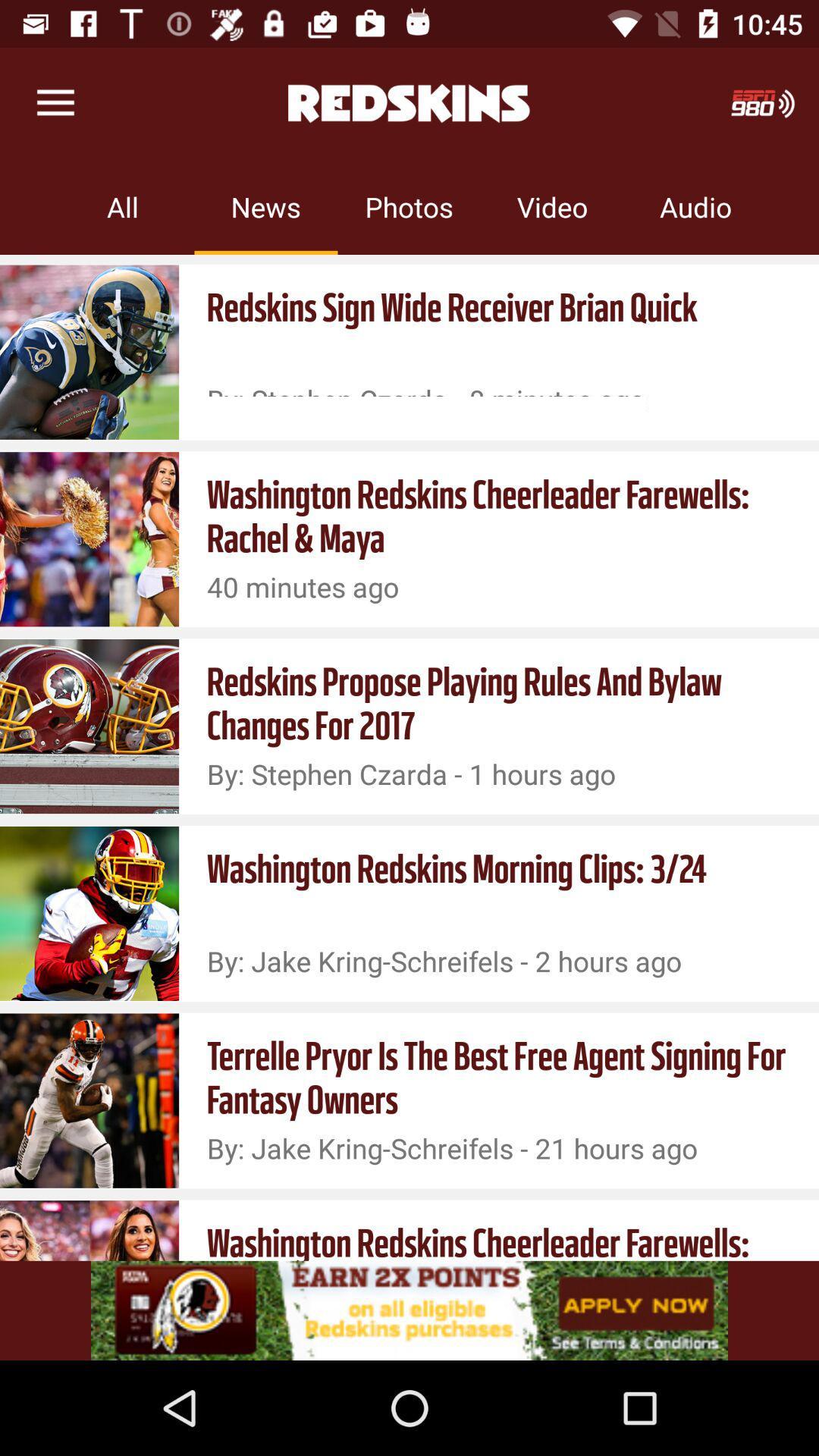

Supplement: Supplemental Information 1 — Use main file UI repair [file peerj-cs-10-2028-s001.zip › MUI Repair code and Data/component Occlusion/8.jpg]

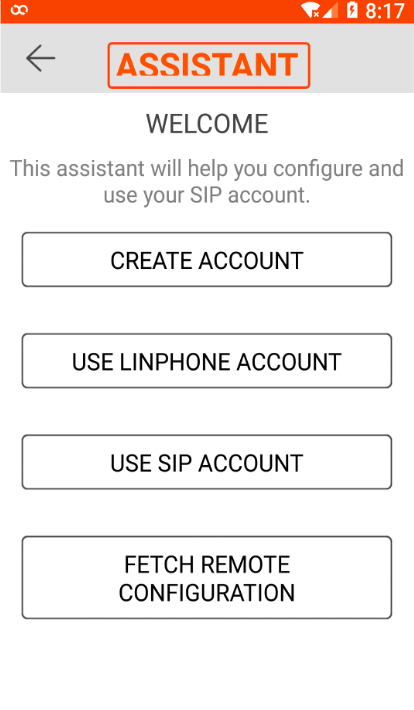

Supplement: Supplemental Information 1 — Use main file UI repair [file peerj-cs-10-2028-s001.zip › MUI Repair code and Data/component Occlusion/8.png]

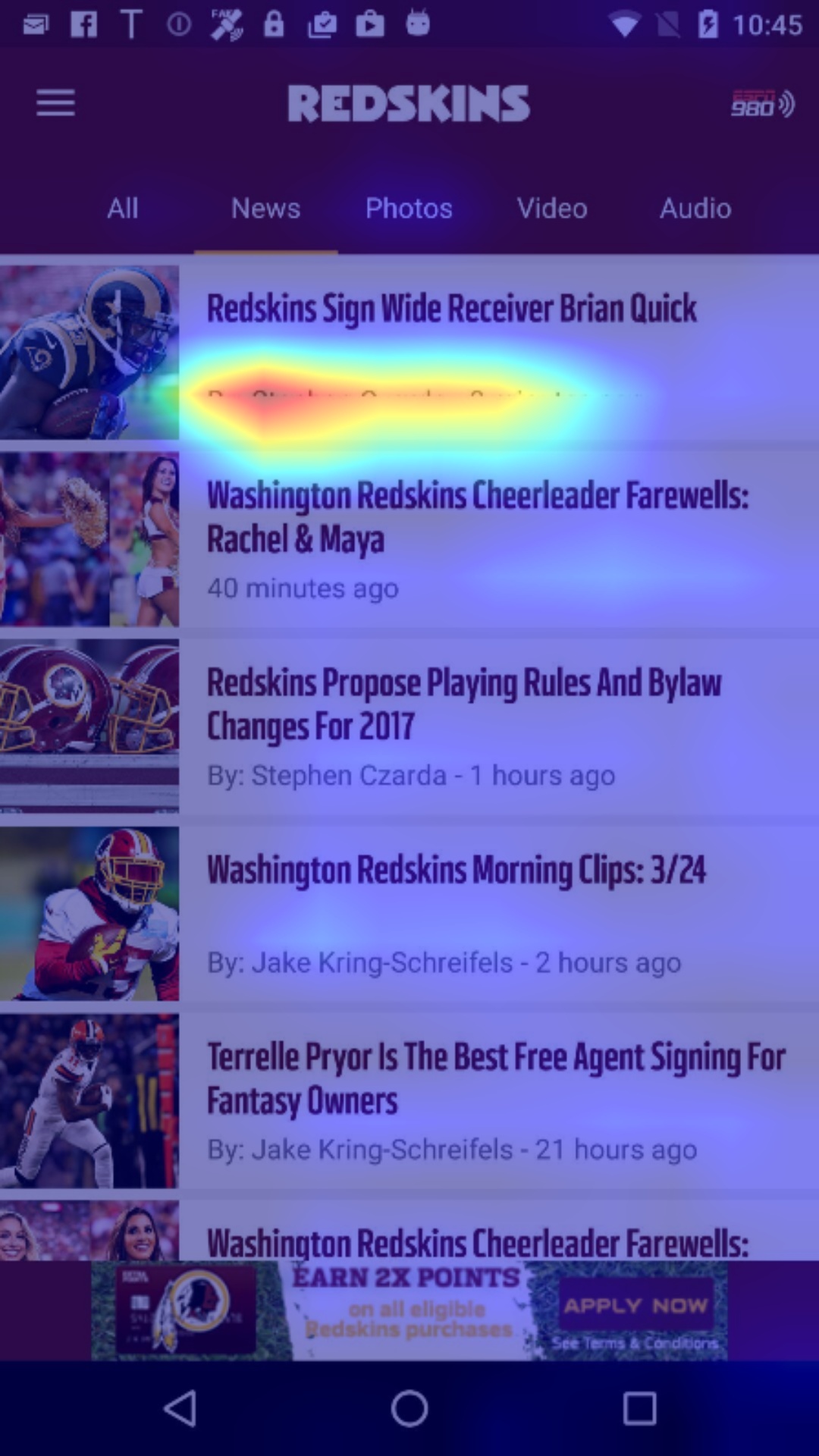

Supplement: Supplemental Information 1 — Use main file UI repair [file peerj-cs-10-2028-s001.zip › MUI Repair code and Data/component Occlusion/8cam.jpg]

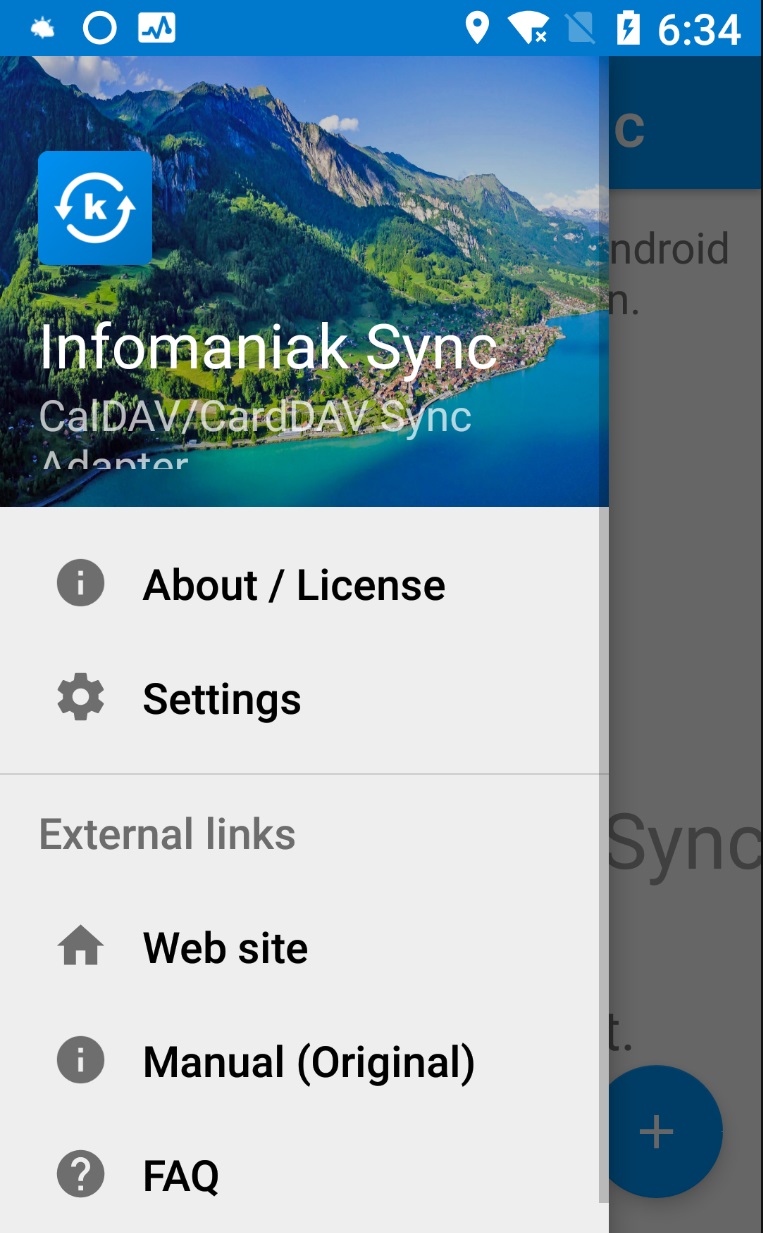

Supplement: Supplemental Information 1 — Use main file UI repair [file peerj-cs-10-2028-s001.zip › MUI Repair code and Data/component Occlusion/9.jpg]

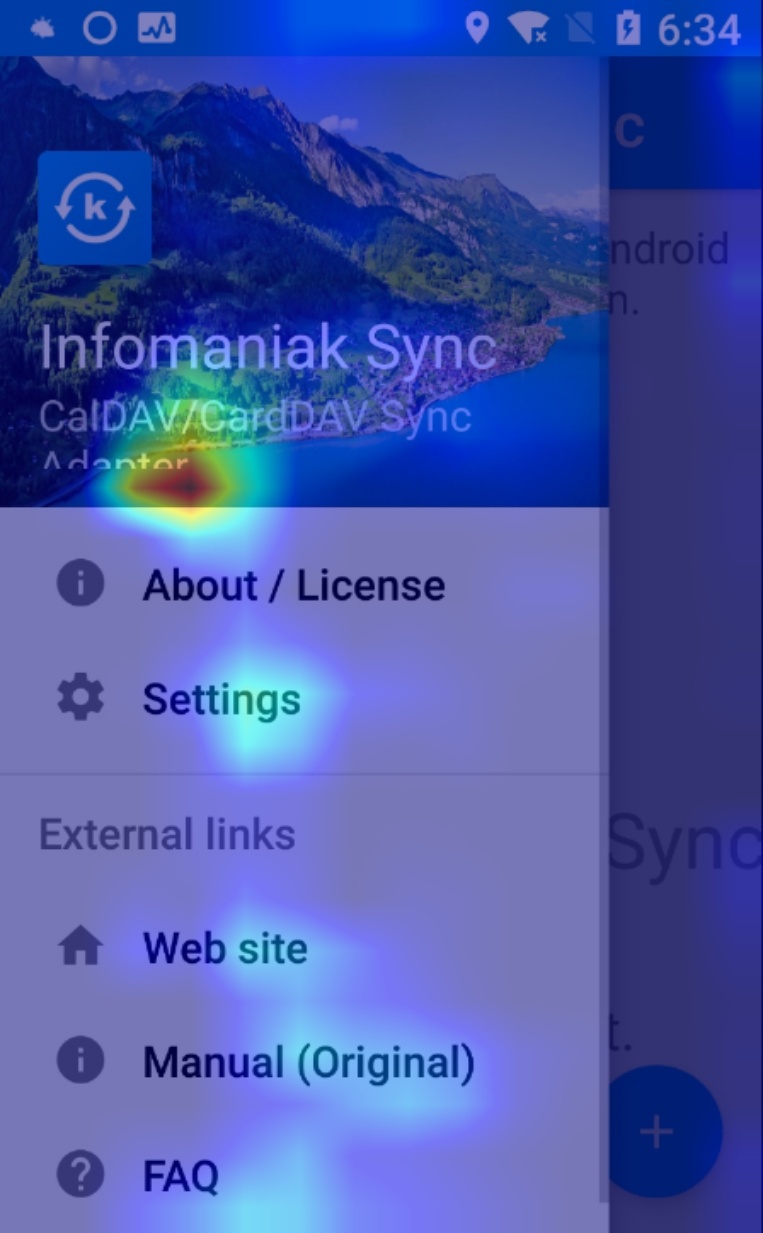

Supplement: Supplemental Information 1 — Use main file UI repair [file peerj-cs-10-2028-s001.zip › MUI Repair code and Data/component Occlusion/9cam.jpg]

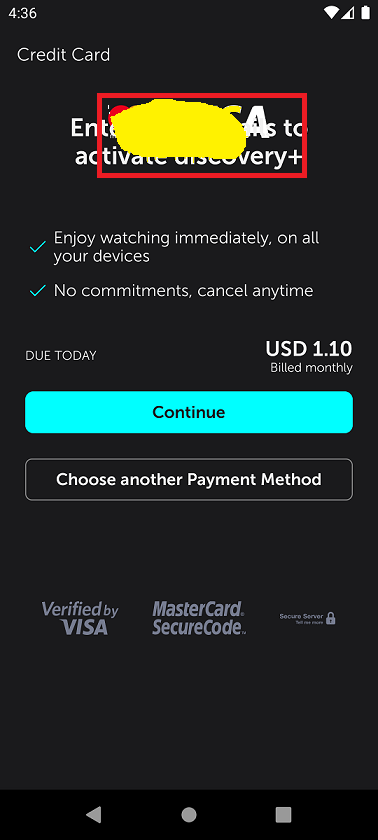

Supplement: Supplemental Information 1 — Use main file UI repair [file peerj-cs-10-2028-s001.zip › MUI Repair code and Data/component Occlusion/Approach_1 _redbox.png]

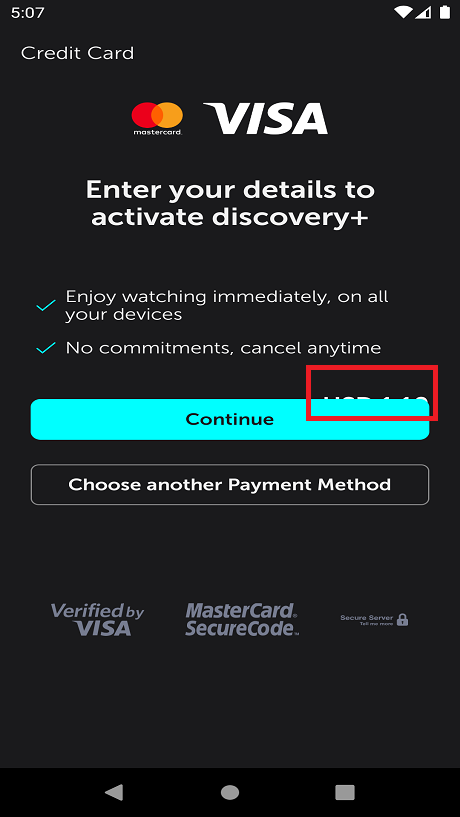

Supplement: Supplemental Information 1 — Use main file UI repair [file peerj-cs-10-2028-s001.zip › MUI Repair code and Data/component Occlusion/ND_1.png]

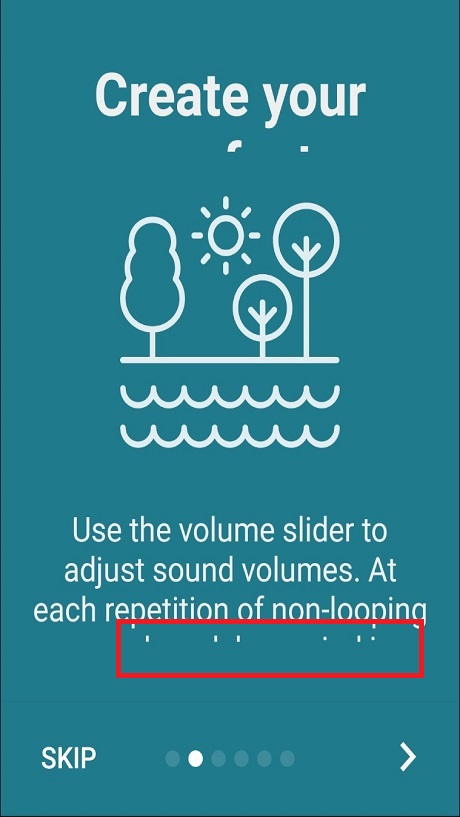

Supplement: Supplemental Information 1 — Use main file UI repair [file peerj-cs-10-2028-s001.zip › MUI Repair code and Data/component Occlusion/ND_2.jpg]

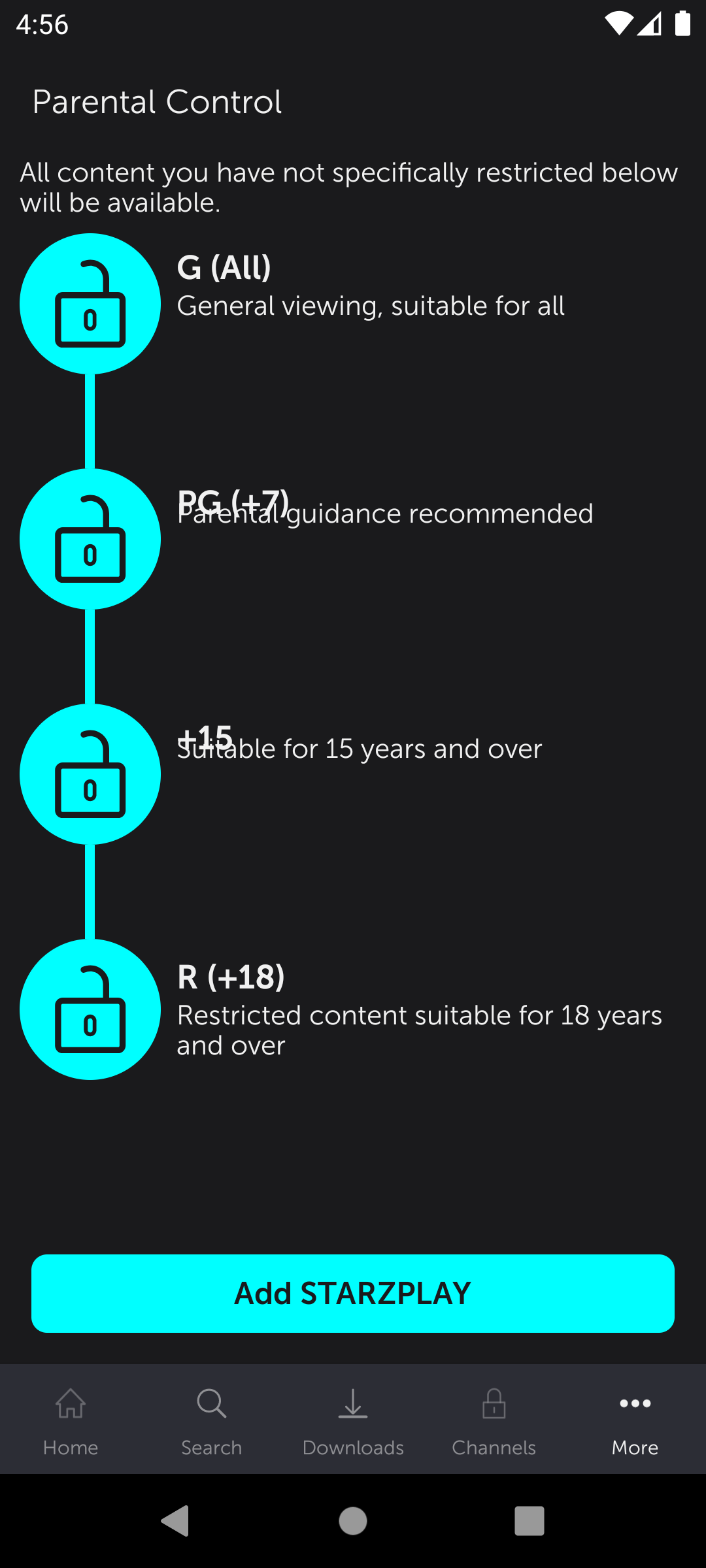

Supplement: Supplemental Information 1 — Use main file UI repair [file peerj-cs-10-2028-s001.zip › MUI Repair code and Data/component Occlusion/Screenshot_1649850984.png]

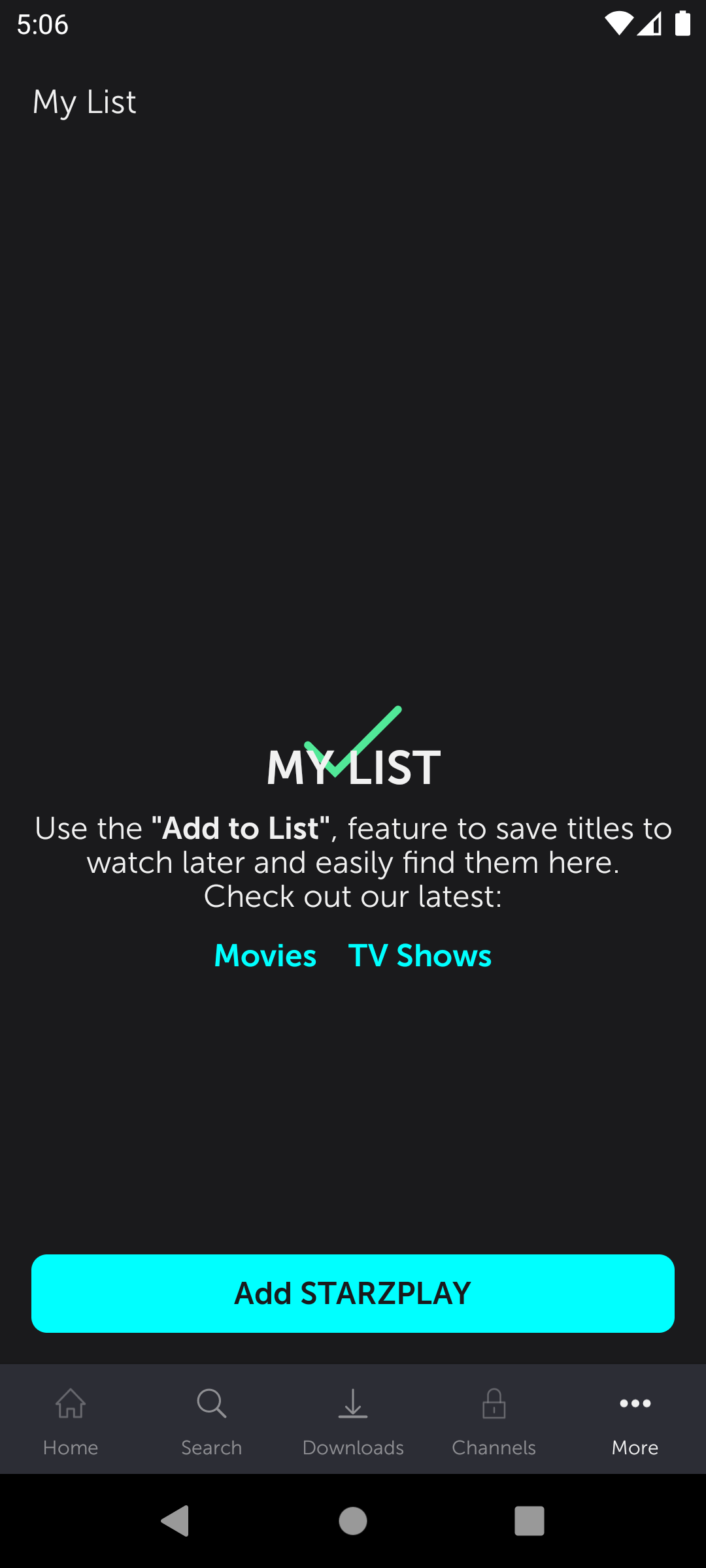

Supplement: Supplemental Information 1 — Use main file UI repair [file peerj-cs-10-2028-s001.zip › MUI Repair code and Data/component Occlusion/Screenshot_1649851612.png]

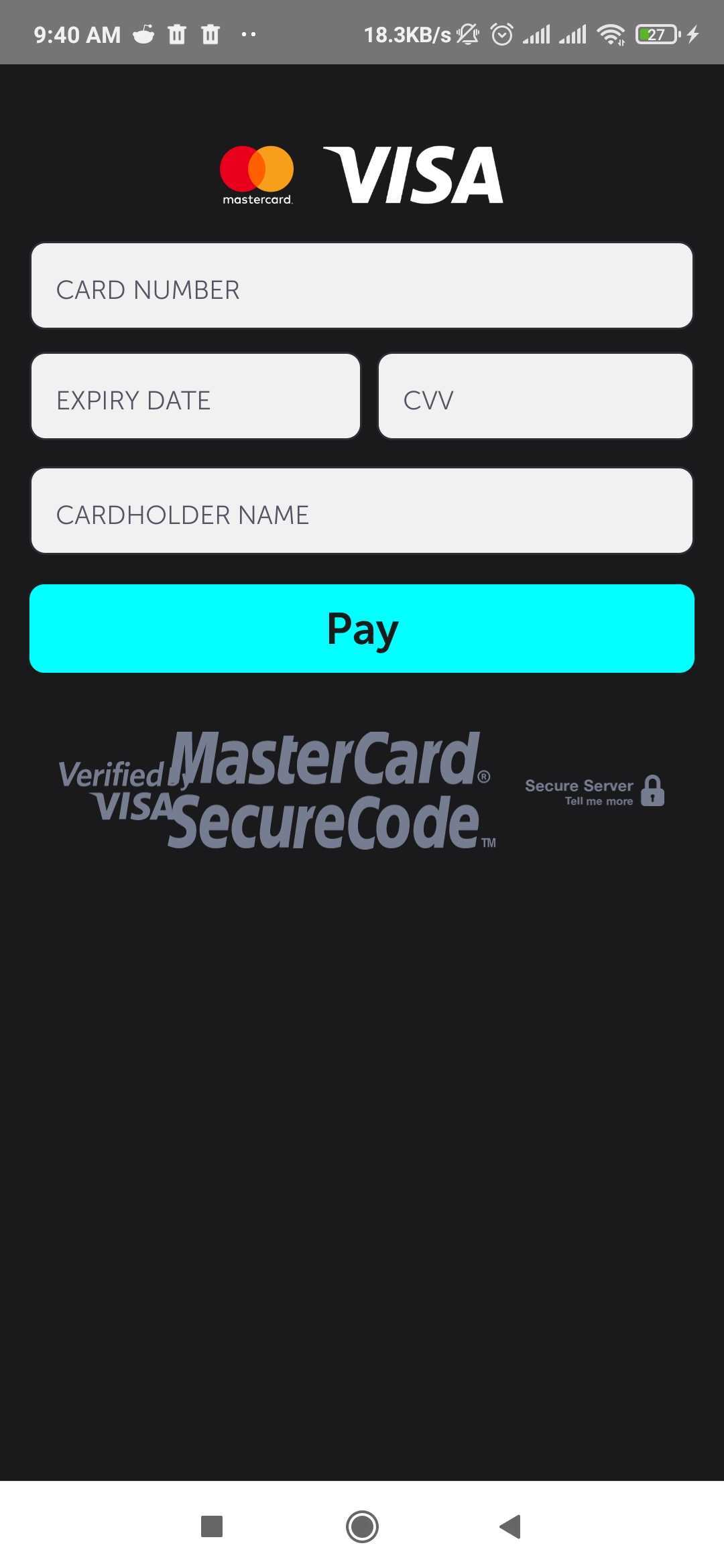

Supplement: Supplemental Information 1 — Use main file UI repair [file peerj-cs-10-2028-s001.zip › MUI Repair code and Data/component Occlusion/Screenshot_2022-04-14-09-40-46-300_com.parsifal.starz.jpg]

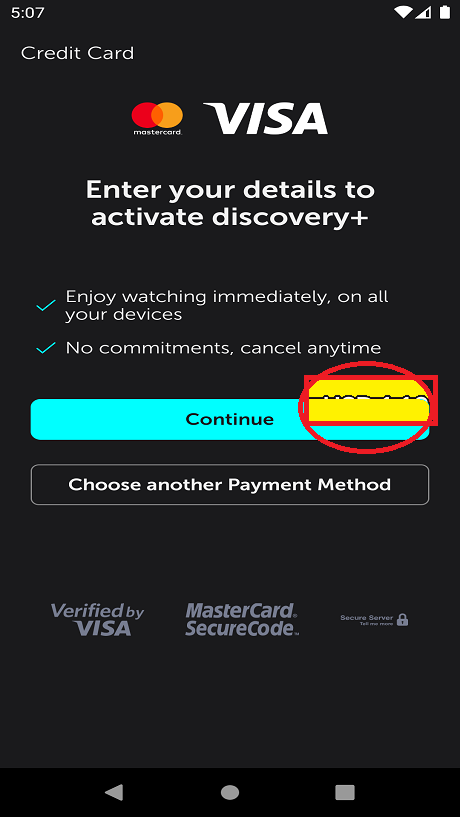

Supplement: Supplemental Information 1 — Use main file UI repair [file peerj-cs-10-2028-s001.zip › MUI Repair code and Data/component Occlusion/unable to detect.png]

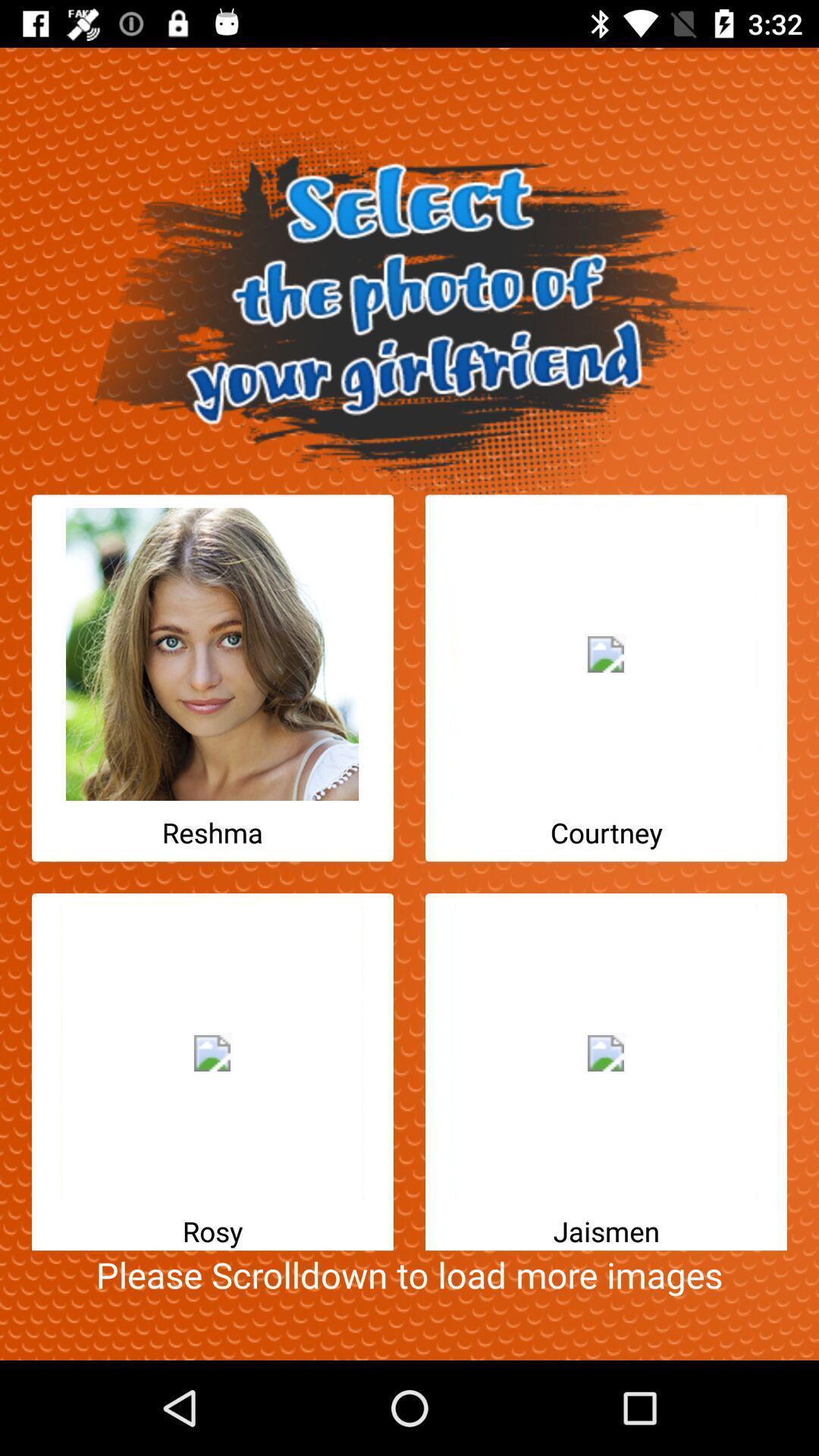

Supplement: Supplemental Information 1 — Use main file UI repair [file peerj-cs-10-2028-s001.zip › MUI Repair code and Data/missing image/1.jpg]

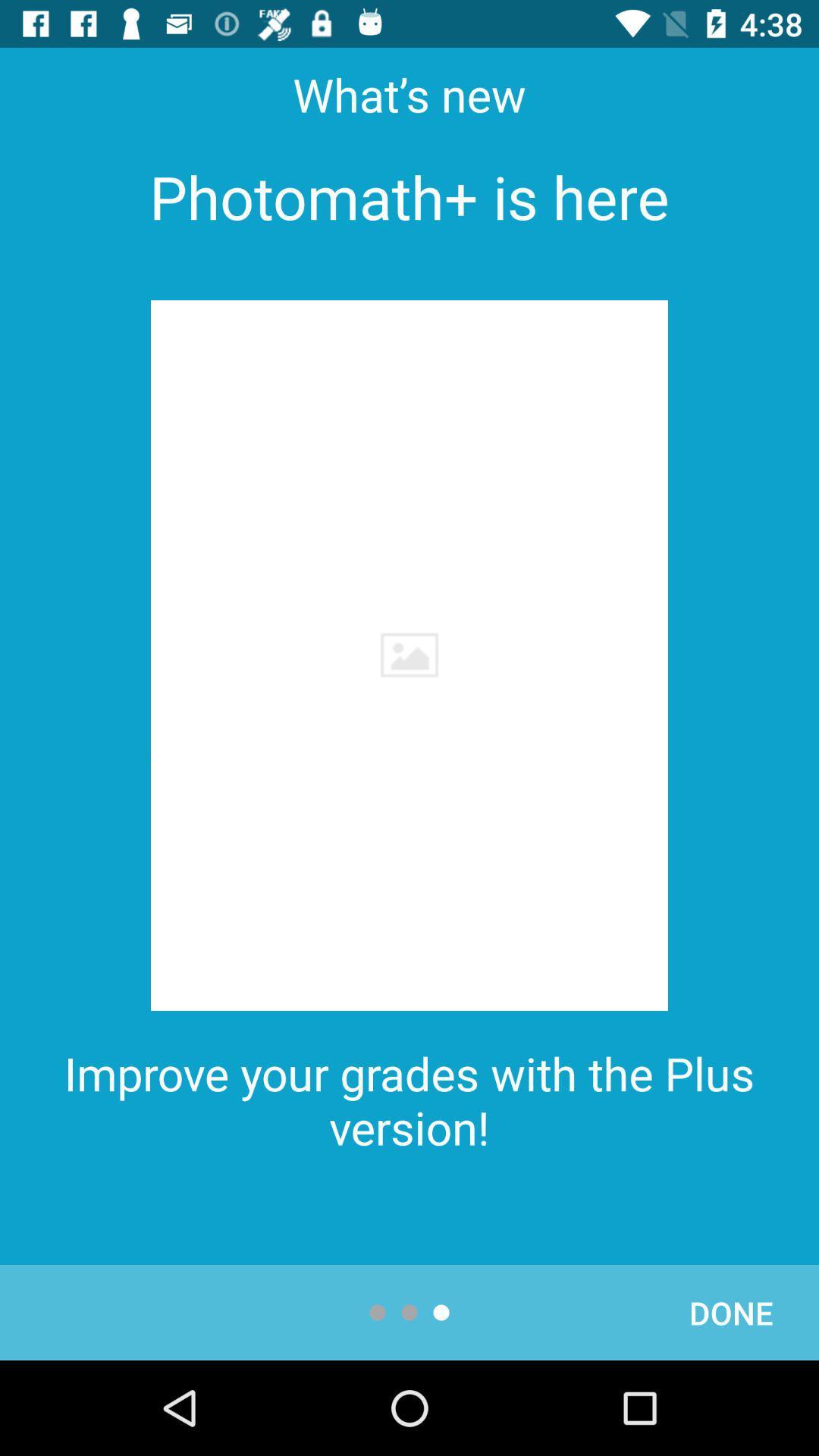

Supplement: Supplemental Information 1 — Use main file UI repair [file peerj-cs-10-2028-s001.zip › MUI Repair code and Data/missing image/10.jpg]

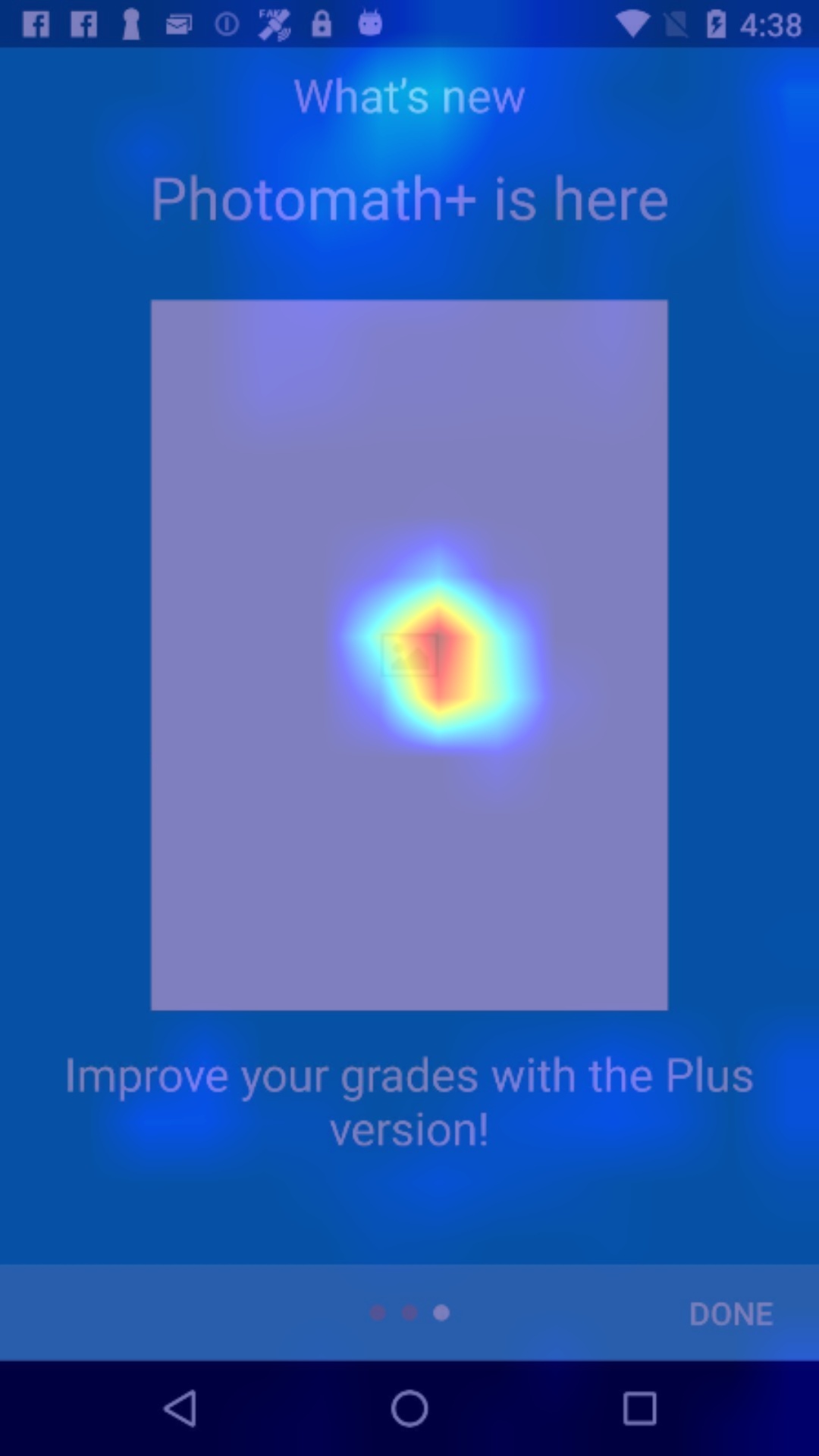

Supplement: Supplemental Information 1 — Use main file UI repair [file peerj-cs-10-2028-s001.zip › MUI Repair code and Data/missing image/10cam.jpg]

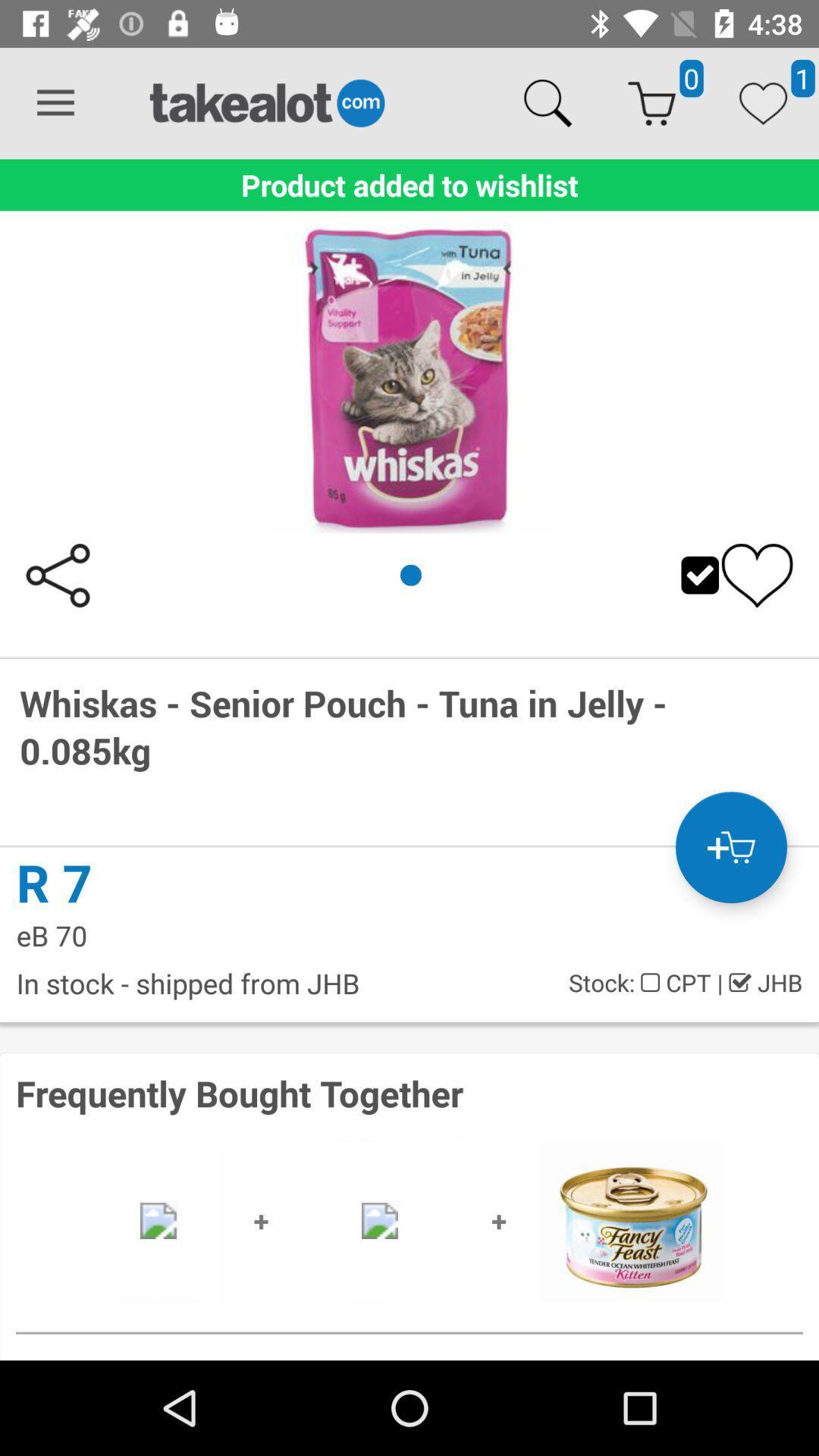

Supplement: Supplemental Information 1 — Use main file UI repair [file peerj-cs-10-2028-s001.zip › MUI Repair code and Data/missing image/11.jpg]

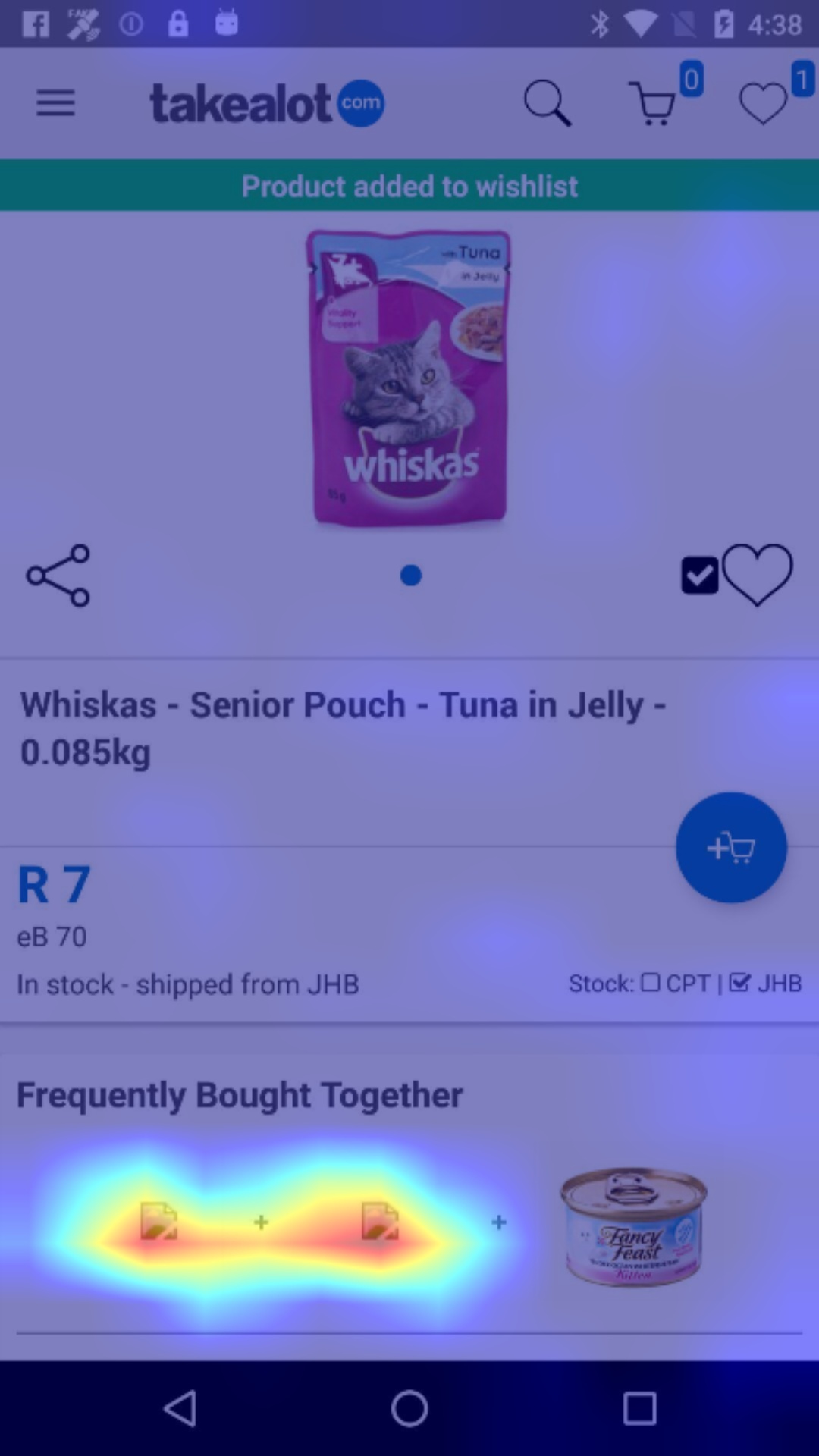

Supplement: Supplemental Information 1 — Use main file UI repair [file peerj-cs-10-2028-s001.zip › MUI Repair code and Data/missing image/11cam.jpg]

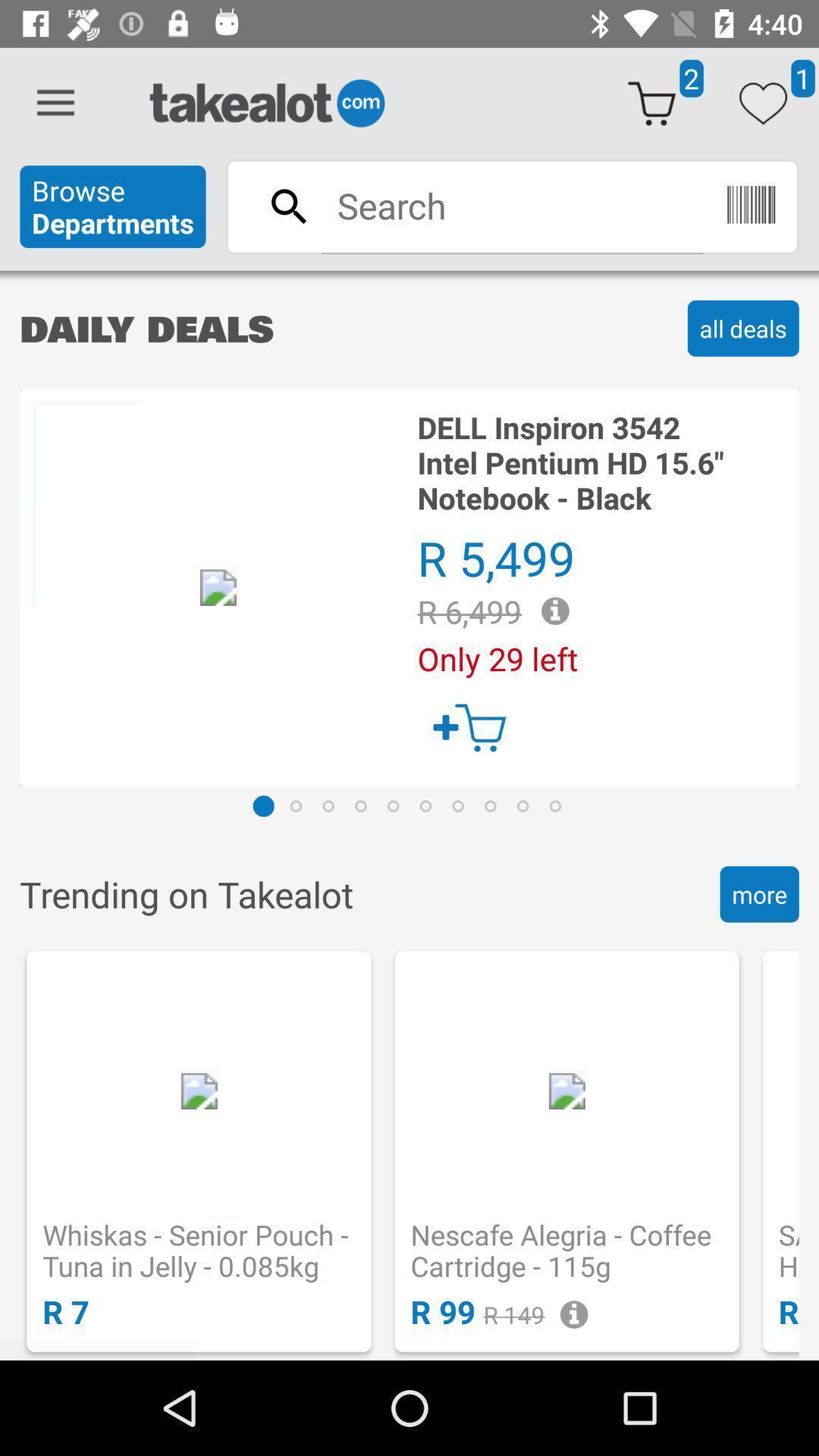

Supplement: Supplemental Information 1 — Use main file UI repair [file peerj-cs-10-2028-s001.zip › MUI Repair code and Data/missing image/12.jpg]

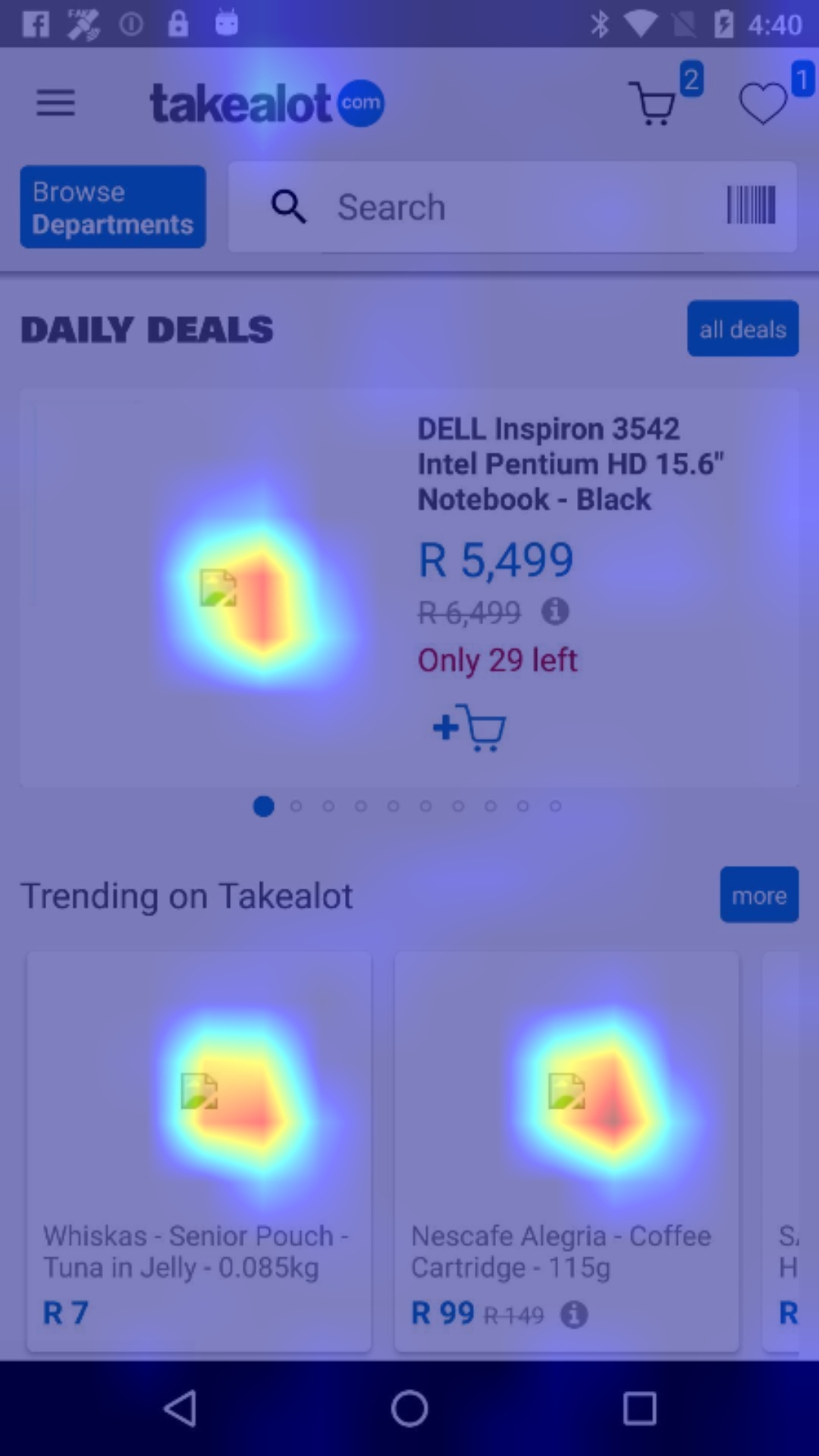

Supplement: Supplemental Information 1 — Use main file UI repair [file peerj-cs-10-2028-s001.zip › MUI Repair code and Data/missing image/12cam.jpg]

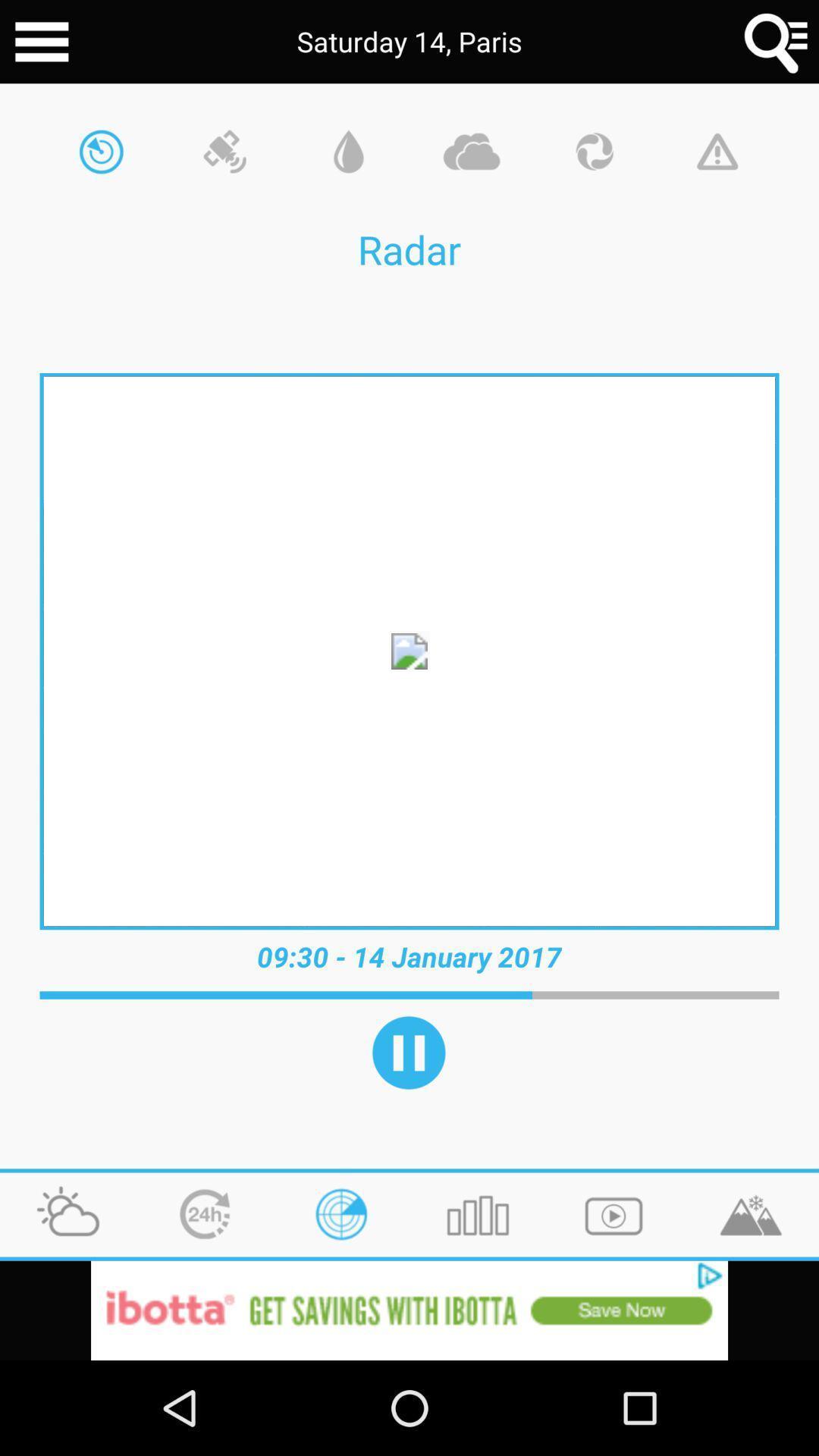

Supplement: Supplemental Information 1 — Use main file UI repair [file peerj-cs-10-2028-s001.zip › MUI Repair code and Data/missing image/13.jpg]

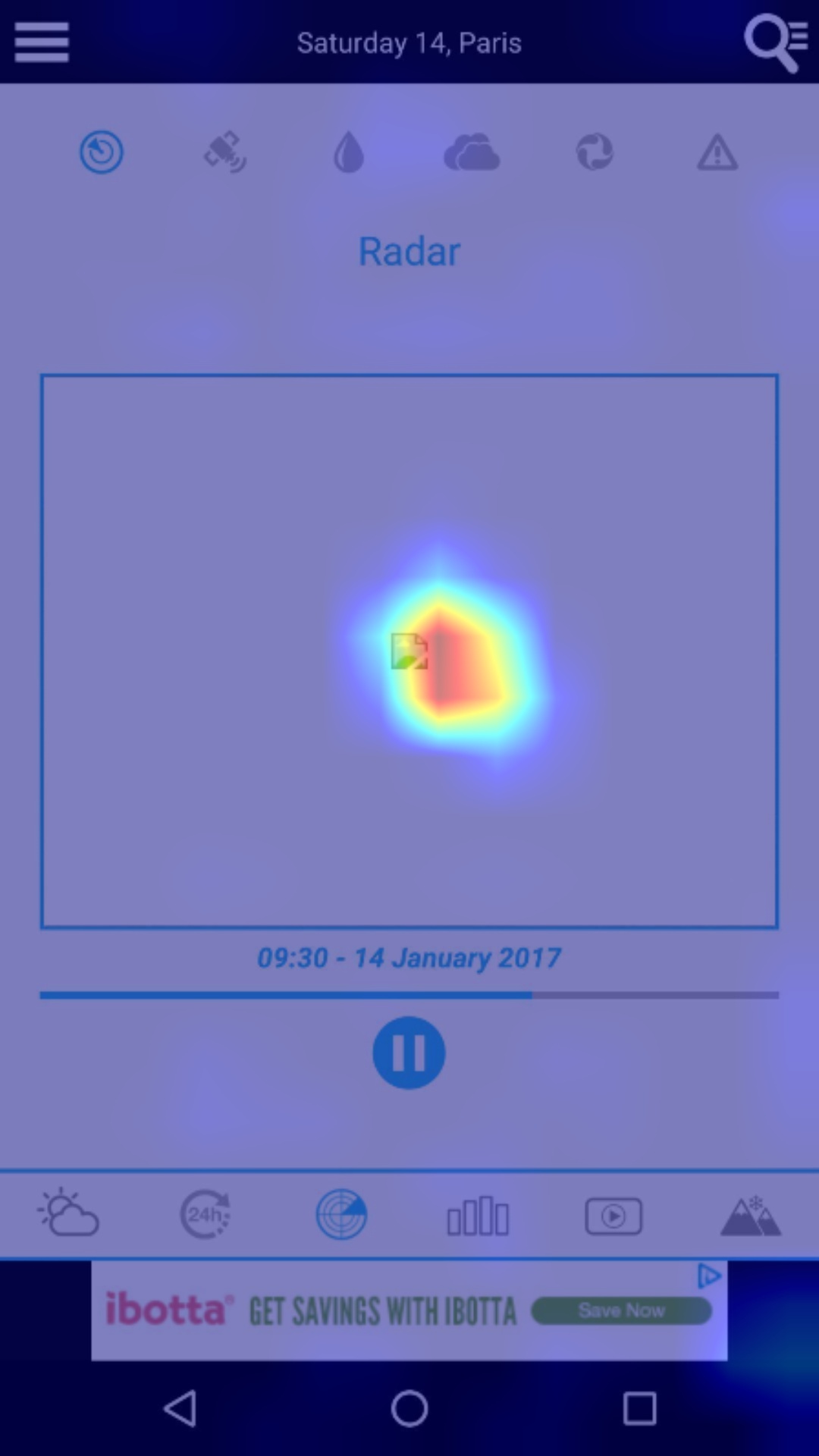

Supplement: Supplemental Information 1 — Use main file UI repair [file peerj-cs-10-2028-s001.zip › MUI Repair code and Data/missing image/13cam.jpg]

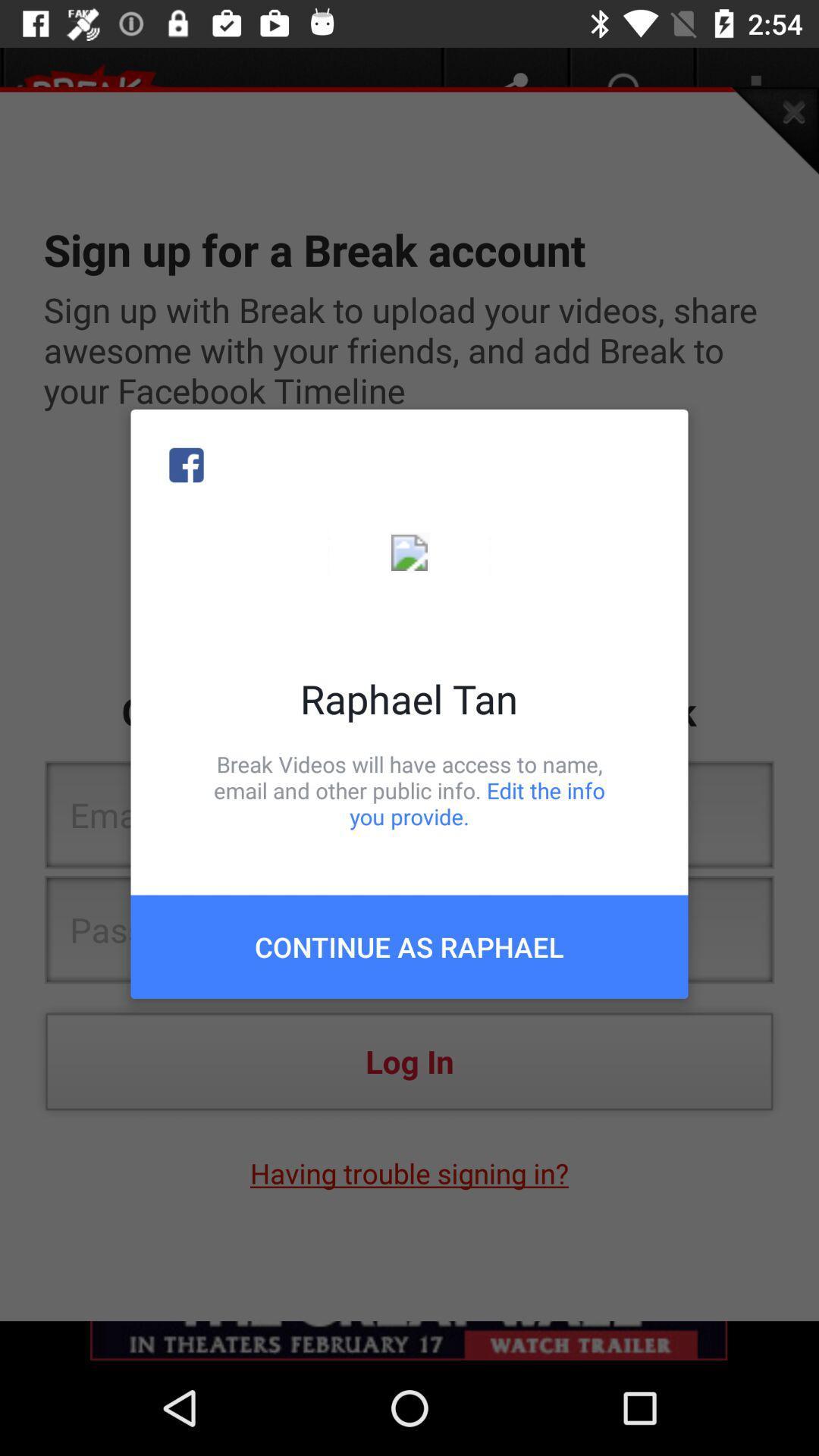

Supplement: Supplemental Information 1 — Use main file UI repair [file peerj-cs-10-2028-s001.zip › MUI Repair code and Data/missing image/14.jpg]

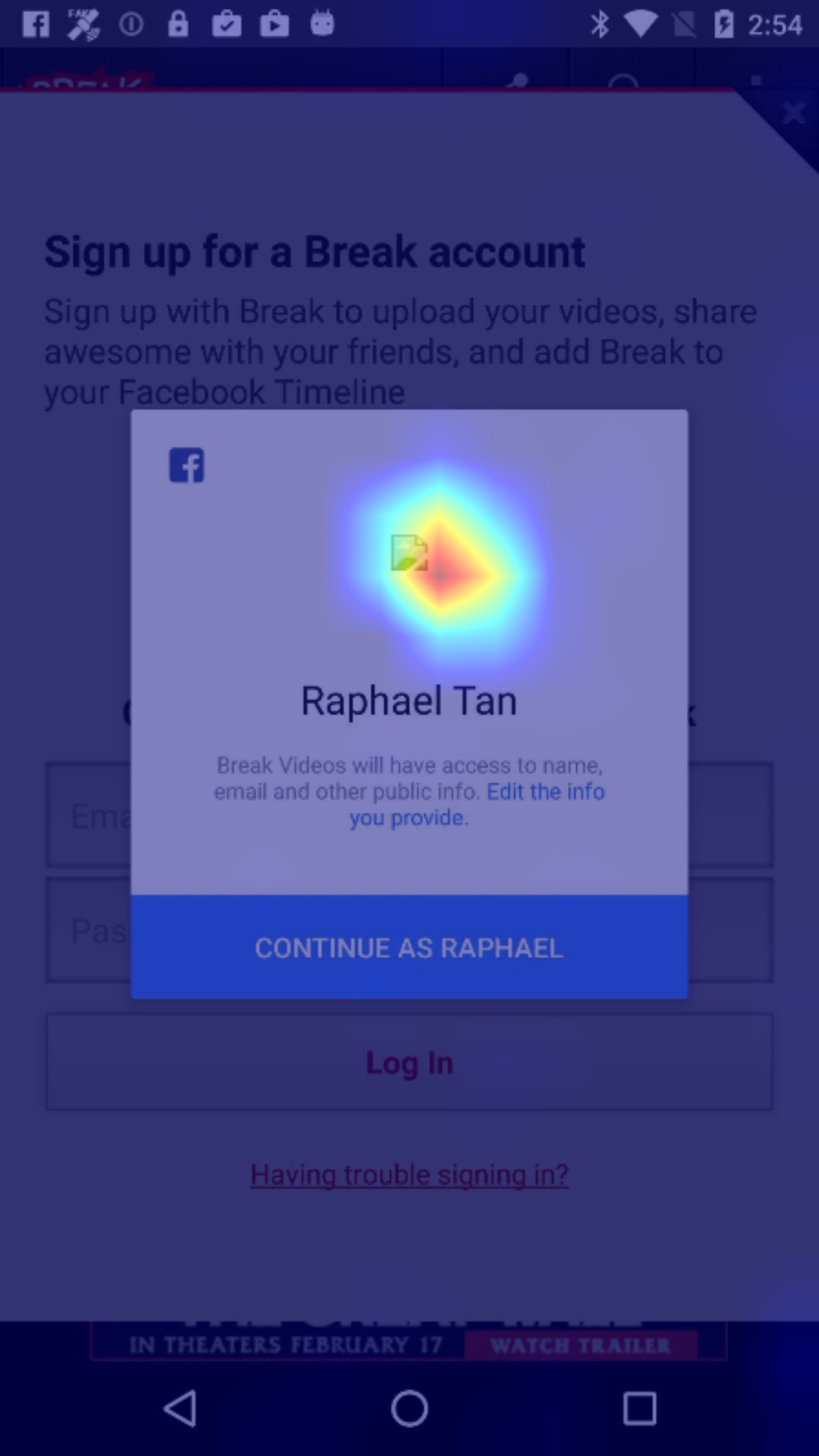

Supplement: Supplemental Information 1 — Use main file UI repair [file peerj-cs-10-2028-s001.zip › MUI Repair code and Data/missing image/14cam.jpg]

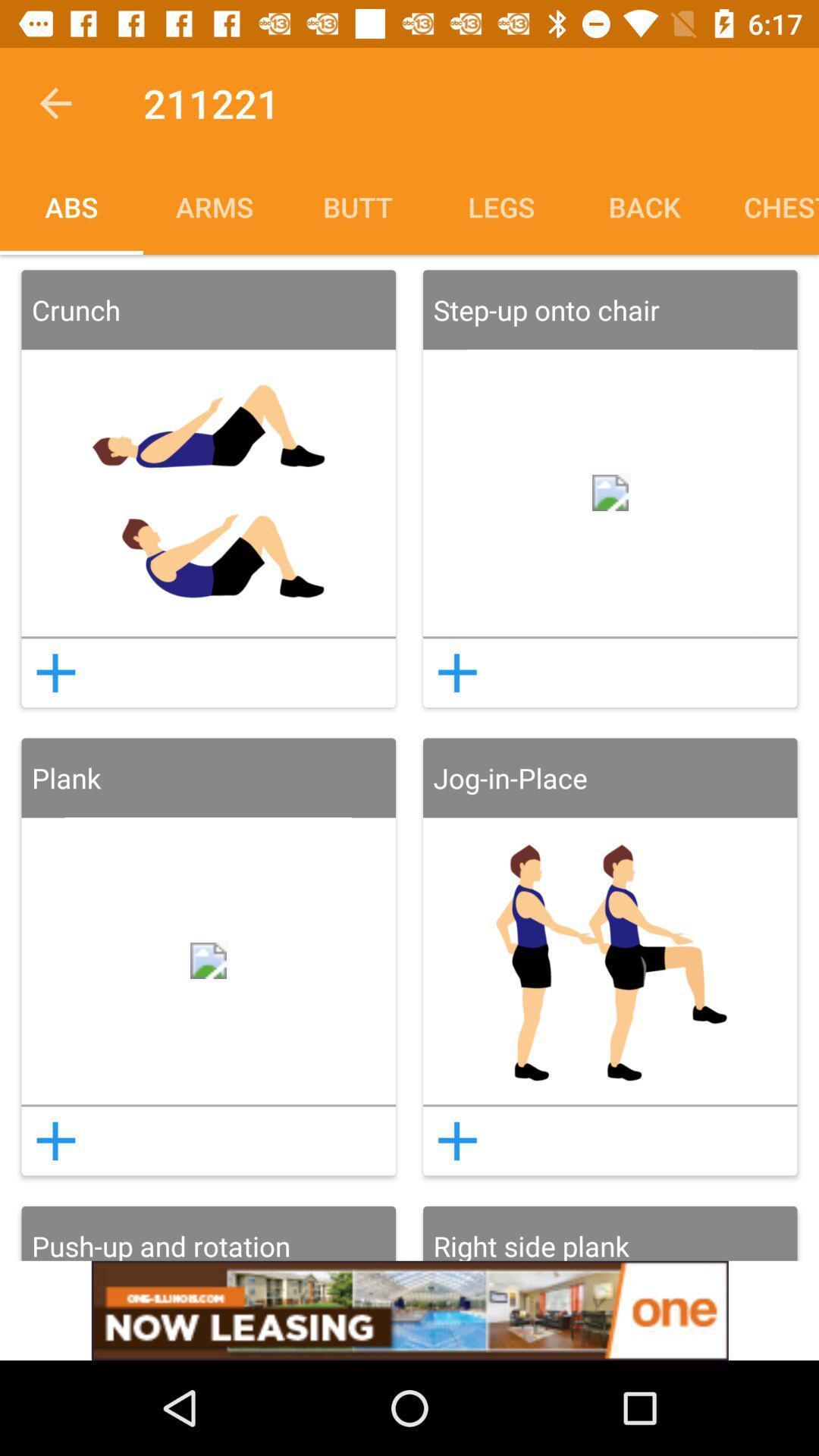

Supplement: Supplemental Information 1 — Use main file UI repair [file peerj-cs-10-2028-s001.zip › MUI Repair code and Data/missing image/15.jpg]

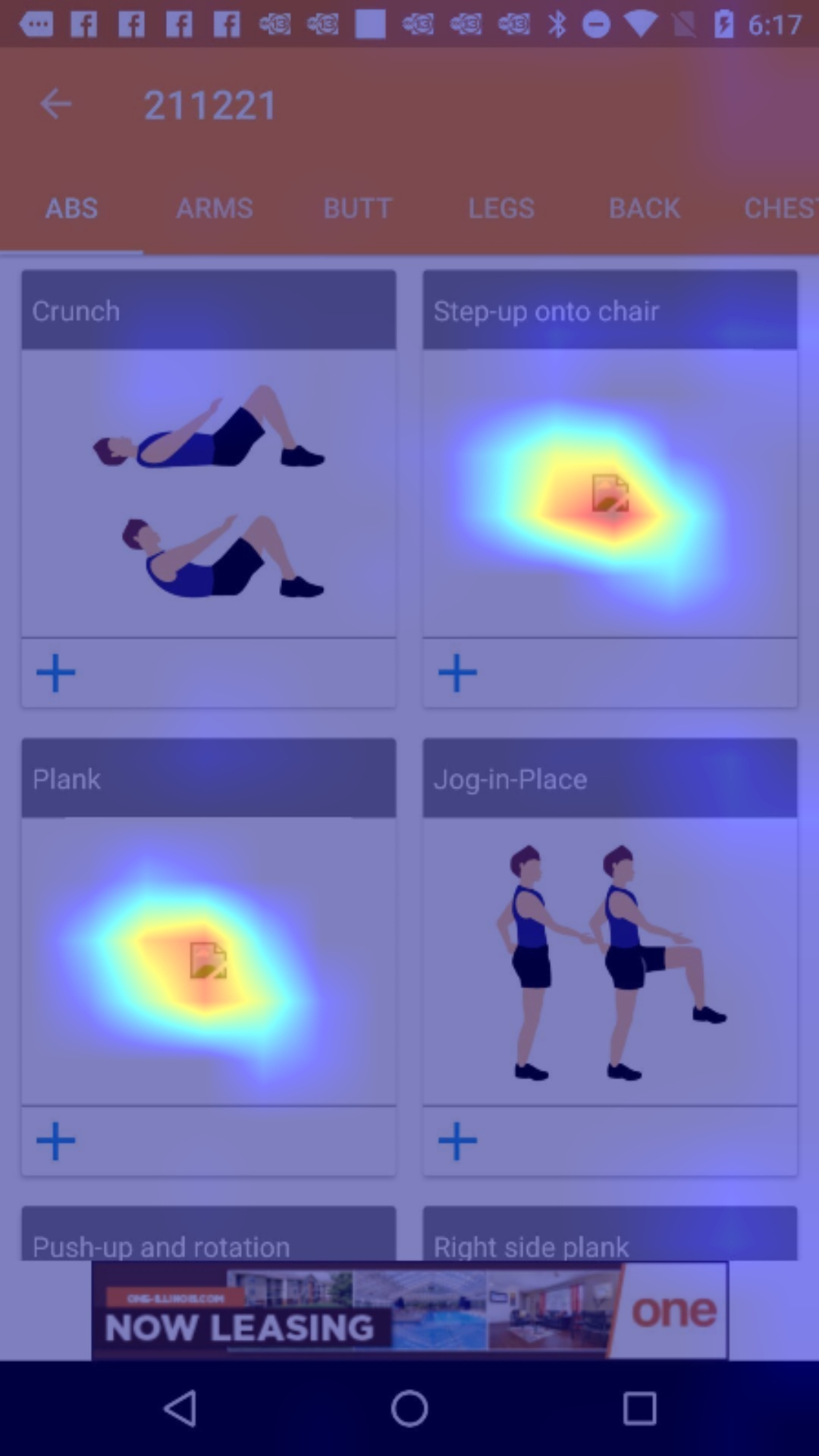

Supplement: Supplemental Information 1 — Use main file UI repair [file peerj-cs-10-2028-s001.zip › MUI Repair code and Data/missing image/15cam.jpg]

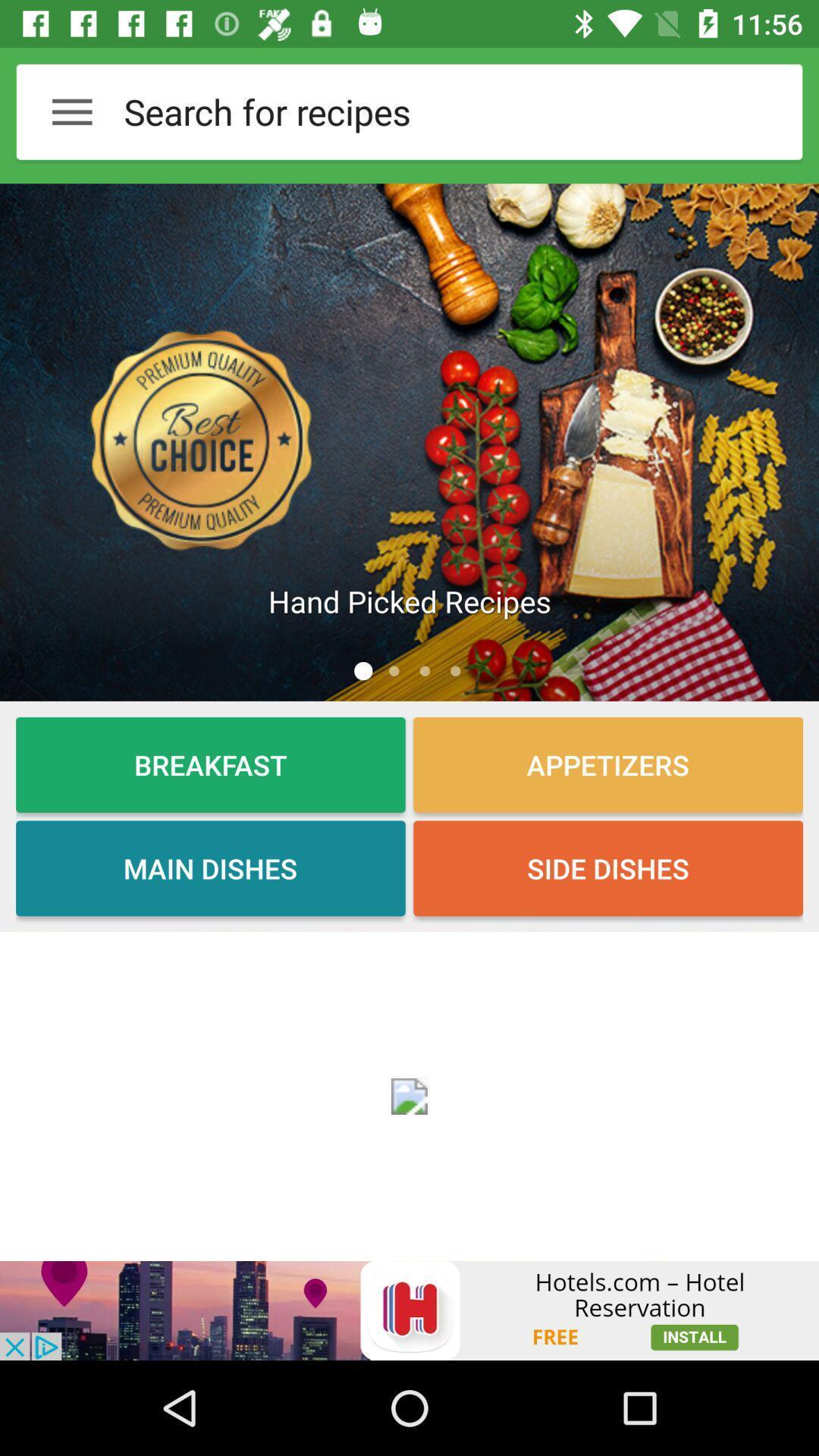

Supplement: Supplemental Information 1 — Use main file UI repair [file peerj-cs-10-2028-s001.zip › MUI Repair code and Data/missing image/16.jpg]

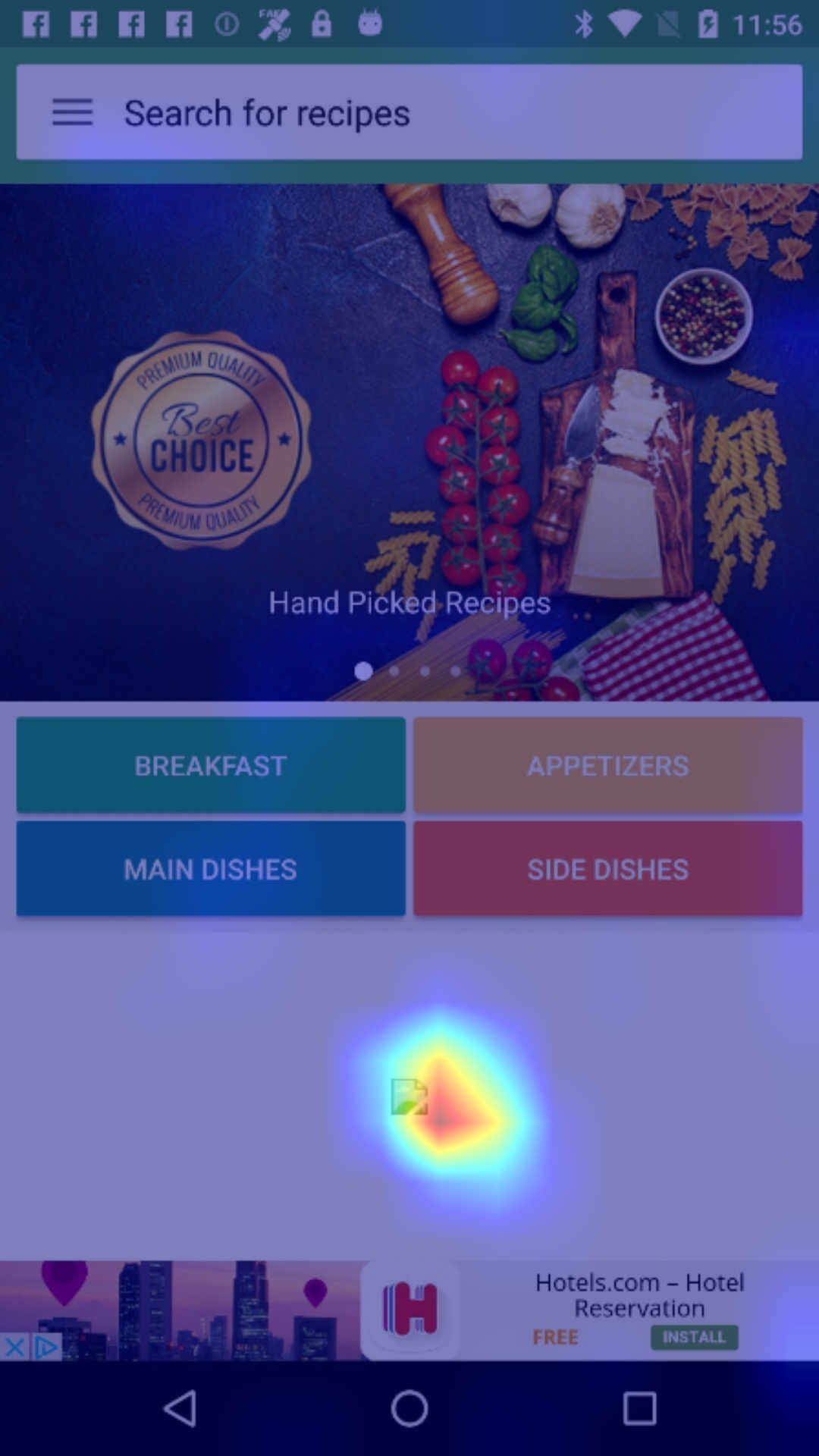

Supplement: Supplemental Information 1 — Use main file UI repair [file peerj-cs-10-2028-s001.zip › MUI Repair code and Data/missing image/16cam.jpg]

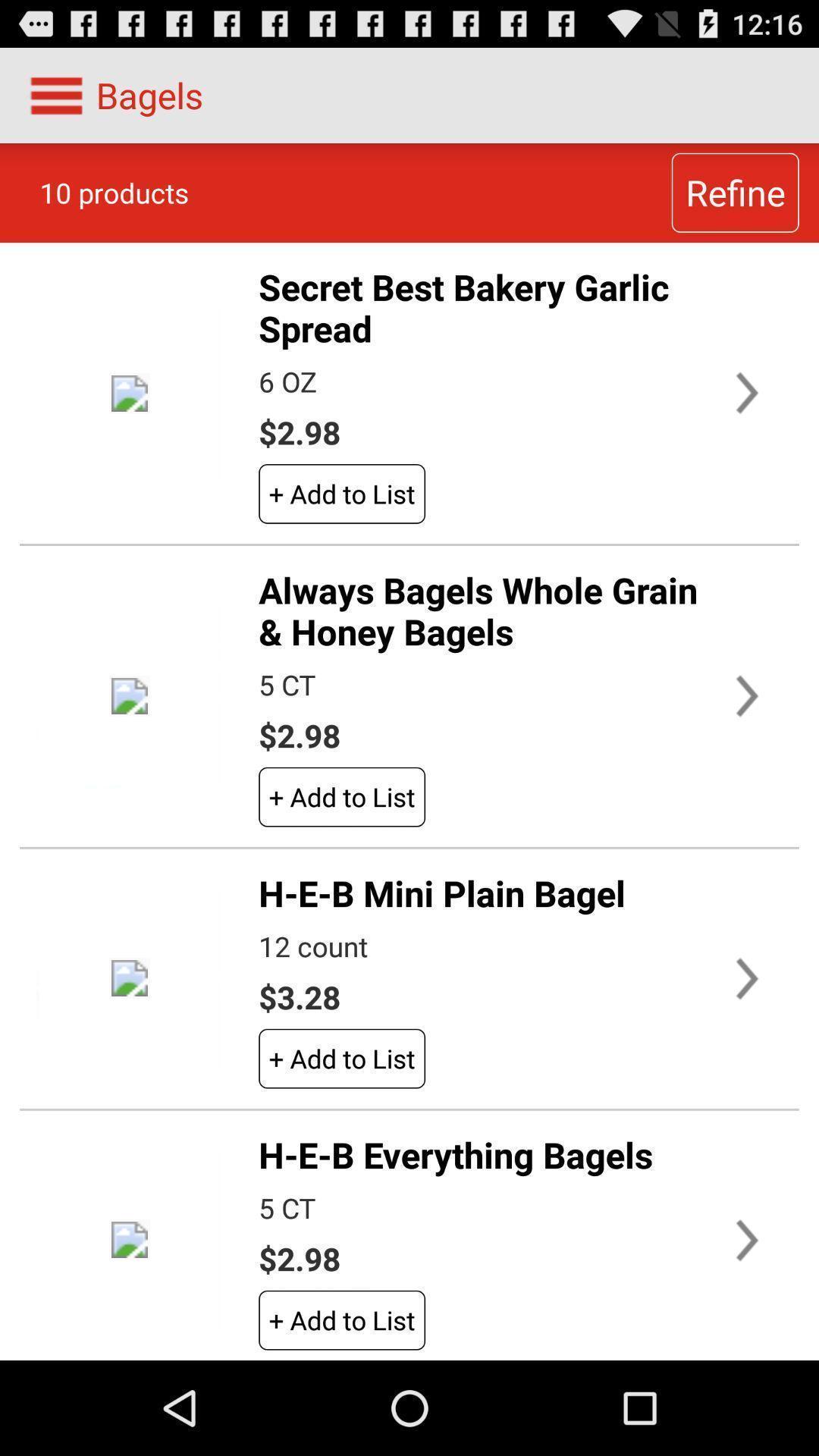

Supplement: Supplemental Information 1 — Use main file UI repair [file peerj-cs-10-2028-s001.zip › MUI Repair code and Data/missing image/17.jpg]

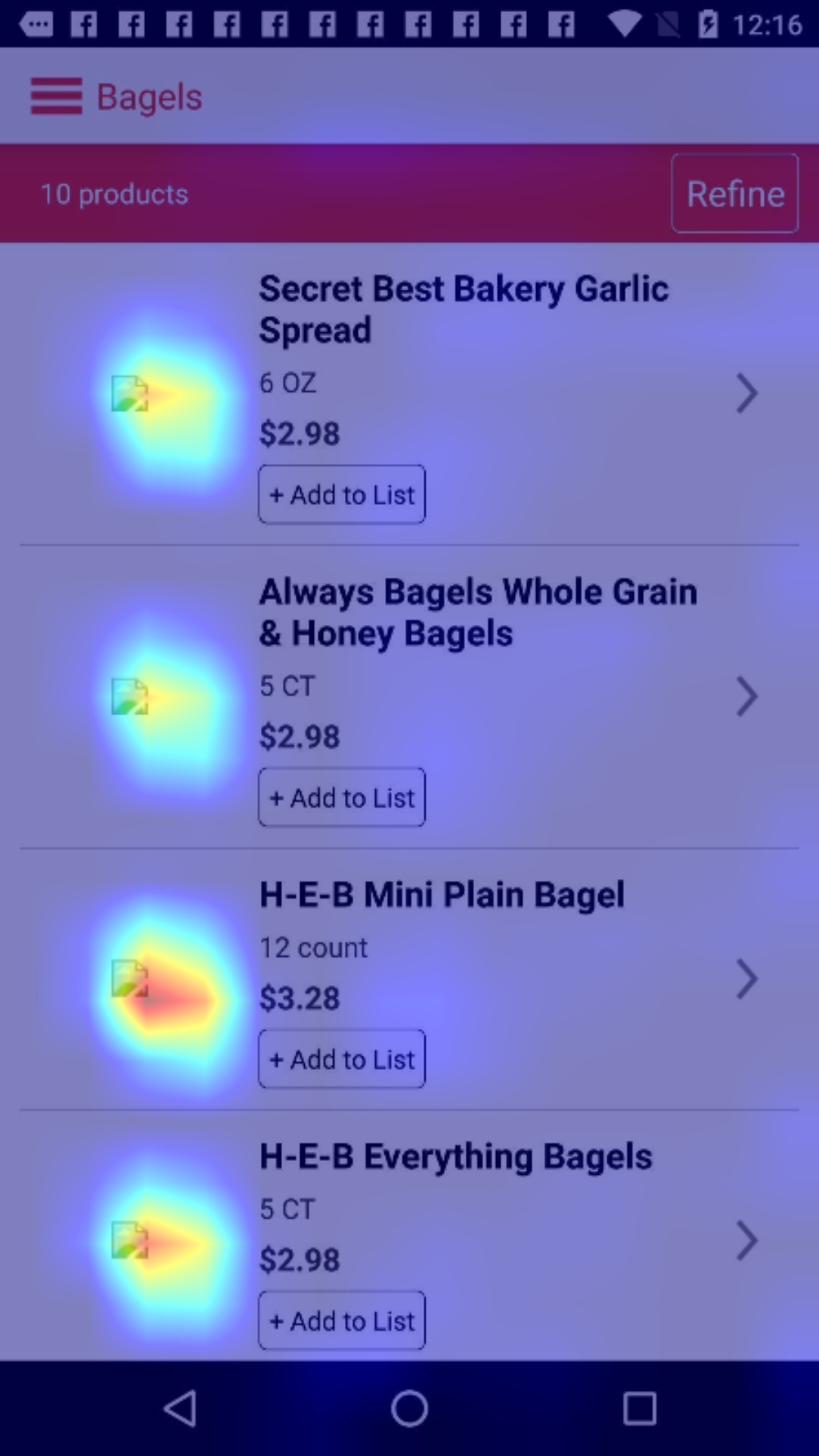

Supplement: Supplemental Information 1 — Use main file UI repair [file peerj-cs-10-2028-s001.zip › MUI Repair code and Data/missing image/17cam.jpg]

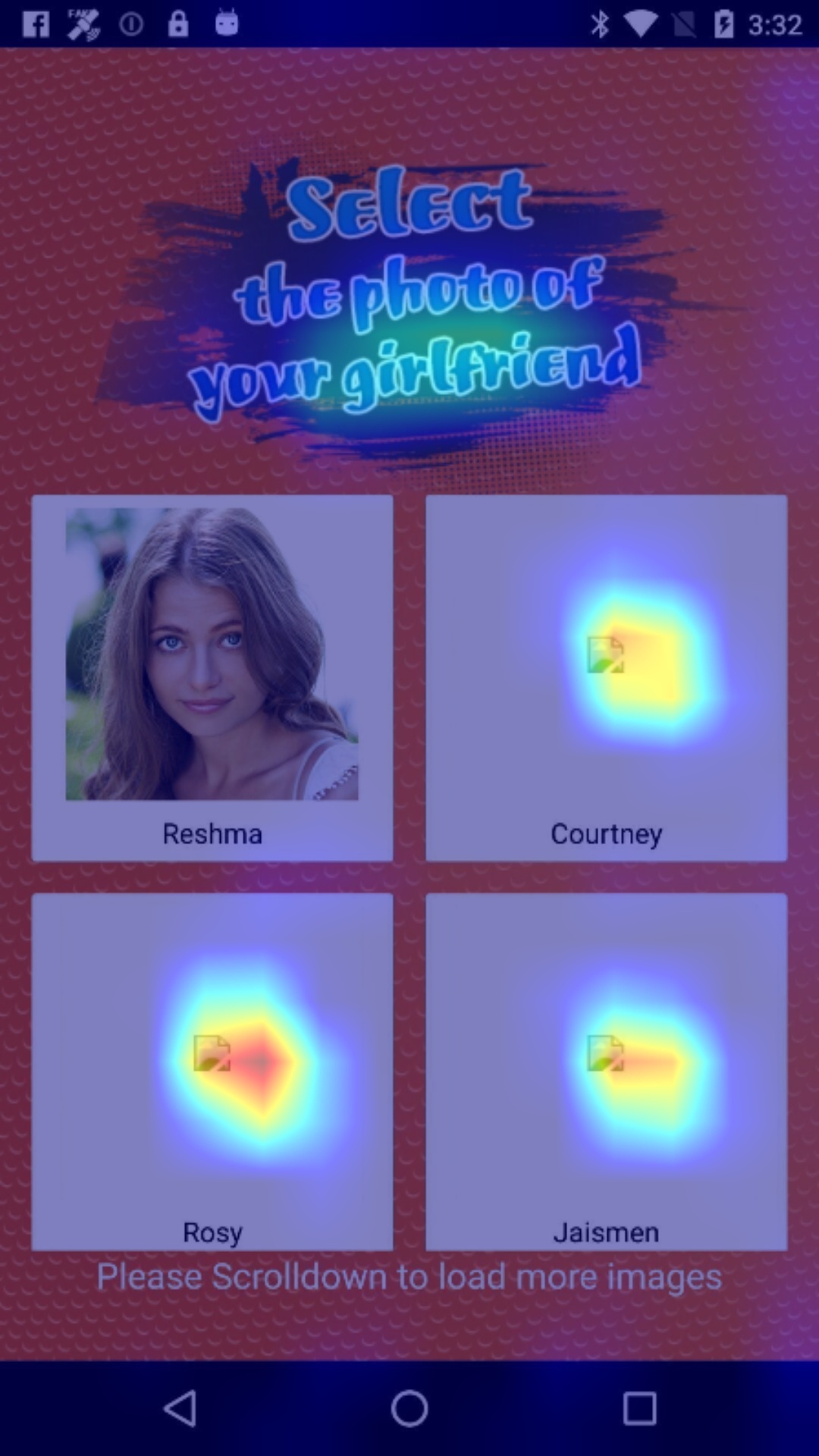

Supplement: Supplemental Information 1 — Use main file UI repair [file peerj-cs-10-2028-s001.zip › MUI Repair code and Data/missing image/1cam.jpg]

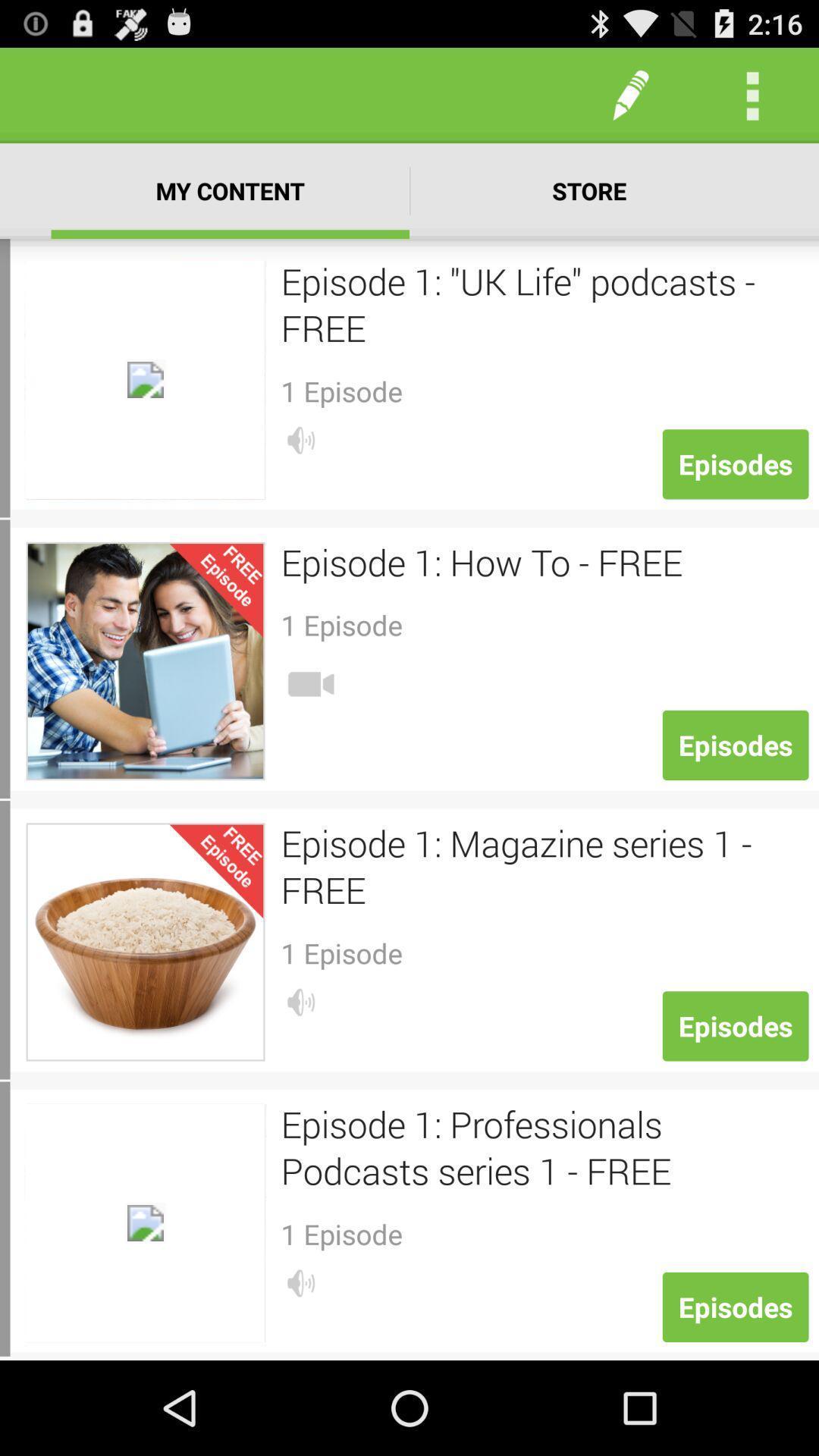

Supplement: Supplemental Information 1 — Use main file UI repair [file peerj-cs-10-2028-s001.zip › MUI Repair code and Data/missing image/2.jpg]

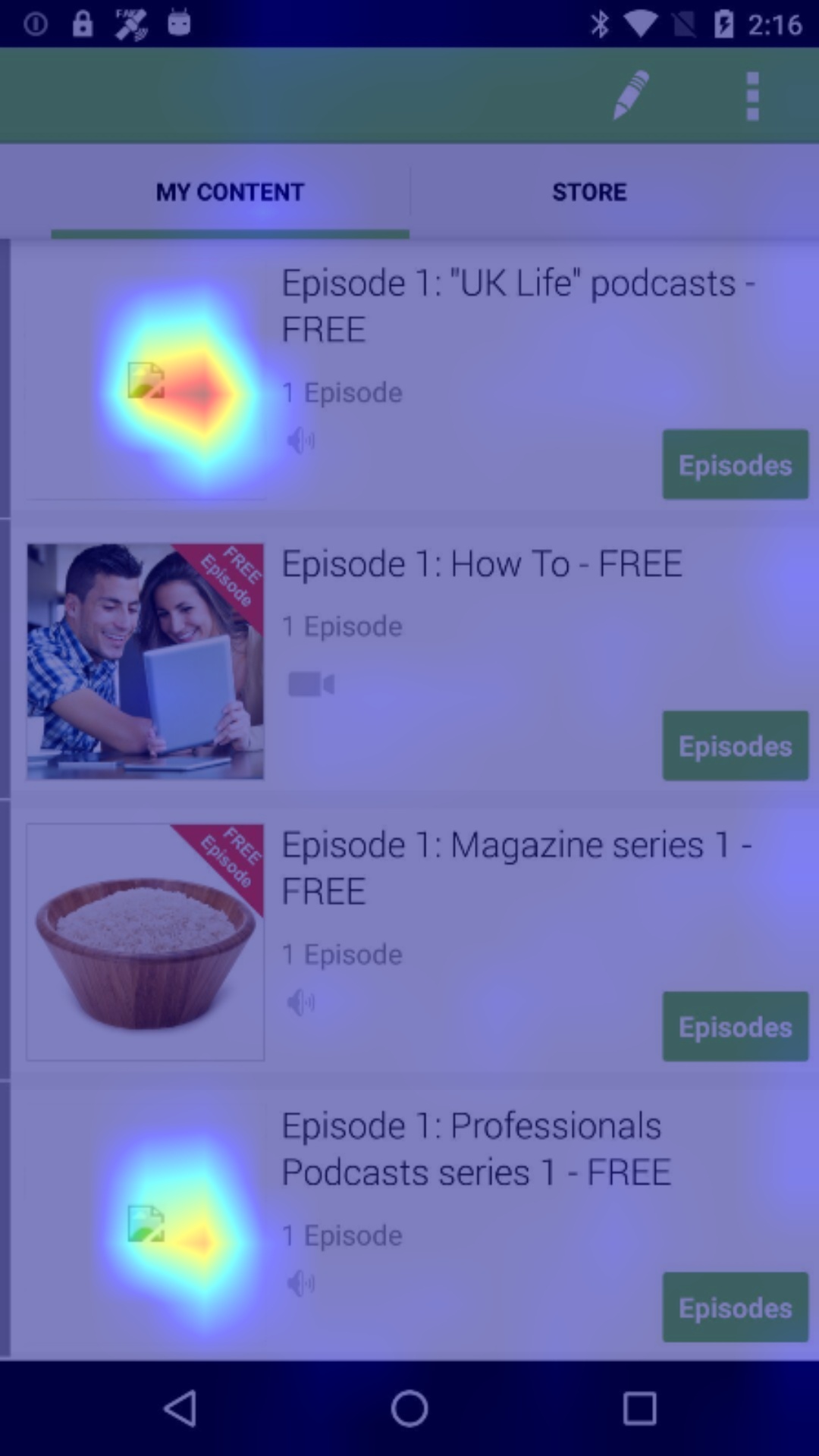

Supplement: Supplemental Information 1 — Use main file UI repair [file peerj-cs-10-2028-s001.zip › MUI Repair code and Data/missing image/2cam.jpg]

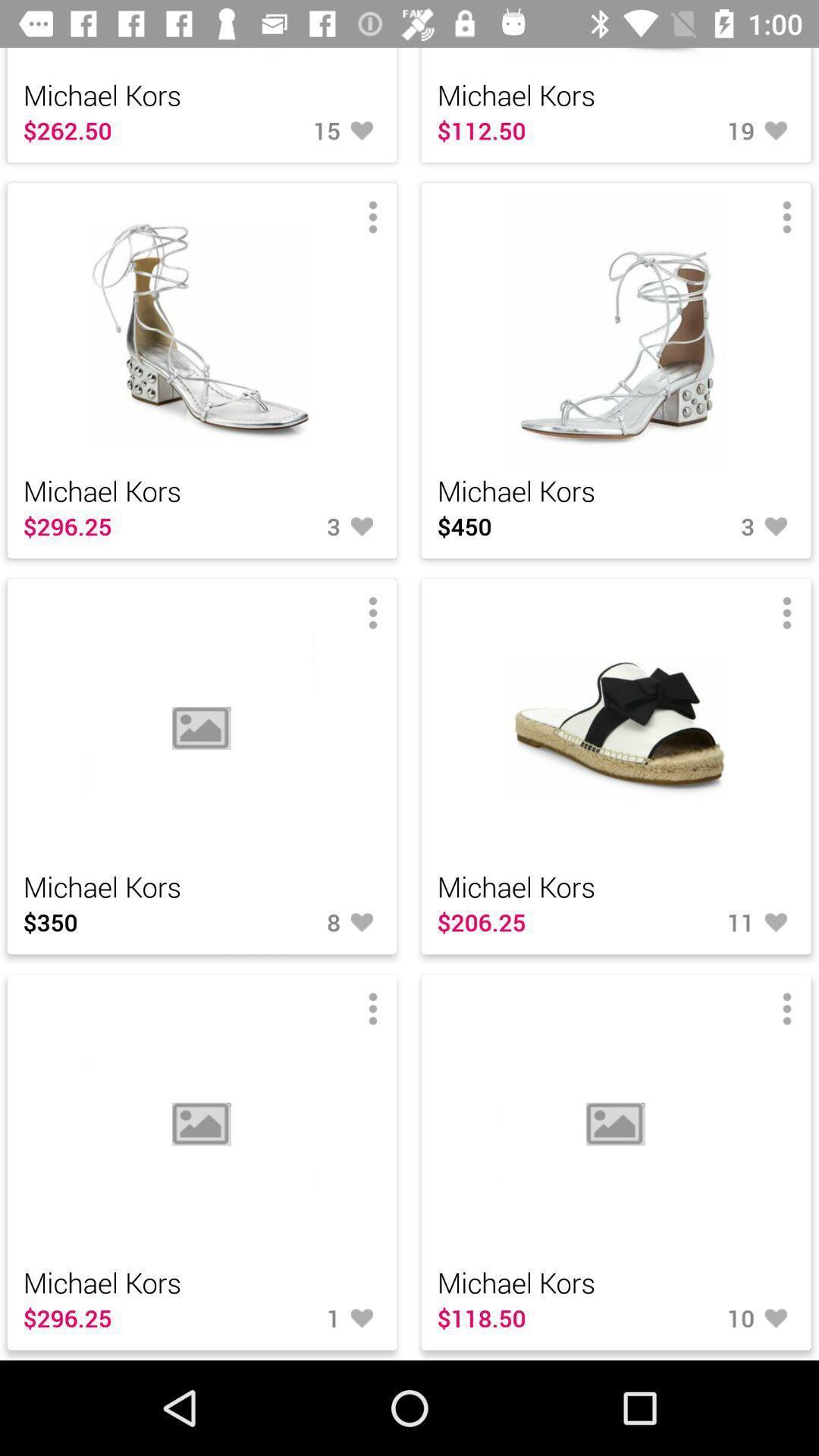

Supplement: Supplemental Information 1 — Use main file UI repair [file peerj-cs-10-2028-s001.zip › MUI Repair code and Data/missing image/3.jpg]

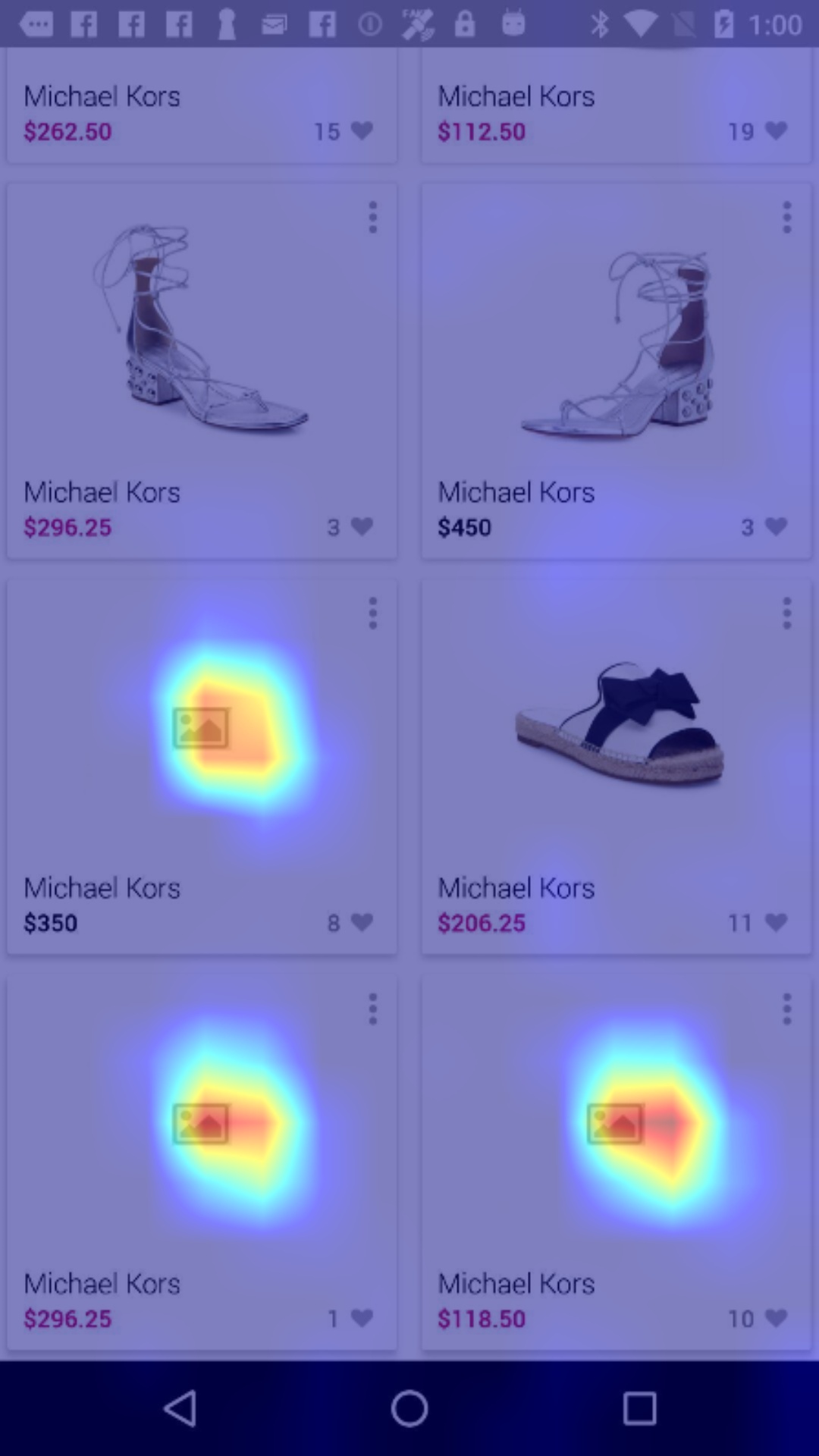

Supplement: Supplemental Information 1 — Use main file UI repair [file peerj-cs-10-2028-s001.zip › MUI Repair code and Data/missing image/3cam.jpg]

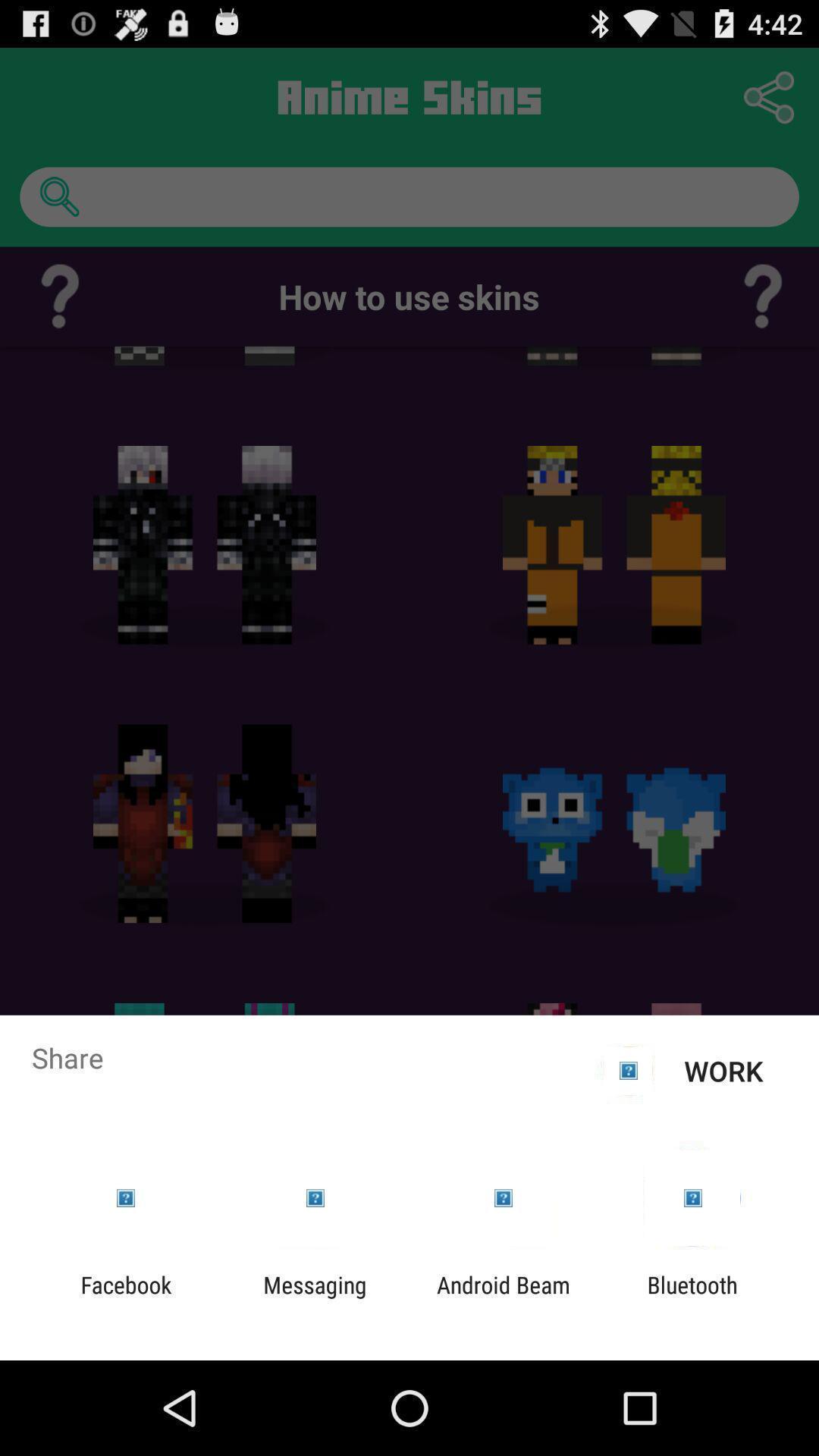

Supplement: Supplemental Information 1 — Use main file UI repair [file peerj-cs-10-2028-s001.zip › MUI Repair code and Data/missing image/4.jpg]

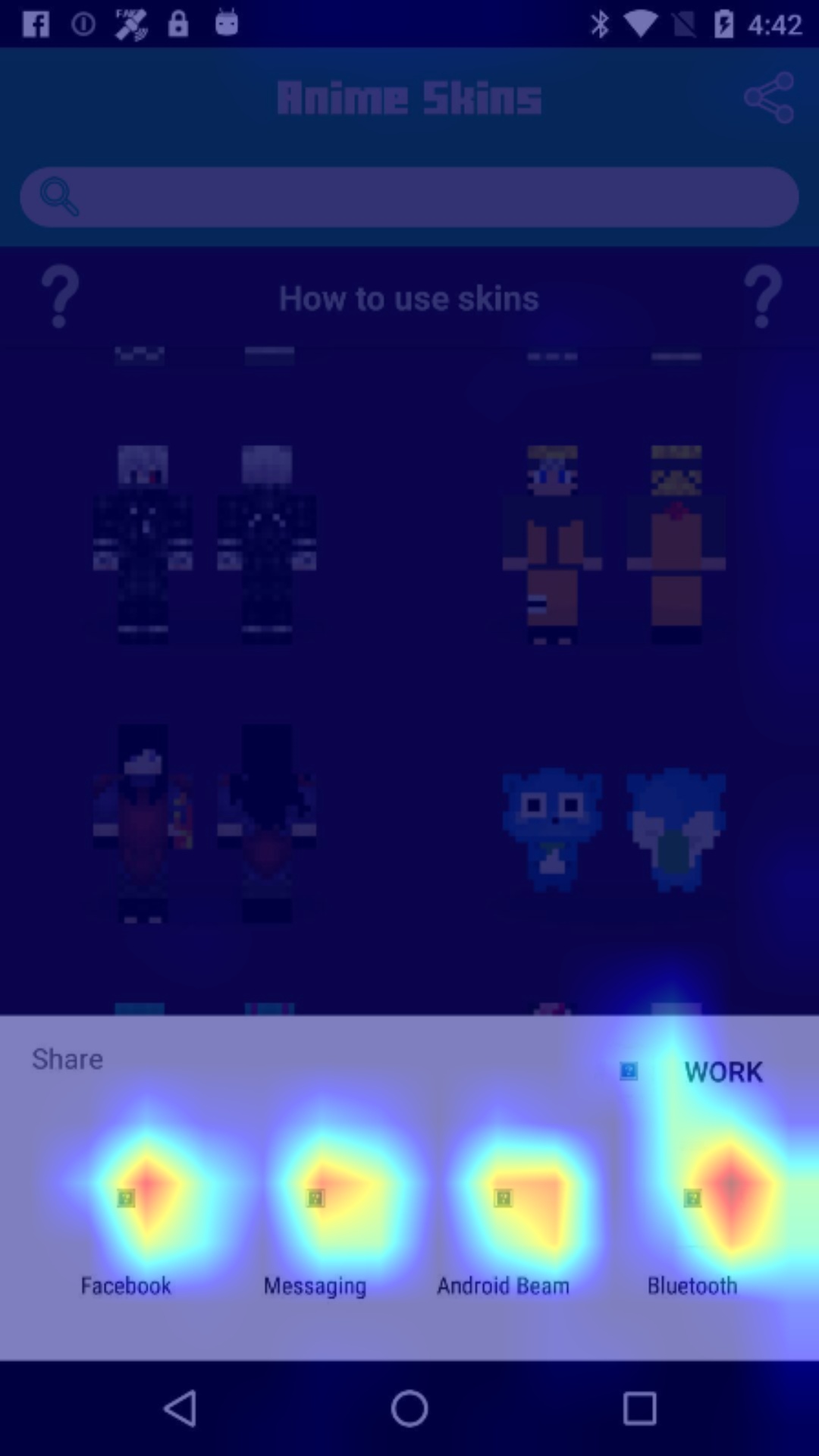

Supplement: Supplemental Information 1 — Use main file UI repair [file peerj-cs-10-2028-s001.zip › MUI Repair code and Data/missing image/4cam.jpg]

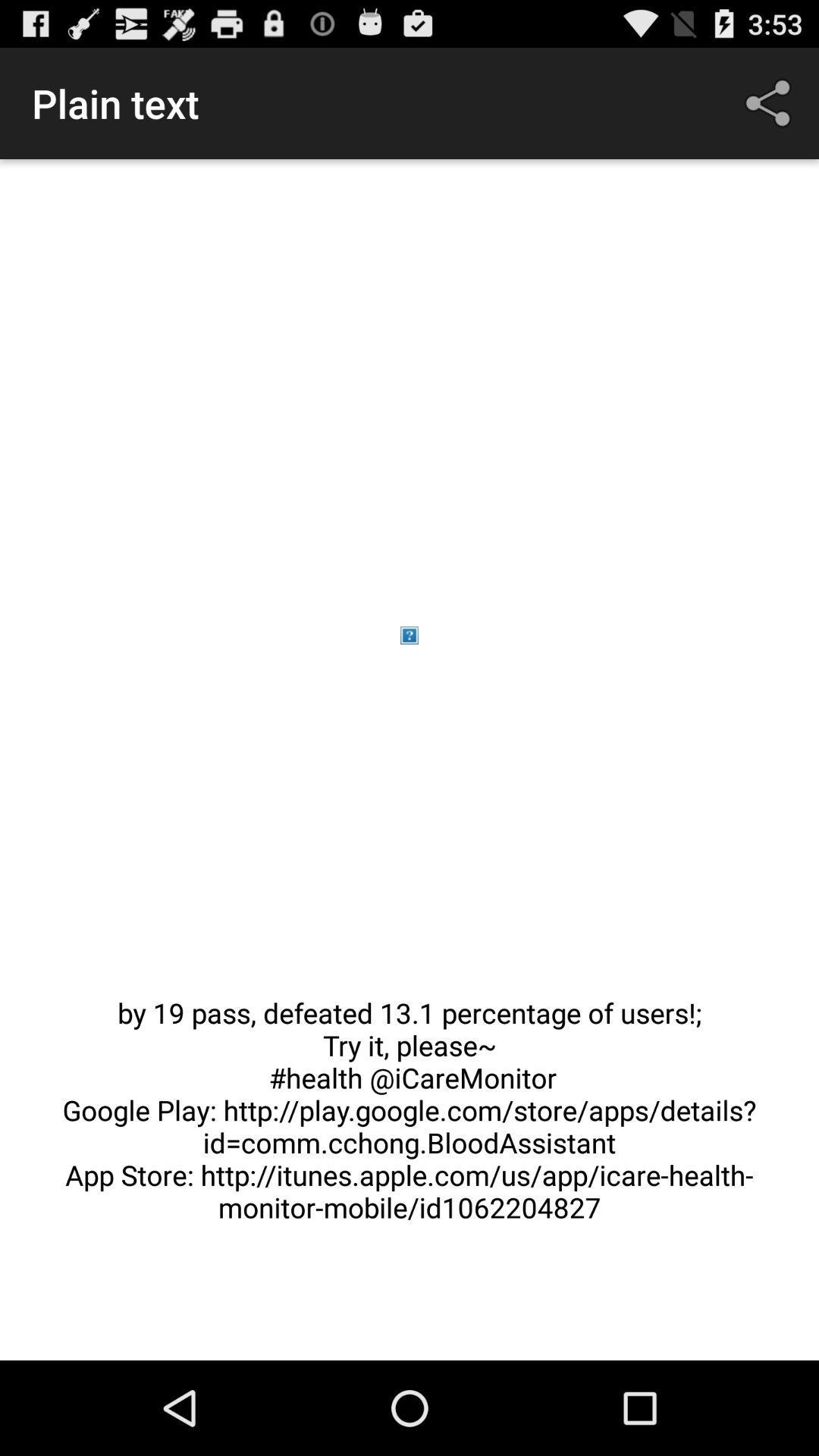

Supplement: Supplemental Information 1 — Use main file UI repair [file peerj-cs-10-2028-s001.zip › MUI Repair code and Data/missing image/5.jpg]

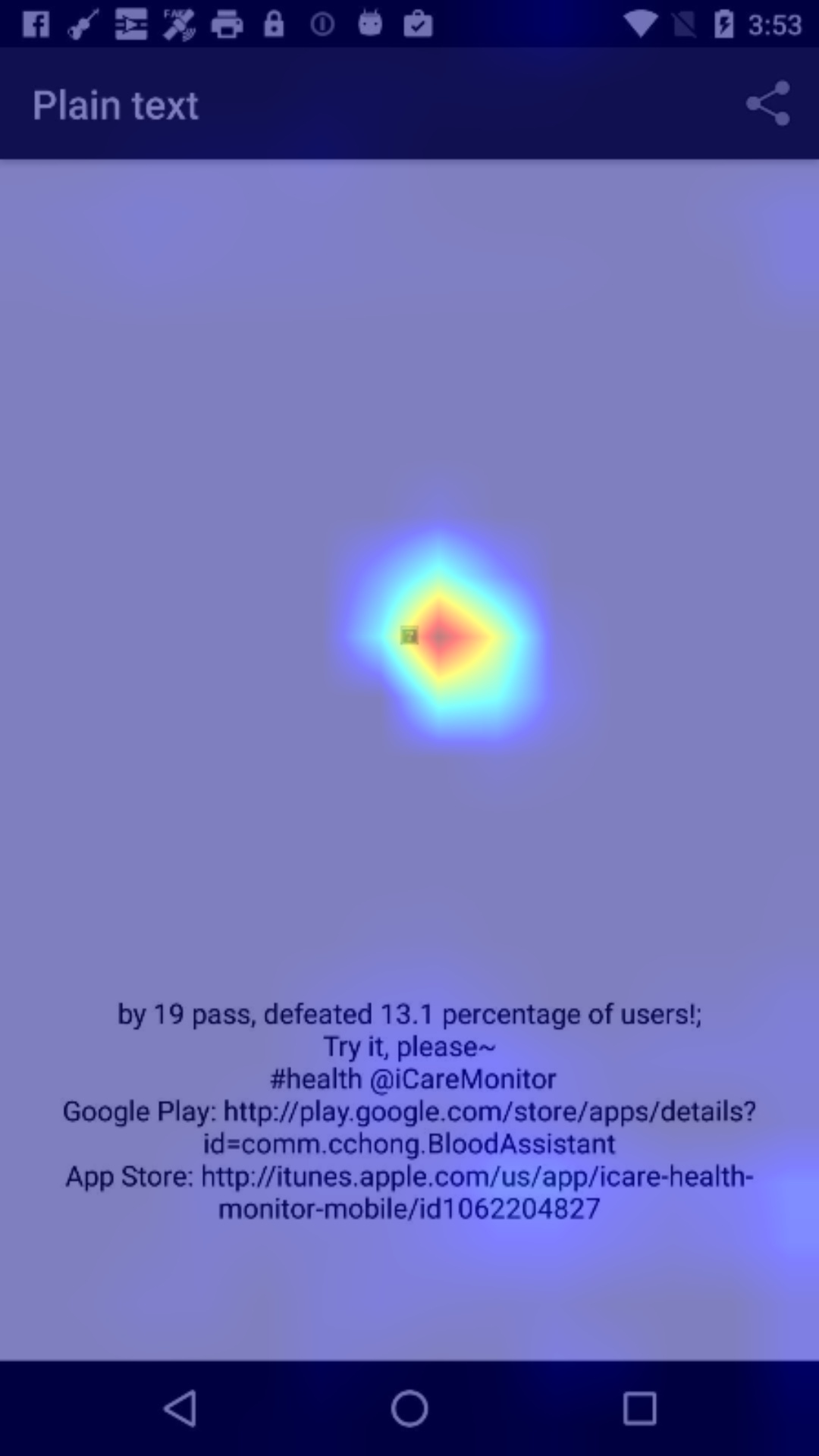

Supplement: Supplemental Information 1 — Use main file UI repair [file peerj-cs-10-2028-s001.zip › MUI Repair code and Data/missing image/5cam.jpg]

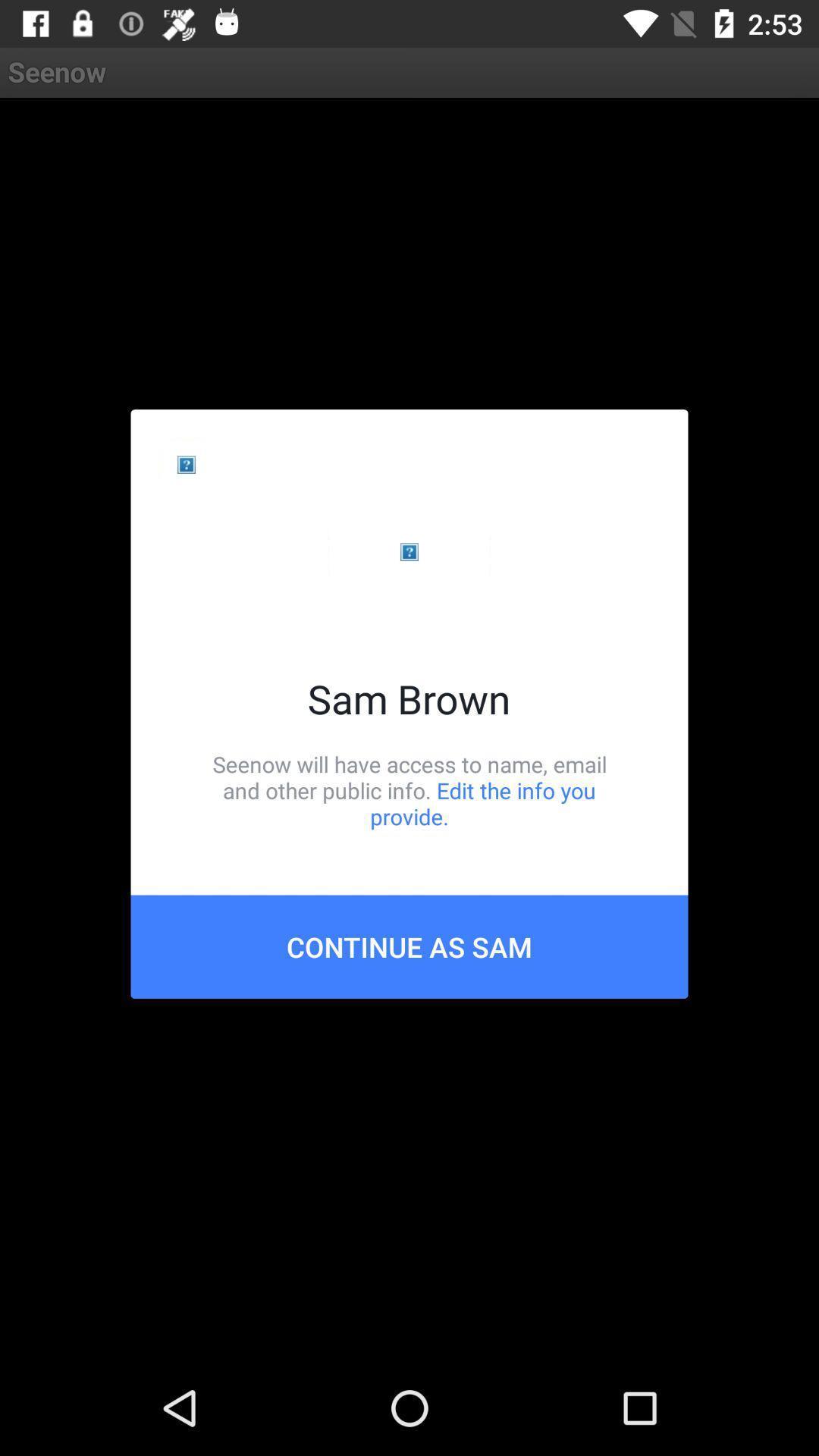

Supplement: Supplemental Information 1 — Use main file UI repair [file peerj-cs-10-2028-s001.zip › MUI Repair code and Data/missing image/6.jpg]

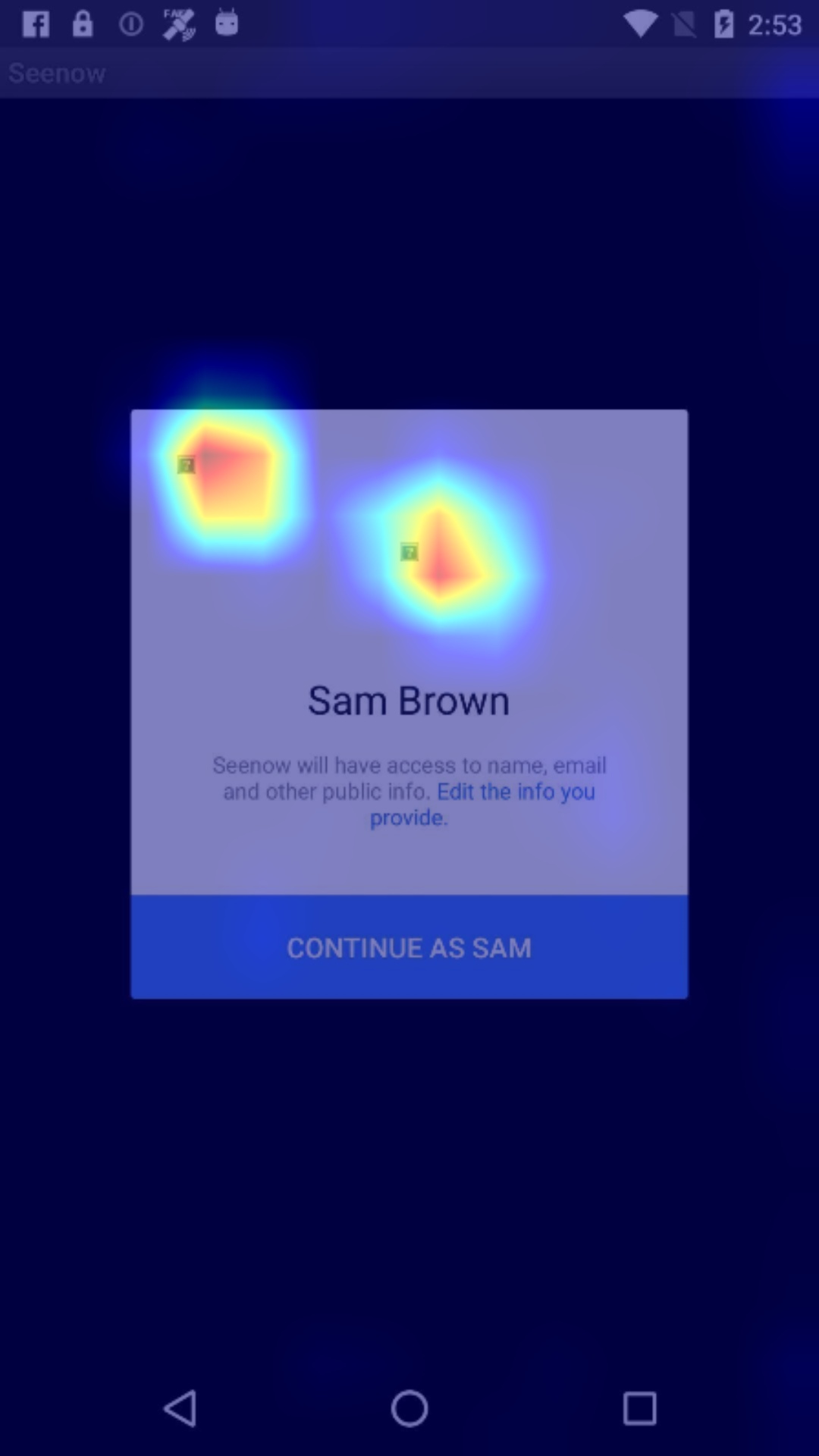

Supplement: Supplemental Information 1 — Use main file UI repair [file peerj-cs-10-2028-s001.zip › MUI Repair code and Data/missing image/6cam.jpg]

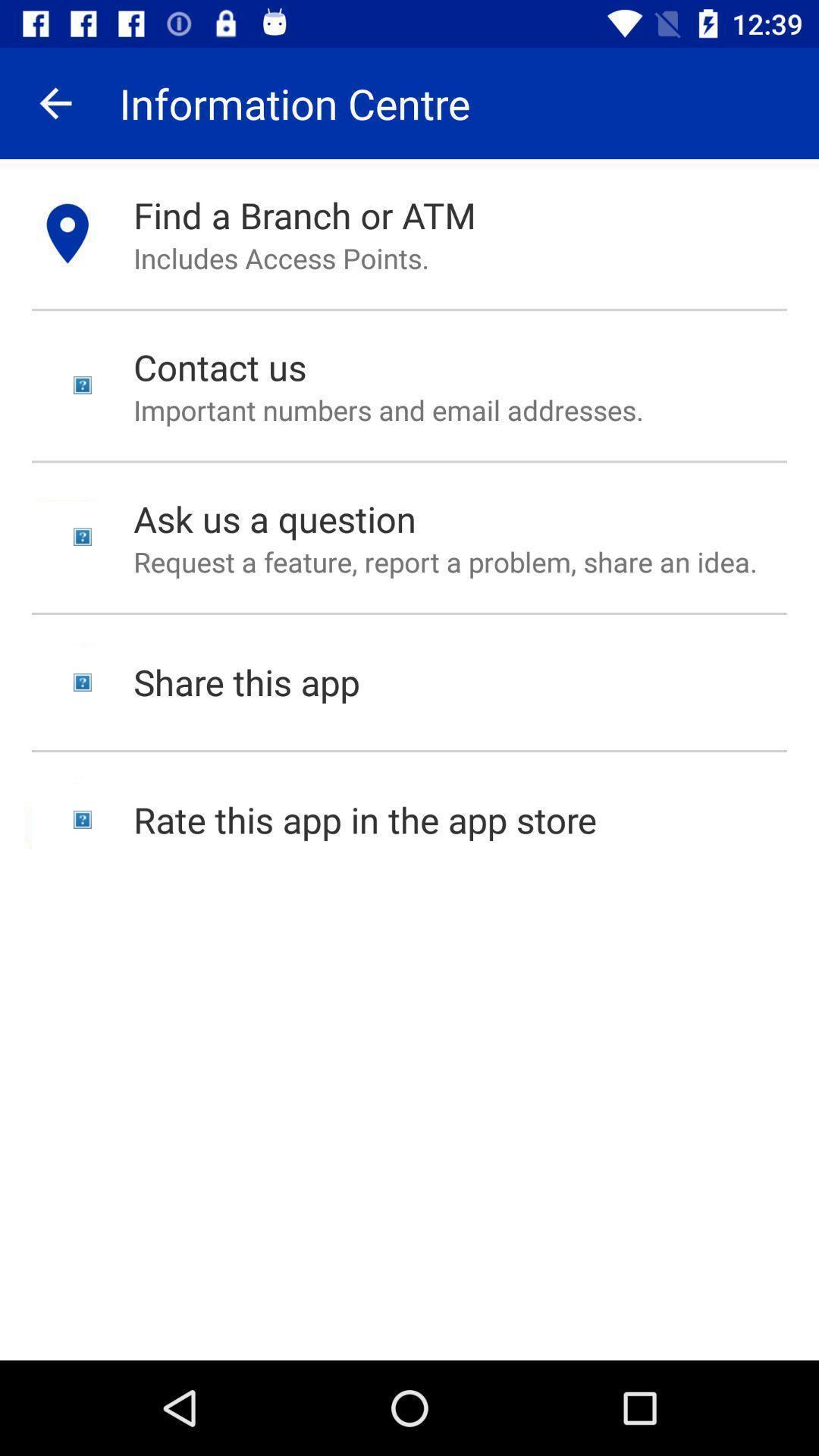

Supplement: Supplemental Information 1 — Use main file UI repair [file peerj-cs-10-2028-s001.zip › MUI Repair code and Data/missing image/7.jpg]

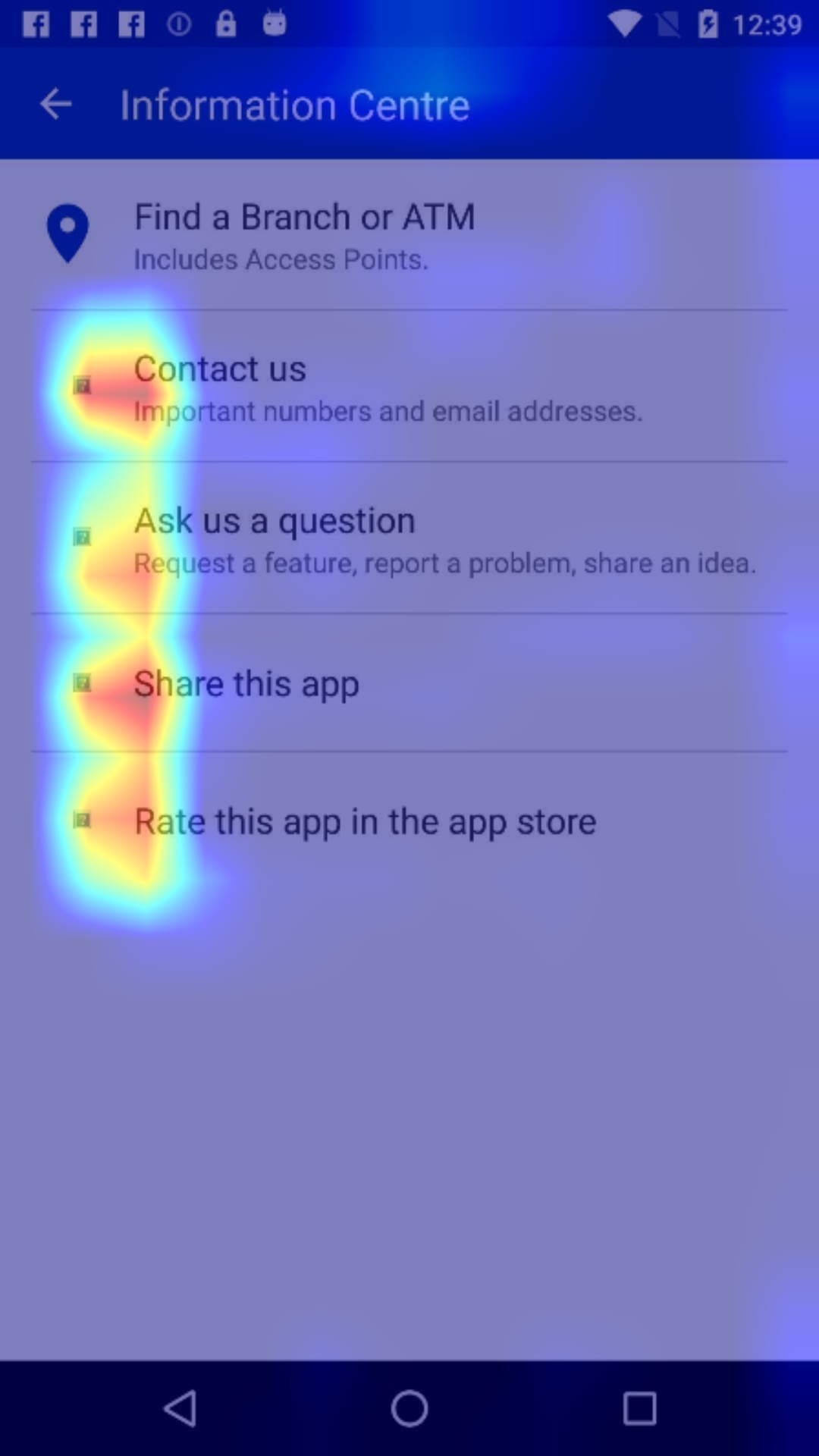

Supplement: Supplemental Information 1 — Use main file UI repair [file peerj-cs-10-2028-s001.zip › MUI Repair code and Data/missing image/7cam.jpg]

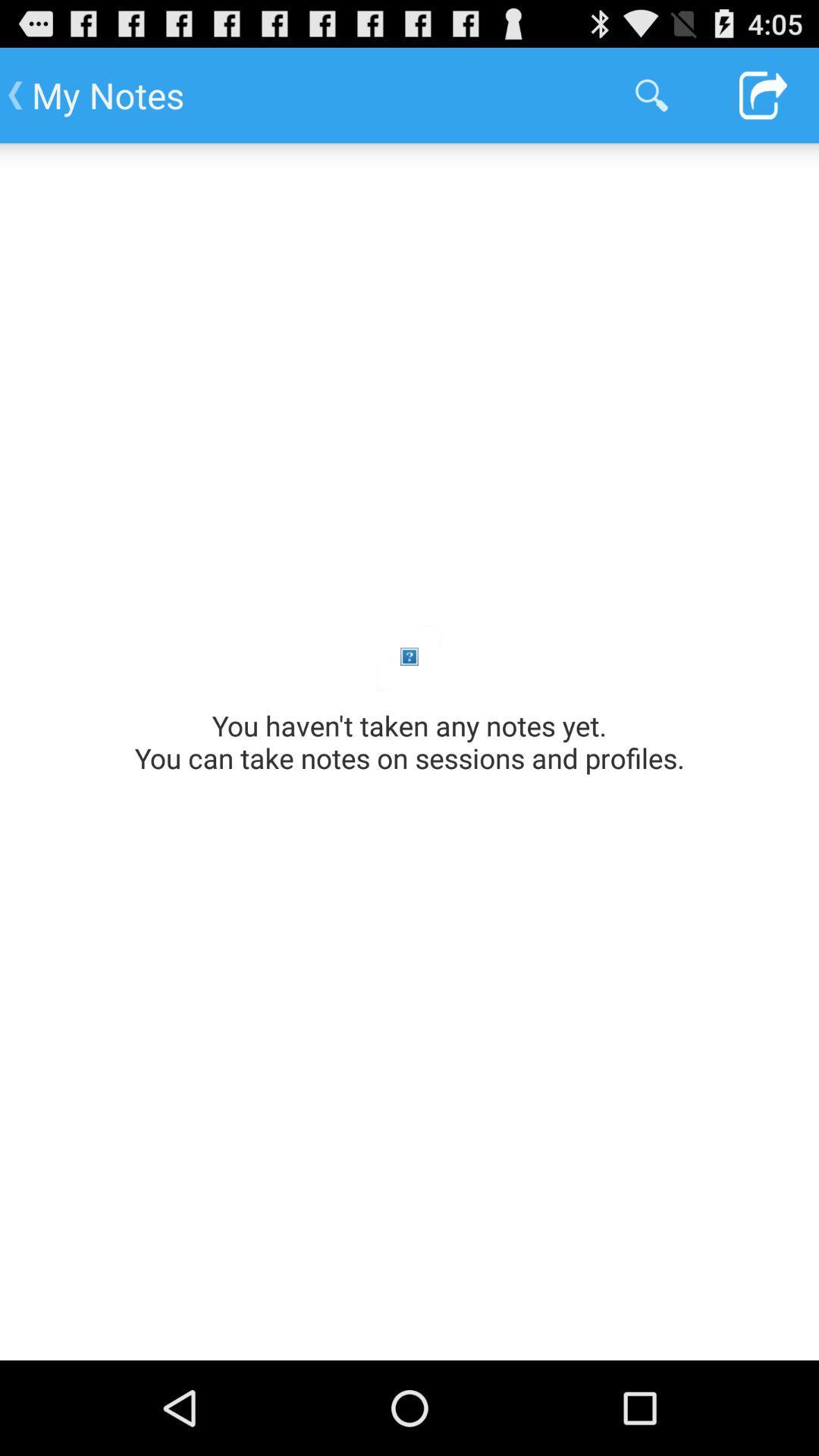

Supplement: Supplemental Information 1 — Use main file UI repair [file peerj-cs-10-2028-s001.zip › MUI Repair code and Data/missing image/8.jpg]

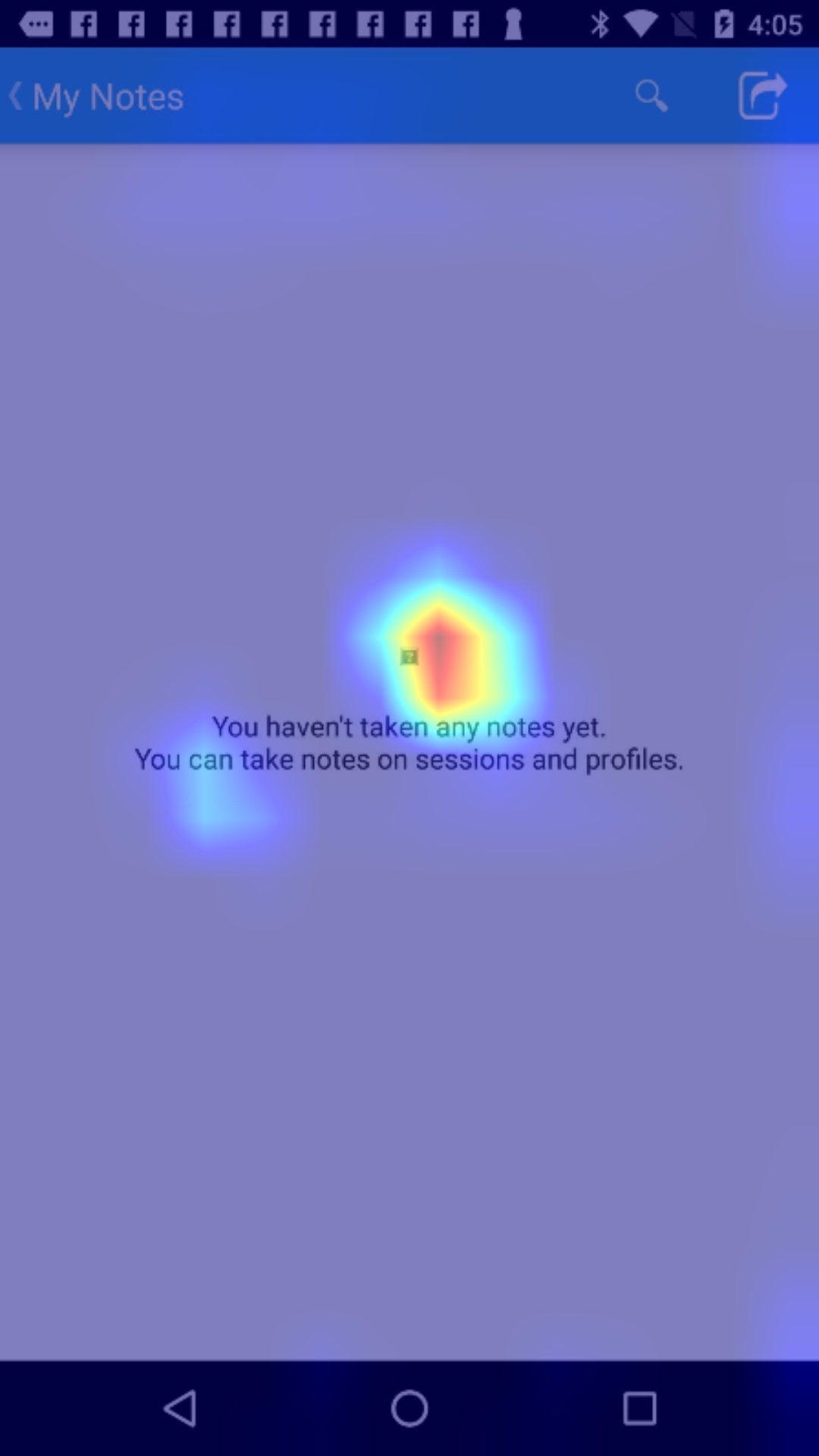

Supplement: Supplemental Information 1 — Use main file UI repair [file peerj-cs-10-2028-s001.zip › MUI Repair code and Data/missing image/8cam.jpg]

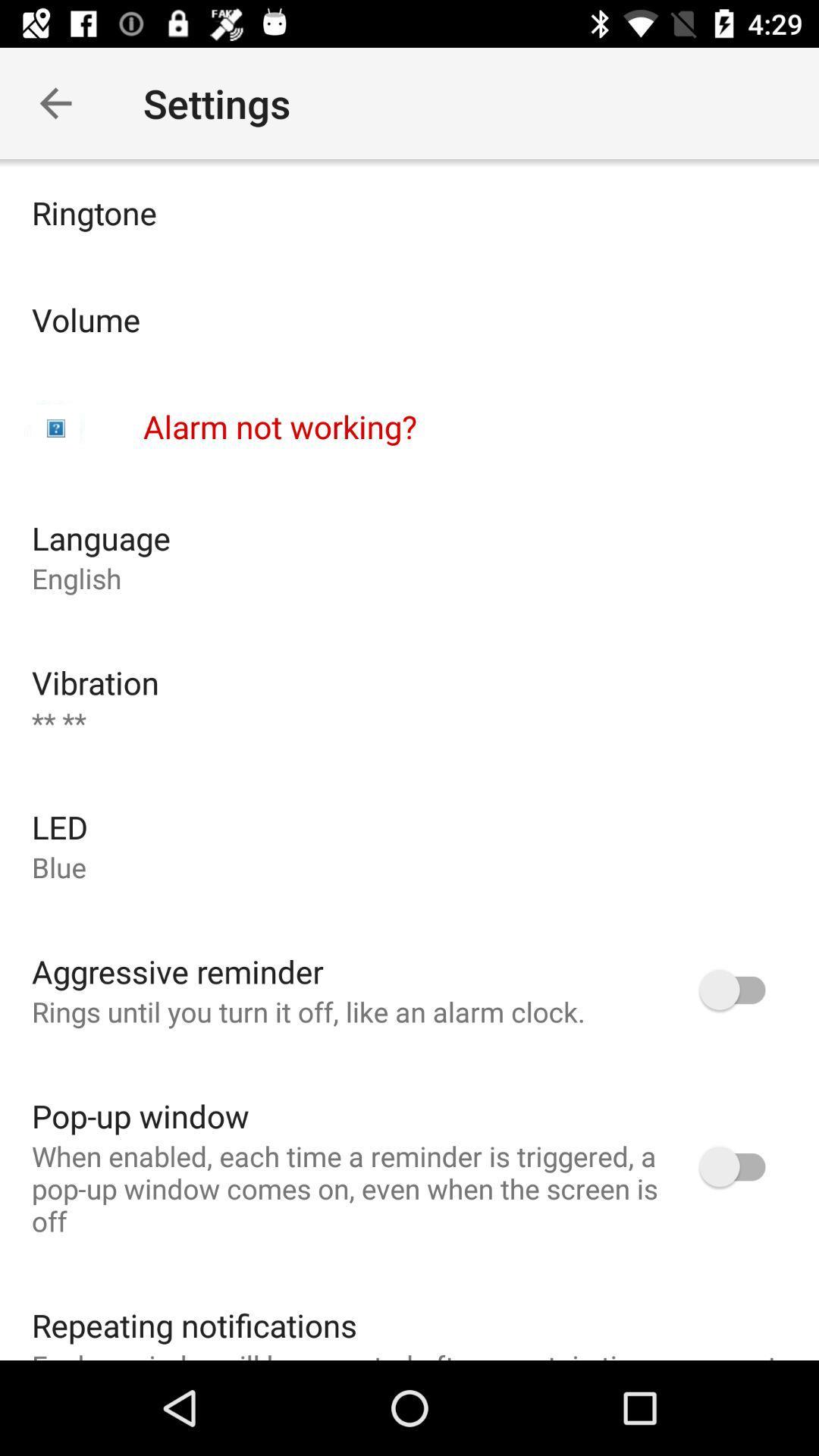

Supplement: Supplemental Information 1 — Use main file UI repair [file peerj-cs-10-2028-s001.zip › MUI Repair code and Data/missing image/9.jpg]

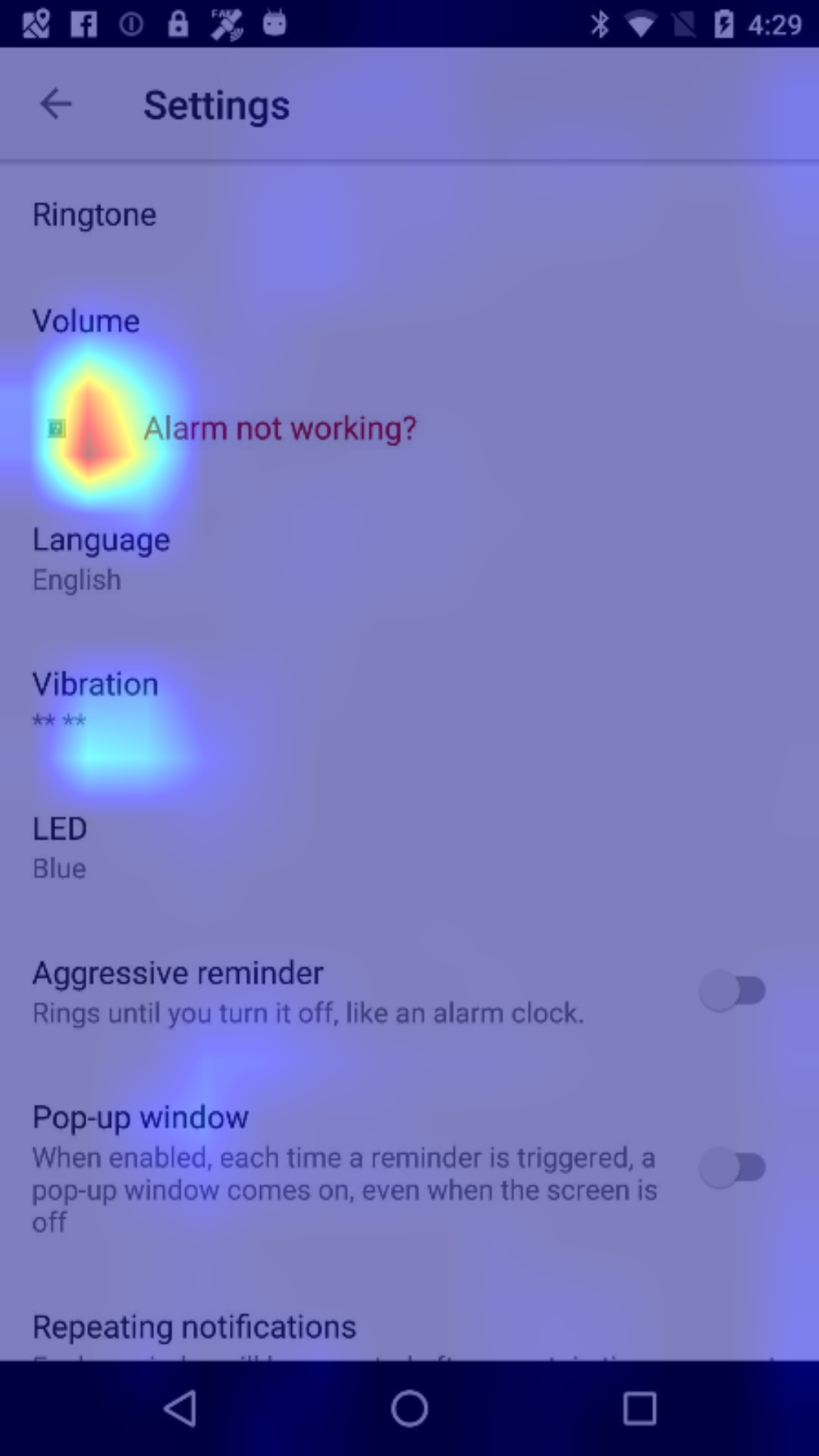

Supplement: Supplemental Information 1 — Use main file UI repair [file peerj-cs-10-2028-s001.zip › MUI Repair code and Data/missing image/9cam.jpg]

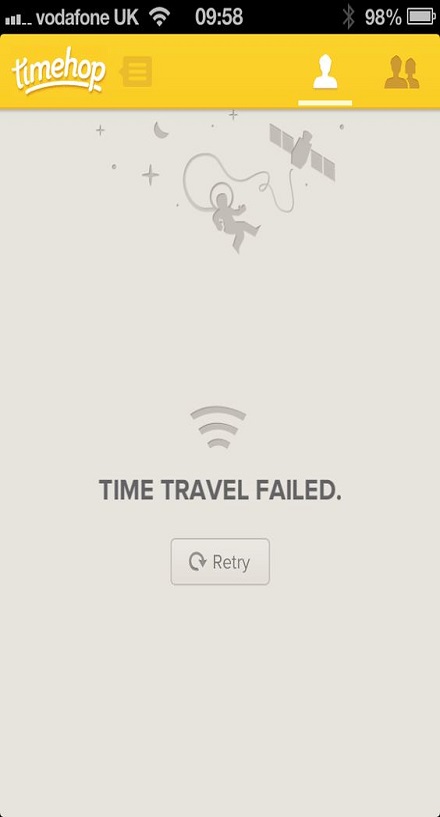

Supplement: Supplemental Information 1 — Use main file UI repair [file peerj-cs-10-2028-s001.zip › MUI Repair code and Data/null value/1.jpg]

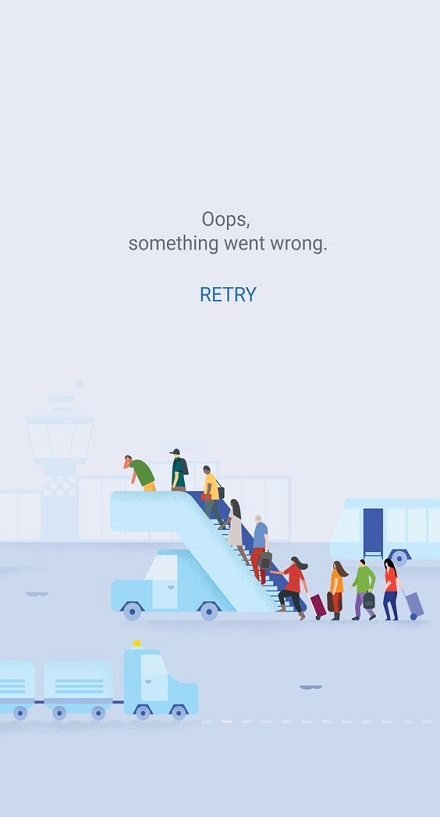

Supplement: Supplemental Information 1 — Use main file UI repair [file peerj-cs-10-2028-s001.zip › MUI Repair code and Data/null value/2.jpg]

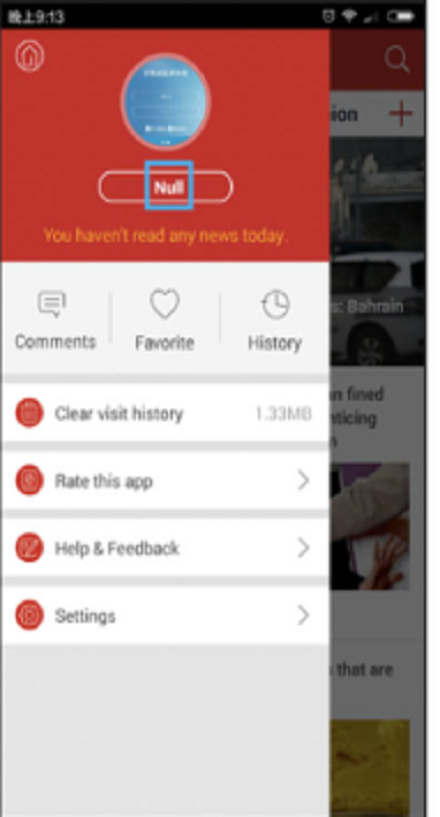

Supplement: Supplemental Information 1 — Use main file UI repair [file peerj-cs-10-2028-s001.zip › MUI Repair code and Data/null value/3.PNG]

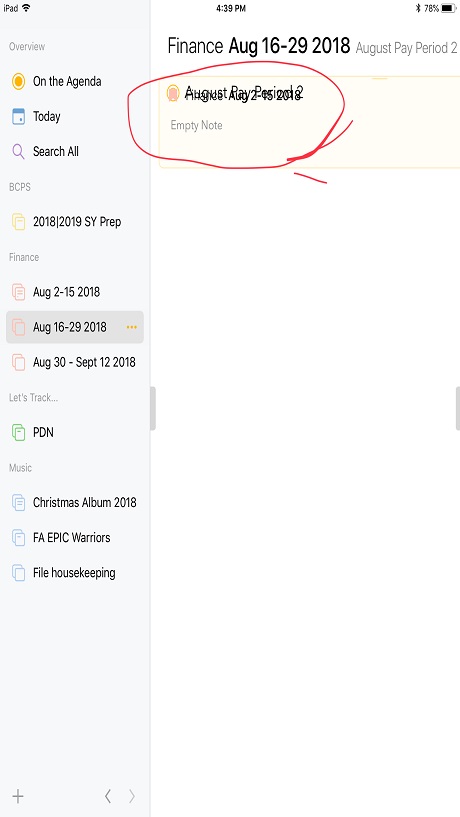

Supplement: Supplemental Information 1 — Use main file UI repair [file peerj-cs-10-2028-s001.zip › MUI Repair code and Data/text overlap/1.jpeg]

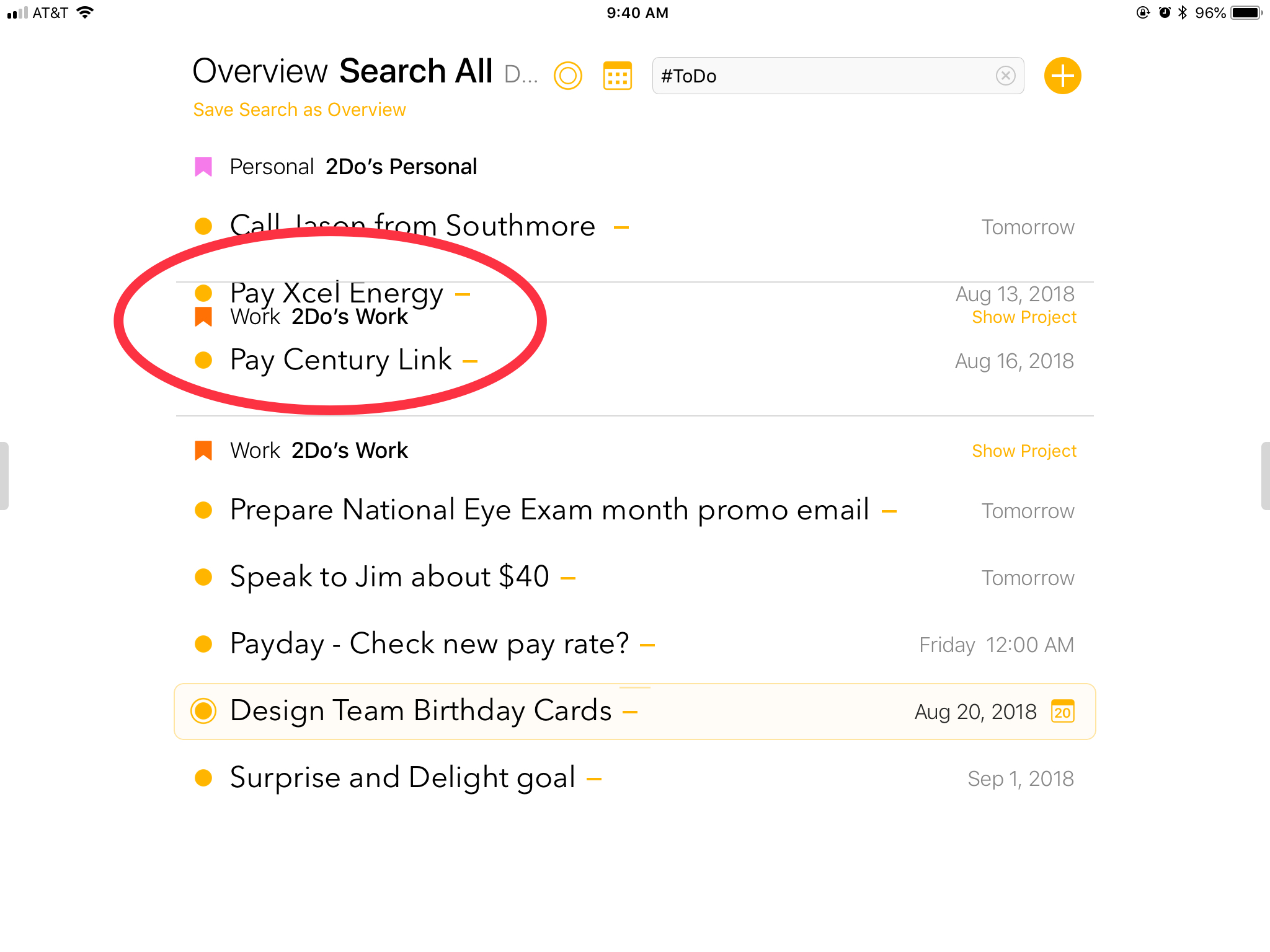

Supplement: Supplemental Information 1 — Use main file UI repair [file peerj-cs-10-2028-s001.zip › MUI Repair code and Data/text overlap/2.jpeg]

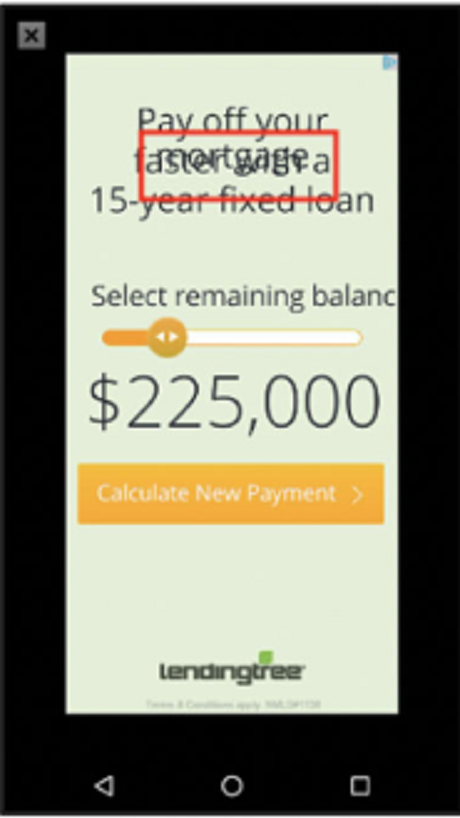

Supplement: Supplemental Information 1 — Use main file UI repair [file peerj-cs-10-2028-s001.zip › MUI Repair code and Data/text overlap/3.PNG]

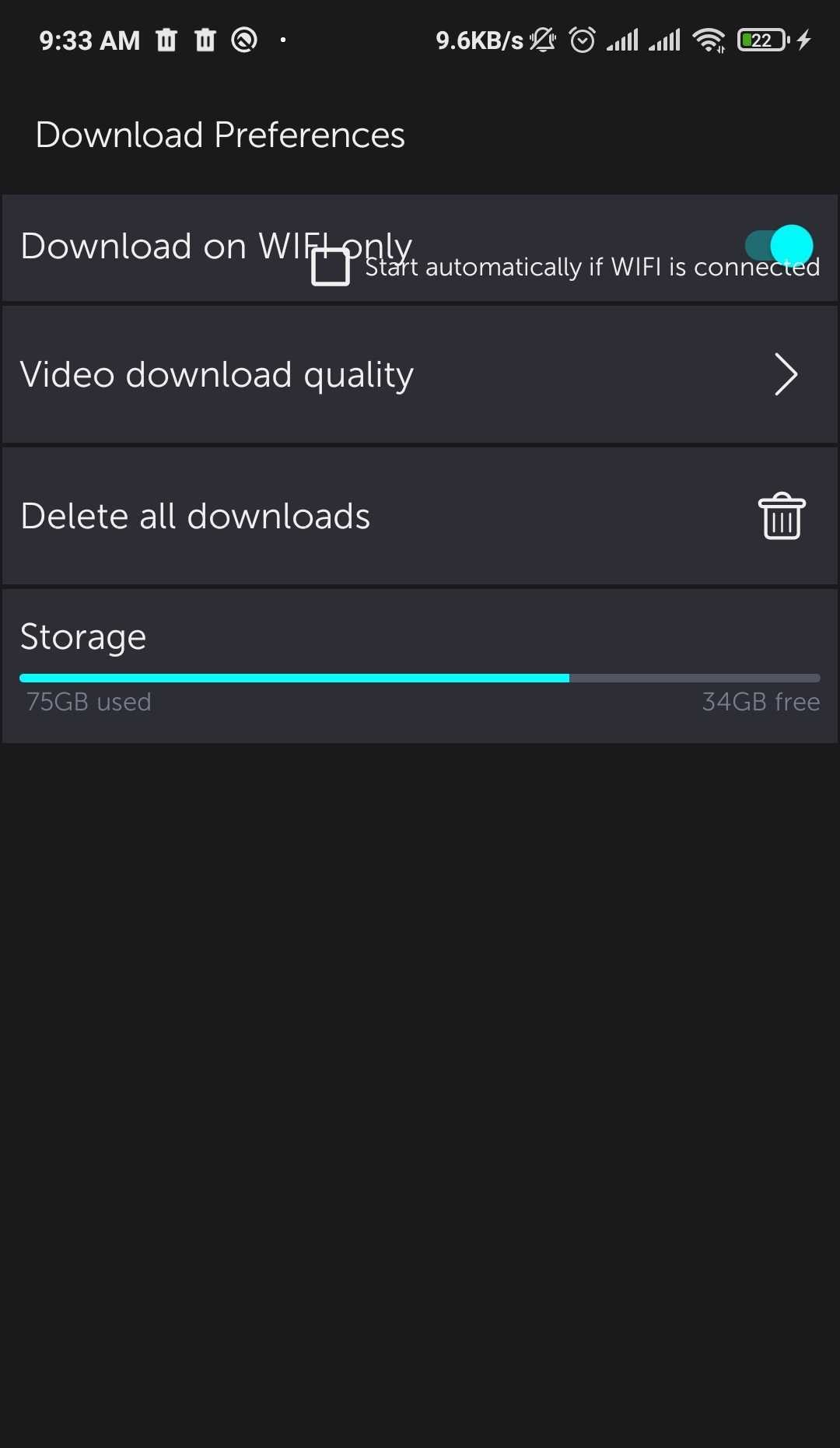

Supplement: Supplemental Information 1 — Use main file UI repair [file peerj-cs-10-2028-s001.zip › MUI Repair code and Data/UI images/1.jpg]

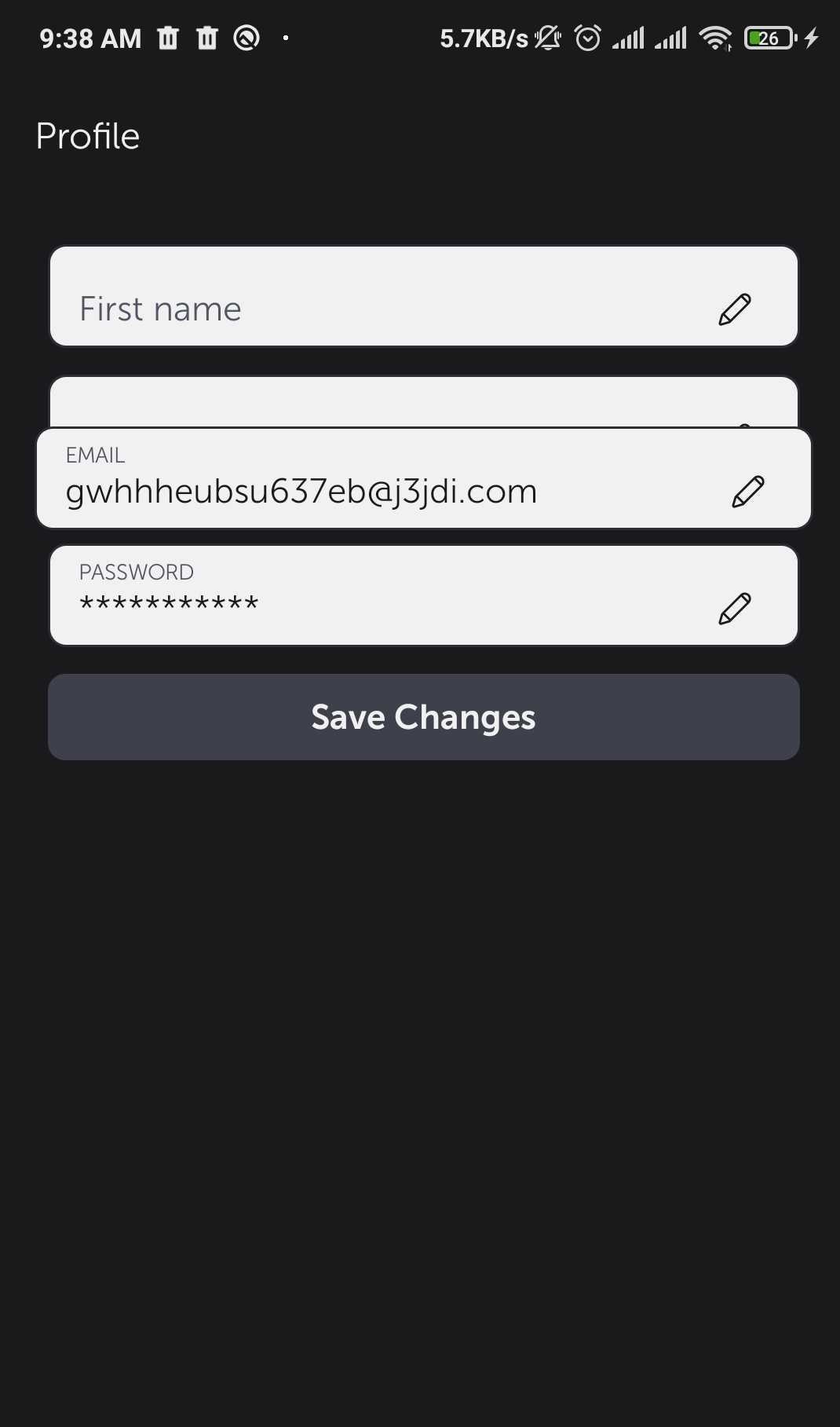

Supplement: Supplemental Information 1 — Use main file UI repair [file peerj-cs-10-2028-s001.zip › MUI Repair code and Data/UI images/3.jpg]

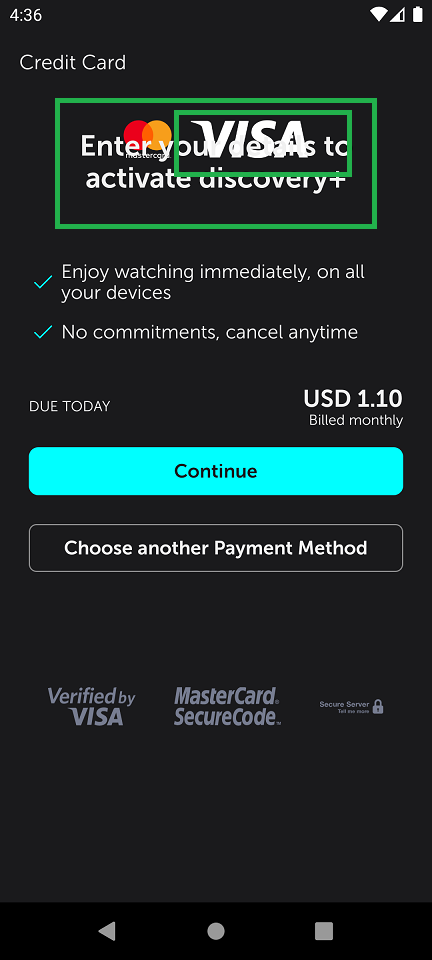

Supplement: Supplemental Information 1 — Use main file UI repair [file peerj-cs-10-2028-s001.zip › MUI Repair code and Data/UI images/Approach_1 last part.png]

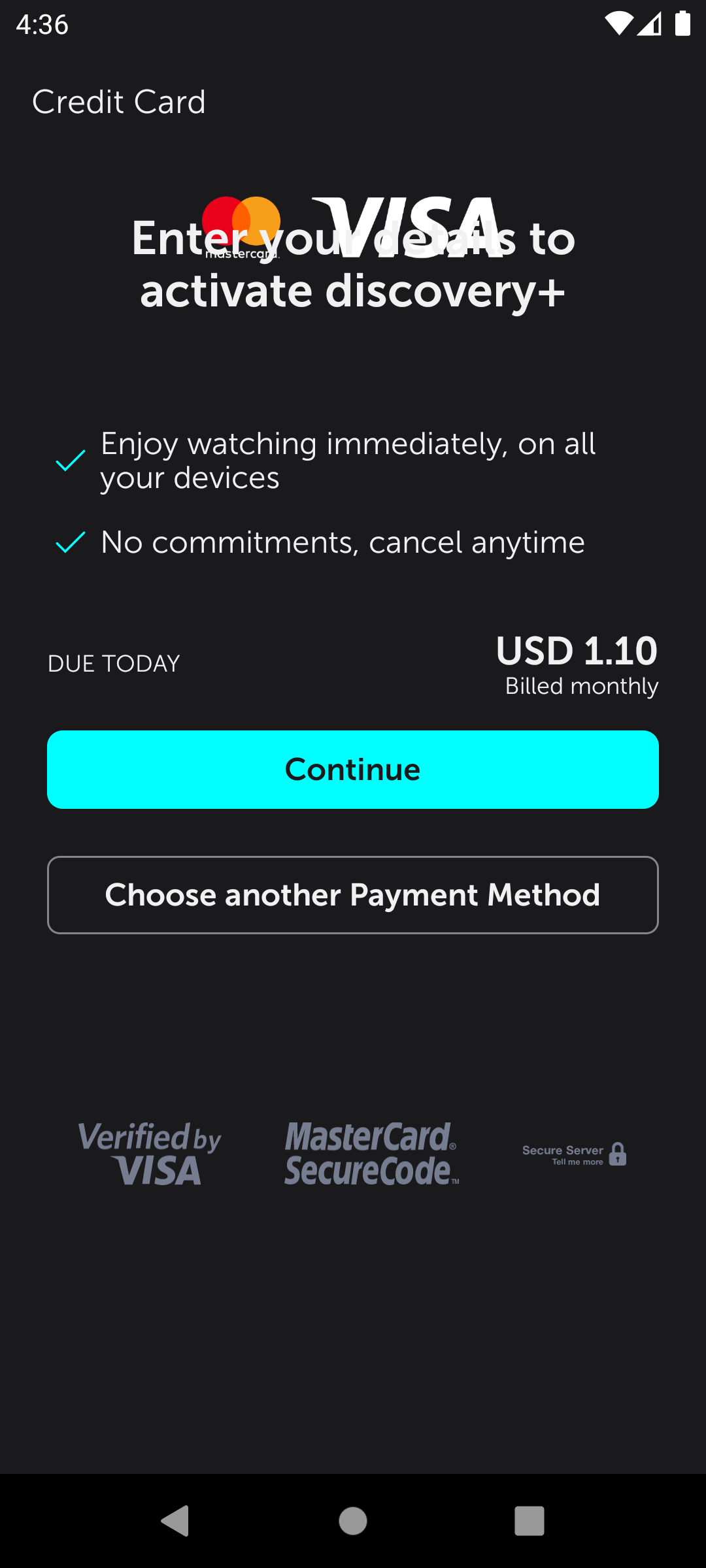

Supplement: Supplemental Information 1 — Use main file UI repair [file peerj-cs-10-2028-s001.zip › MUI Repair code and Data/UI images/New folder/Approach_1 last part.png]

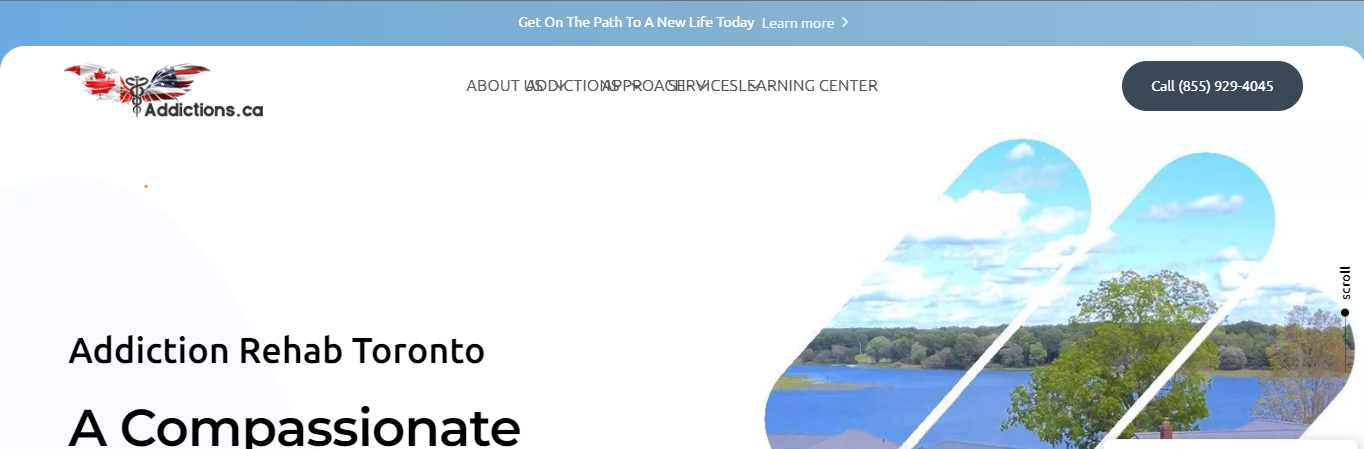

Supplement: Supplemental Information 1 — Use main file UI repair [file peerj-cs-10-2028-s001.zip › MUI Repair code and Data/UI images/Screenshot_10.png]

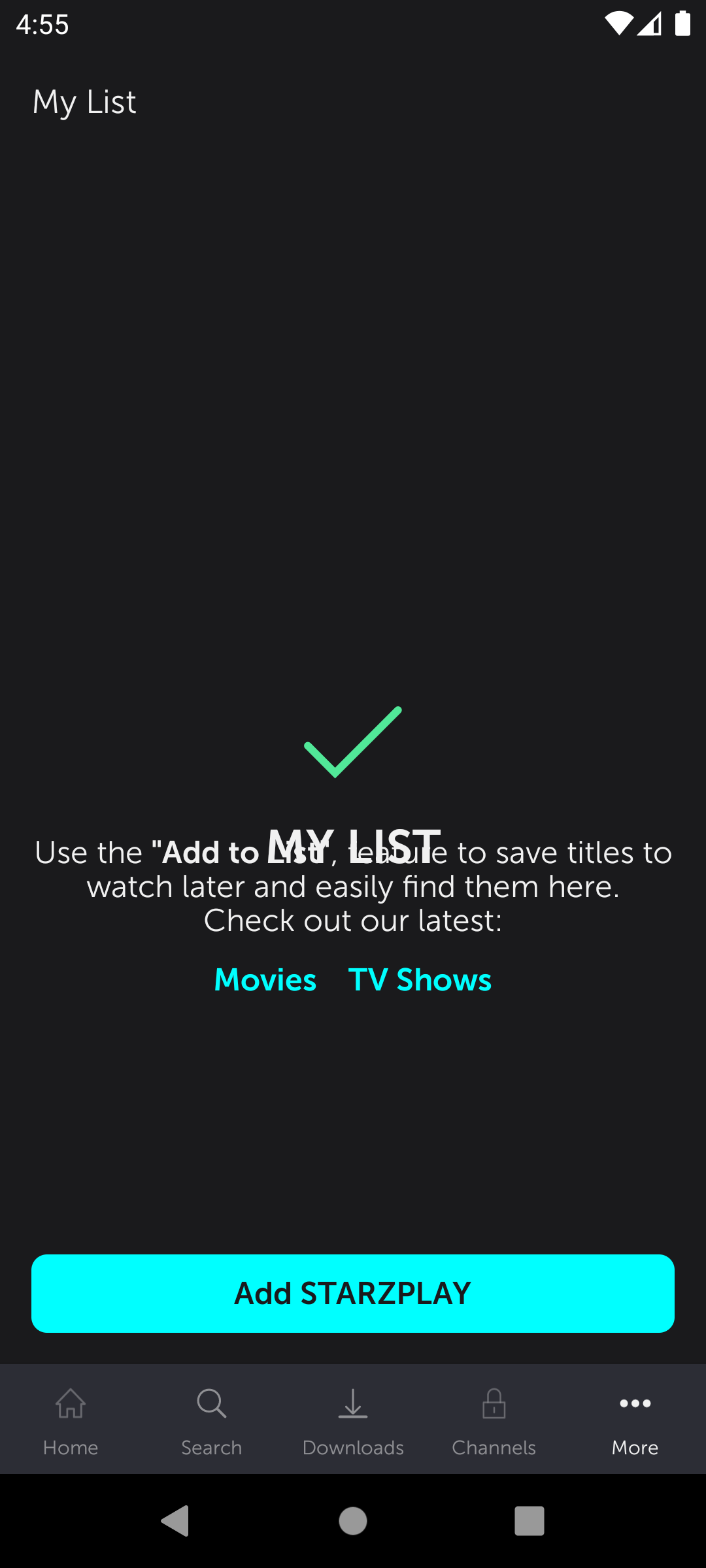

Supplement: Supplemental Information 1 — Use main file UI repair [file peerj-cs-10-2028-s001.zip › MUI Repair code and Data/UI images/Screenshot_1649850958.png]

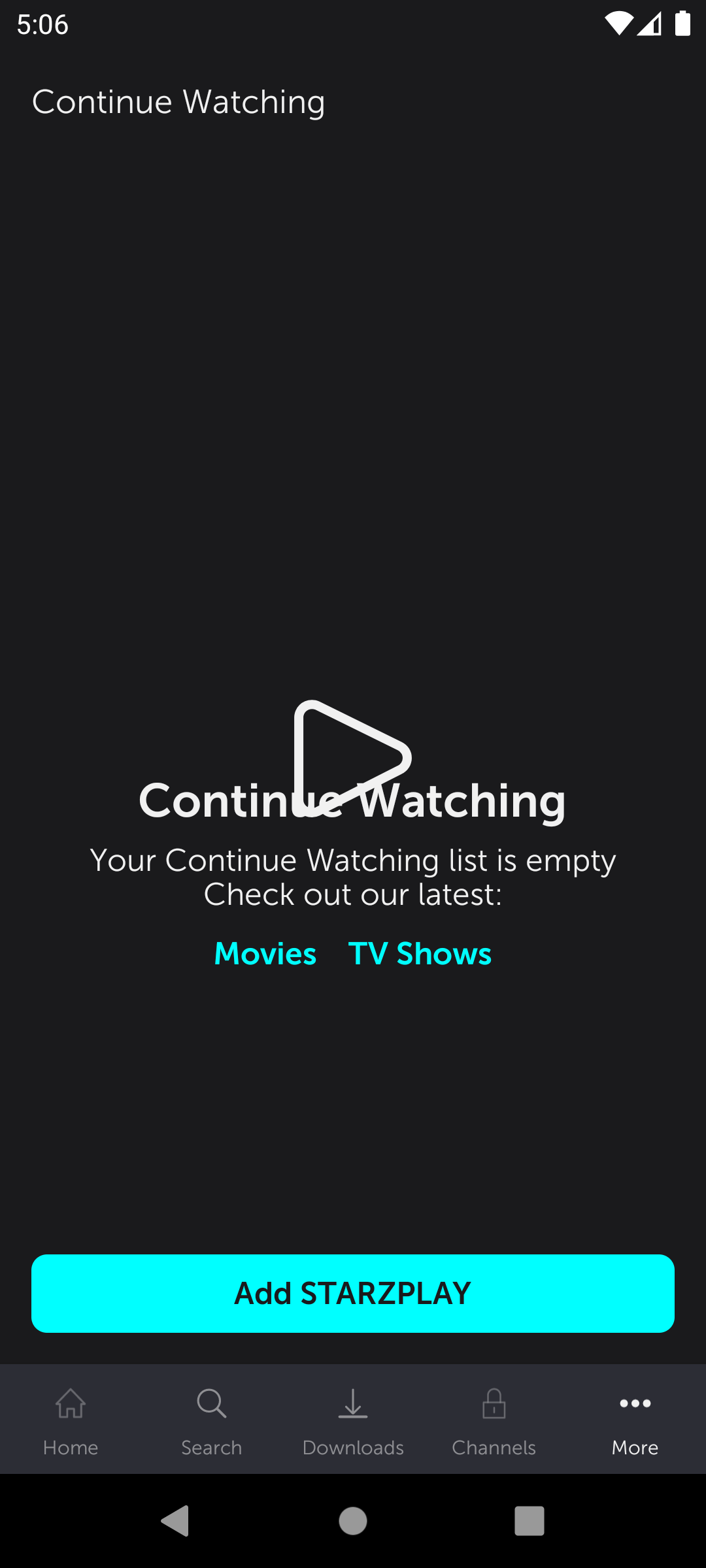

Supplement: Supplemental Information 1 — Use main file UI repair [file peerj-cs-10-2028-s001.zip › MUI Repair code and Data/UI images/Screenshot_1649851608.png]

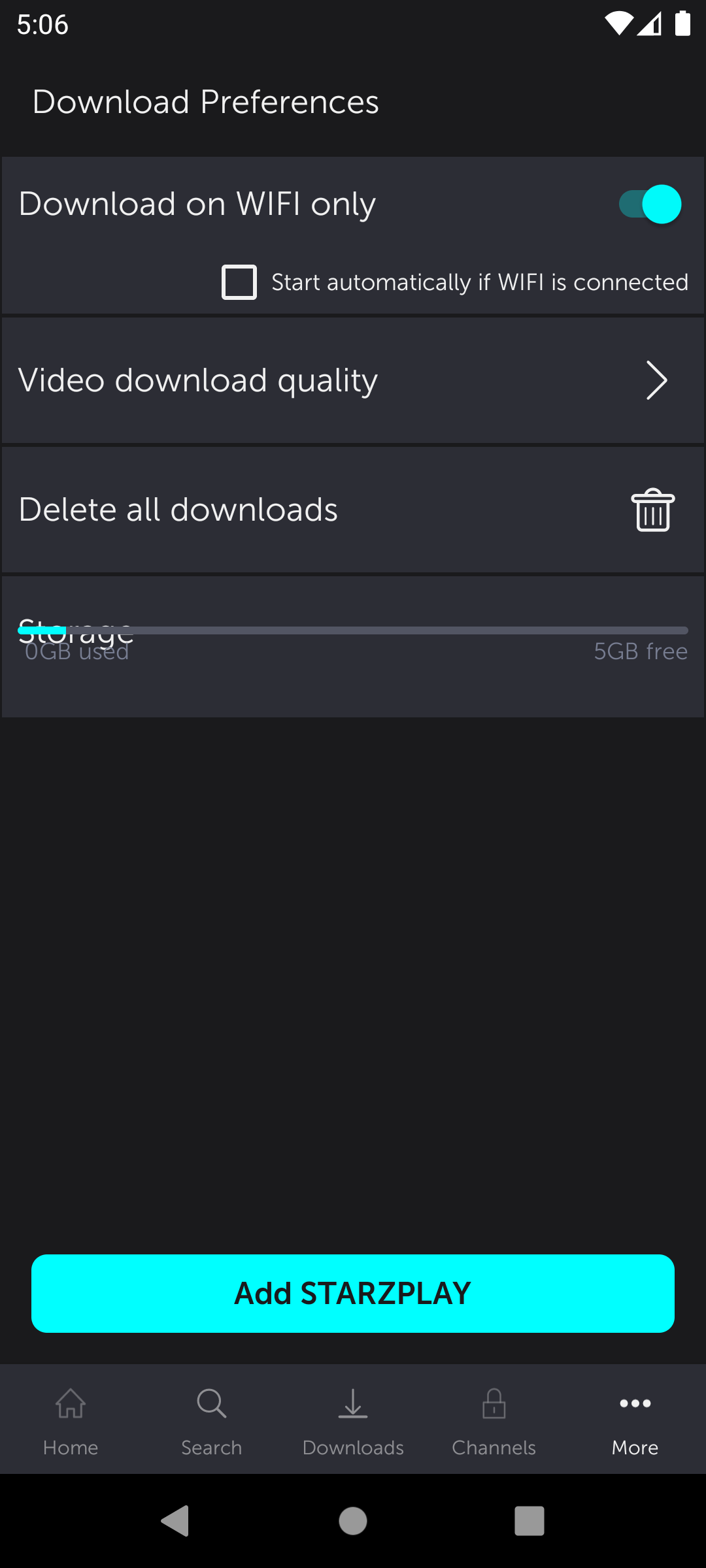

Supplement: Supplemental Information 1 — Use main file UI repair [file peerj-cs-10-2028-s001.zip › MUI Repair code and Data/UI images/Screenshot_1649851619.png]

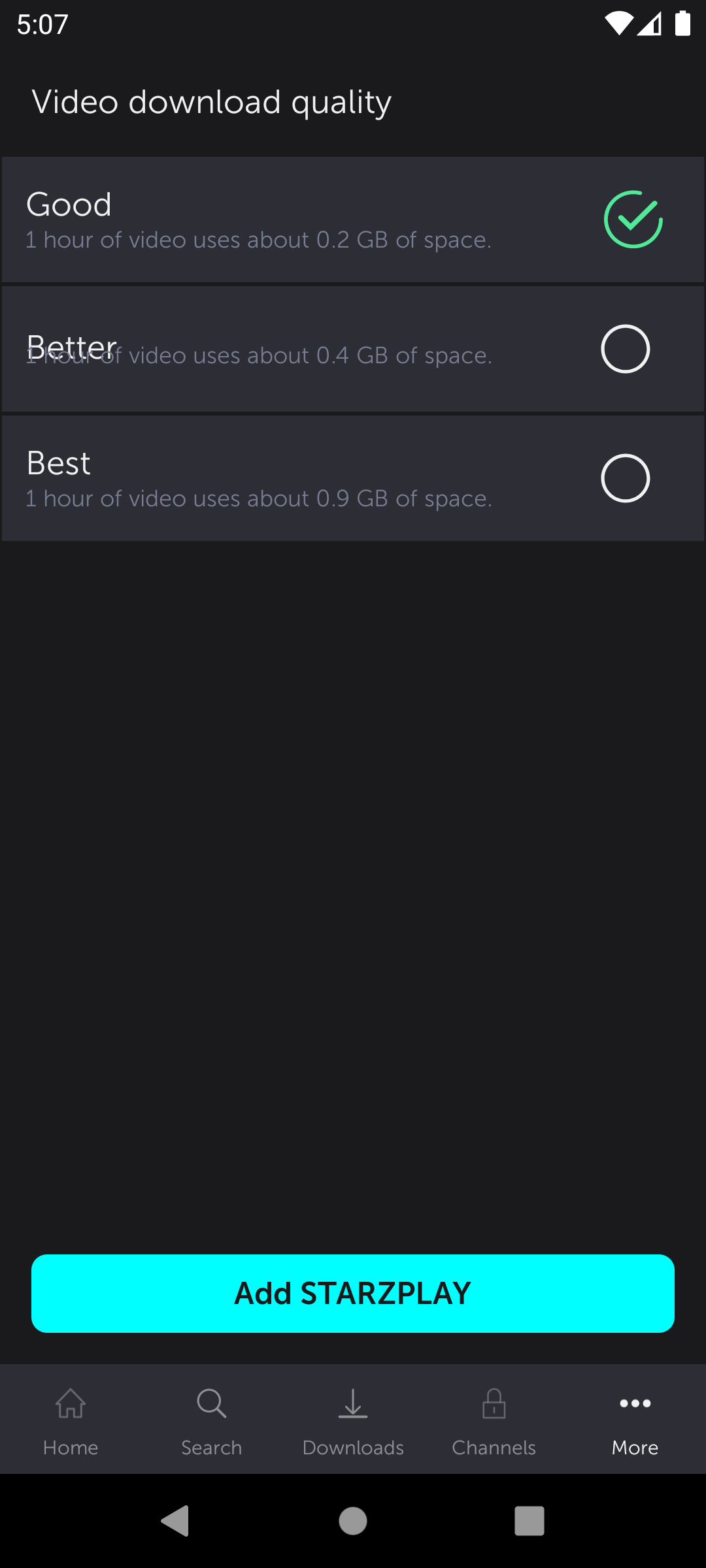

Supplement: Supplemental Information 1 — Use main file UI repair [file peerj-cs-10-2028-s001.zip › MUI Repair code and Data/UI images/Screenshot_1649851622.png]

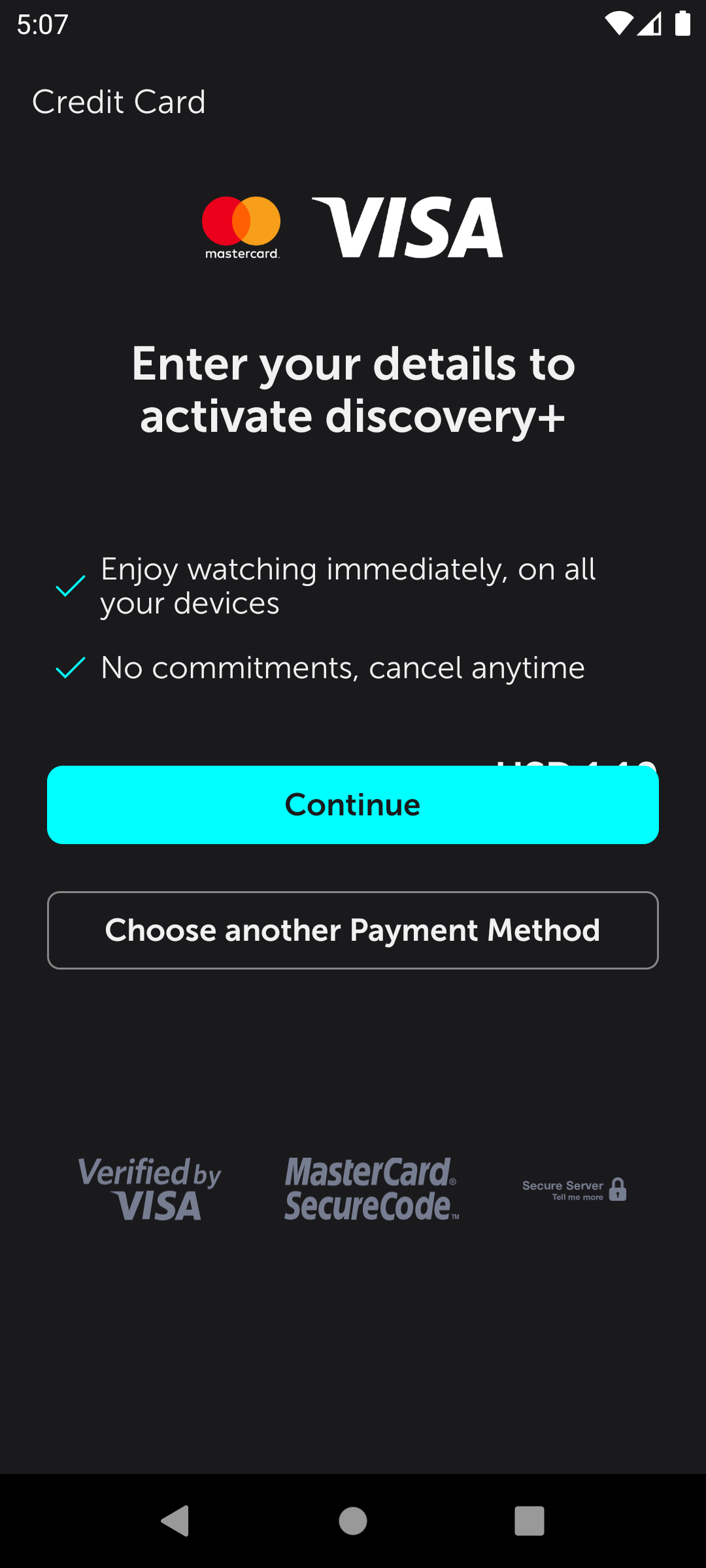

Supplement: Supplemental Information 1 — Use main file UI repair [file peerj-cs-10-2028-s001.zip › MUI Repair code and Data/UI images/Screenshot_1649851641.png]

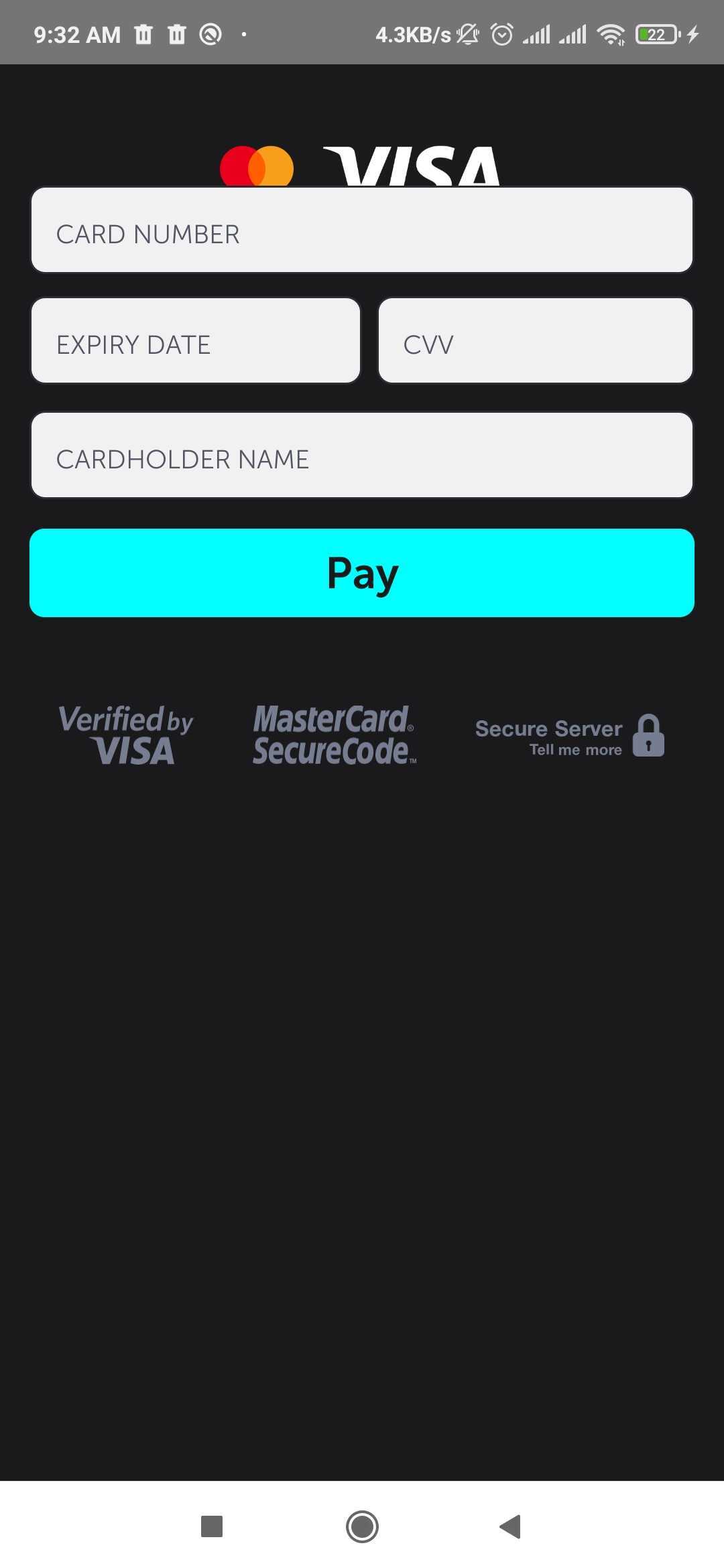

Supplement: Supplemental Information 1 — Use main file UI repair [file peerj-cs-10-2028-s001.zip › MUI Repair code and Data/UI images/Screenshot_2022-04-14-09-32-56-733_com.parsifal.starz.jpg]

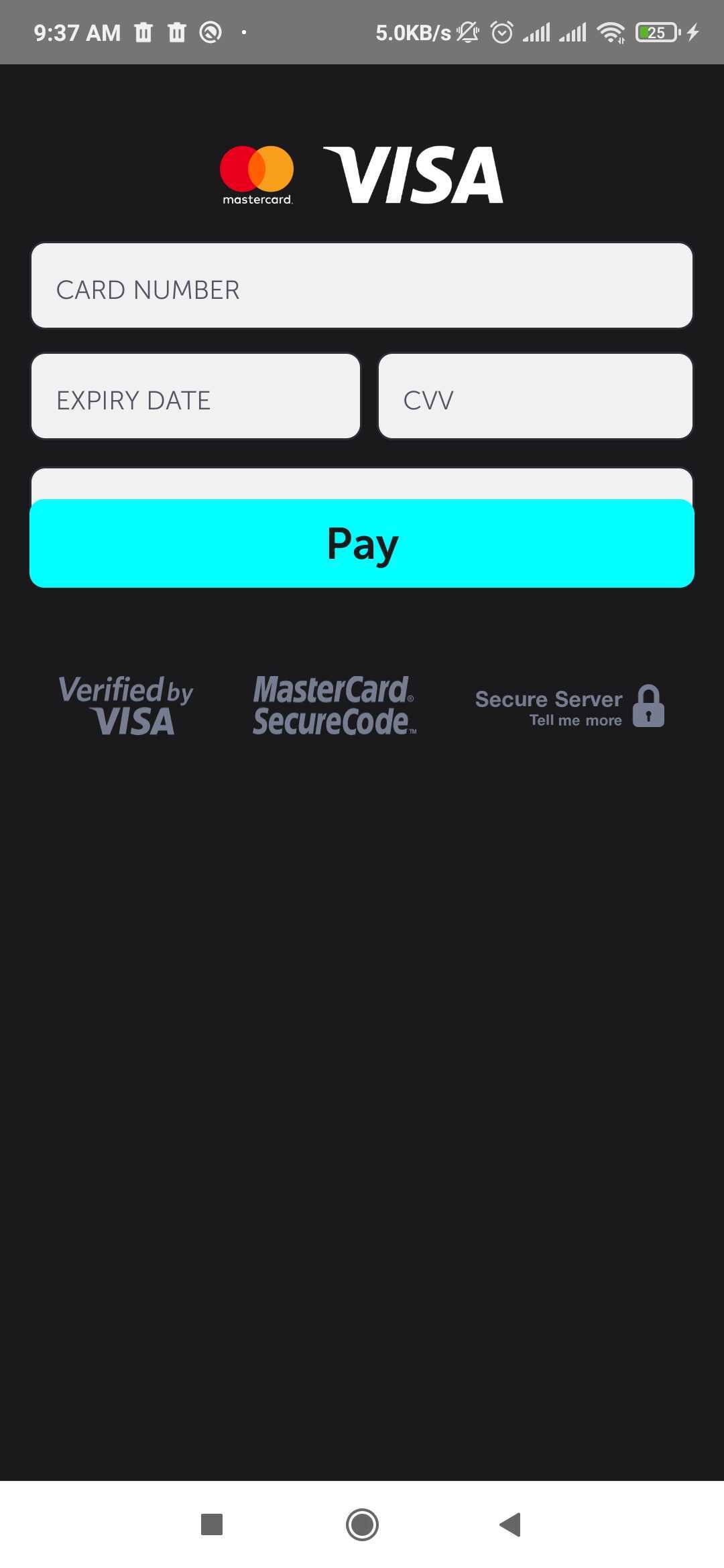

Supplement: Supplemental Information 1 — Use main file UI repair [file peerj-cs-10-2028-s001.zip › MUI Repair code and Data/UI images/Screenshot_2022-04-14-09-37-27-290_com.parsifal.starz.jpg]

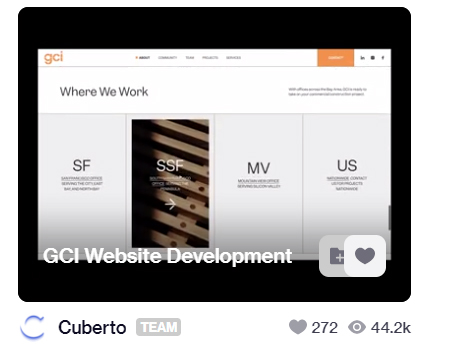

Supplement: Supplemental Information 1 — Use main file UI repair [file peerj-cs-10-2028-s001.zip › MUI Repair code and Data/UI images/Screenshot_22.jpg]

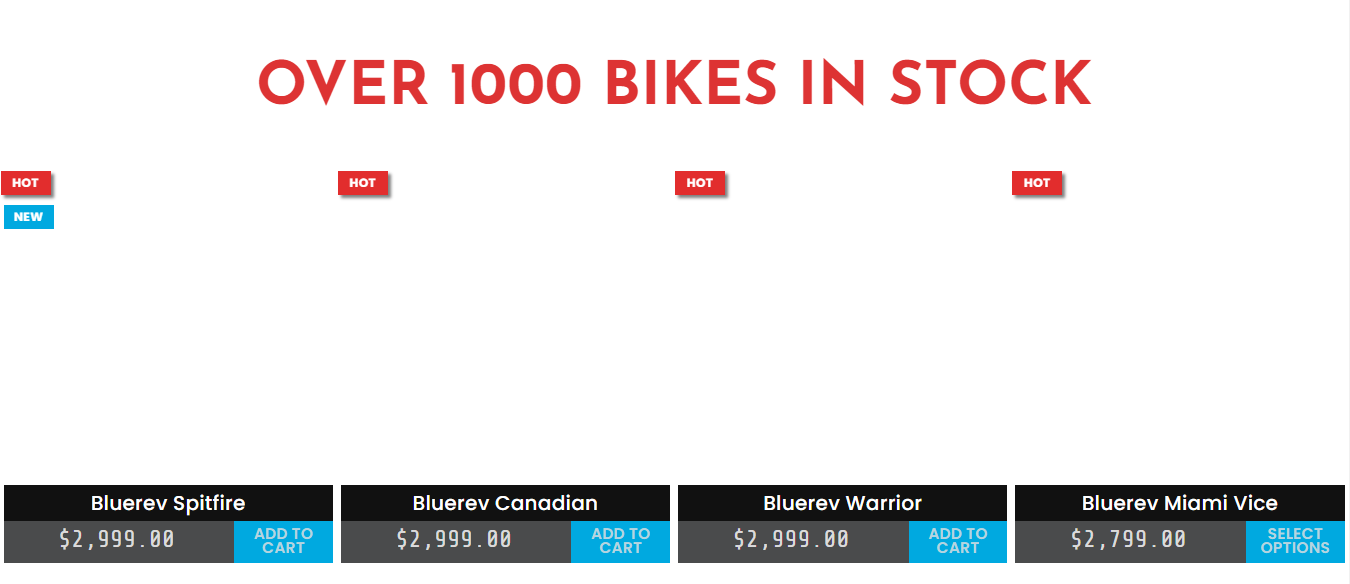

Supplement: Supplemental Information 1 — Use main file UI repair [file peerj-cs-10-2028-s001.zip › MUI Repair code and Data/UI images/Screenshot_29.png]

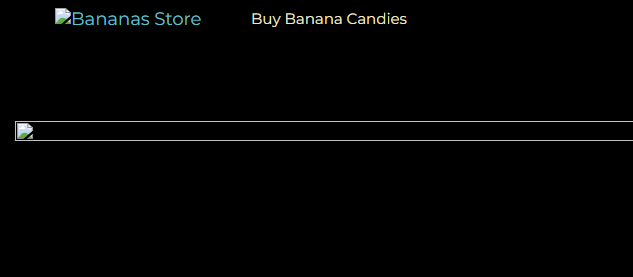

Supplement: Supplemental Information 1 — Use main file UI repair [file peerj-cs-10-2028-s001.zip › MUI Repair code and Data/UI images/Screenshot_9.png]
